# Explainable Rhythm-Resolved EEG for Retrospective Comparison of Psychiatric-Service Adults with Recorded Violent Offences and Non-Clinical Controls

**DOI:** 10.3390/diagnostics16213585

**Published:** 2026-10-30

**Authors:** Kubra Yildirim, Gulay Tasci, Irem Tasci, Mehmet Baygin, Burak Tasci, Kerim Uğur, Turker Tuncer, Sengul Dogan

**Affiliations:** 1 Department of Digital Forensics Engineering, Technology Faculty, Firat University, 23119 Elazig, Turkey; kubra.yildirim@firat.edu.tr (K.Y.); turkertuncer@firat.edu.tr (T.T.); 2 Department of Psychiatry, Elazig Fethi Sekin City Hospital, 23119 Elazig, Turkey; akcagulay01@gmail.com; 3 Department of Neurology, School of Medicine, Firat University, 23119 Elazig, Turkey; itasci@firat.edu.tr; 4 Department of Computer Engineering, Faculty of Engineering, Erzurum Technical University, 25050 Erzurum, Turkey; mehmet.baygin@erzurum.edu.tr; 5 Department of Electrical and Energy, Vocational School of Technical Sciences, Firat University, 23119 Elazig, Turkey; 6 Department of Psychiatry, Malatya Turgut Özal University, 44210 Malatya, Turkey; kerim.ugur@ozal.edu.tr

**Keywords:** electroencephalography, neurocriminology, HumanForensics, recorded violent offending, explainable artificial intelligence, DRLobish, psychiatric cohort, selection bias

## Abstract

**Background/Objectives**: This study examines whether rhythm-resolved EEG features separate two retrospective groups and whether those features can be traced to explicit rhythms and sensors. The groups are psychiatric-service adults with recorded violent offences and non-clinical controls; the design does not compare offenders with non-offenders. **Methods**: We analysed 2554 non-overlapping 15 s EEG epochs from 95 adults (51 controls; 44 recorded-offence participants). RhythmFluxPat generated 9216 rank-transition features. CWImRMR selected 352 features with full-cohort labels, and classifier selection was nested within 95 participant-held-out leave-one-subject-out (LOSO) folds. **Results**: Classifier-nested tkNN classified 2392/2554 epochs (93.66%) and 93/95 participants (97.89%), and an independent check reproduced both confusion matrices. A nested support-vector machine (SVM) gave similar values (94.71% of epochs; 93/95 participants). In the comparative evaluation of four feature selectors, CWImRMR remained the best branch (93.85%), and a 44-feature subset reached 91.11%. DRLobish linked the selected features to delta and beta-to-gamma routes in the recorded-offence-assigned panel and to theta and alpha-to-theta routes in the control-assigned panel. **Conclusions**: RhythmFluxPat provides traceable rank-persistence and rank-exchange coordinates. The reported accuracies are optimistic internal estimates. Fully nested replication with matched psychiatric controls is required before any claim of generalisation or biomarker validity.

## 1. Introduction

Neurocriminology combines neuroscience and criminology to examine associations between brain function, antisocial behaviour and criminal conduct [1]. The field does not replace psychological, clinical, social or situational explanations of violence. Instead, neurobiological findings are evaluated as one part of a broader behavioural framework. This distinction is important because violent offending is heterogeneous. Individuals with recorded violent offences may differ in psychiatric diagnosis, medication status, substance exposure, cognitive state and social background. A single neural pattern should therefore not be expected to explain violent behaviour across all individuals or populations.

Electroencephalography (EEG) provides a non-invasive method for the assessment of brain activity with high temporal resolution. Early clinical studies did not establish a direct relationship between an abnormal EEG tracing and violent criminal behaviour [2]. Later quantitative EEG studies reported differences in spectral activity among homicidal men with antisocial personality disorder [3]. Qualitative and quantitative EEG abnormalities were also reported in violent offenders with antisocial personality disorder [4]. Differences between psychopathic and non-psychopathic violent offenders were subsequently examined through EEG recordings [5]. Frontal alpha asymmetry was also associated with callous-unemotional traits in an imprisoned violent-offender sample [6].

Recent studies have expanded this field beyond conventional spectral analysis. Task-related oscillatory activity was examined during laboratory-induced reactive aggression [7]. Reduced temporal stability of resting EEG networks was associated with higher trait aggression [8]. Changes in oscillatory activity and frontal synchrony were evaluated after high-definition transcranial direct-current stimulation in violent offenders with substance dependence [9]. EEG microstate abnormalities were also reported among violent patients with schizophrenia [10]. More recently, resting-state EEG markers were associated with violence risk in patients with major depressive disorder [11].

These studies show that aggression-related EEG findings depend strongly on the investigated population and experimental condition. Antisocial personality disorder, psychopathy, schizophrenia, depression and substance dependence represent distinct clinical contexts [10,11]. Resting-state recordings, reactive-aggression tasks and stimulation protocols also measure different aspects of brain activity [7,8,9]. Therefore, findings from one forensic or clinical group cannot be directly generalised to all individuals with a history of violent offending.

The interpretation of EEG findings in neurocriminology requires additional caution. Oscillatory activity can be affected by age, vigilance, medication, psychiatric state, substance exposure, attention and recording quality. Similar EEG patterns may therefore arise from different biological or clinical conditions. An EEG classifier should not be interpreted as a direct measure of dangerousness, criminal intent or future behaviour. Its output should instead be considered a cohort-specific statistical association that requires independent validation.

The present recordings were obtained during a common meditation-video condition. An early pilot study reported EEG changes during a mindfulness-based relaxation technique [12]. More recent work associated mindfulness meditation with broad changes in theta, alpha and beta amplitudes [13]. Meditation-related oscillatory changes were also reported independently of general arousal effects [14]. However, passive viewing of a meditation video does not confirm that every participant reached the same cognitive or meditative state. The video was therefore treated only as a standardised audiovisual context. It was not considered a therapeutic intervention or a verified mindfulness manipulation.

A major challenge in EEG-based classification is the balance between predictive performance and interpretability. High-dimensional models may achieve strong internal performance, but their decisions are often based on anonymous feature indices or latent representations. Such outputs provide limited information about the EEG rhythms and sensor locations that contribute to classification. This limitation is especially important in neurocriminology because model outputs may be socially consequential and easily overinterpreted.

The present study introduces RhythmFluxPat, a rhythm-resolved EEG feature extractor. It converts each EEG epoch into directed source–rhythm–target coordinates. Each coordinate retains its rhythm layer, source channel and temporally later target channel. Cumulative-weighted minimum redundancy maximum relevance selection (CWImRMR) reduces the feature space, and the t-algorithm-based k-nearest-neighbour framework (tkNN) classifies the selected inputs. DRLobish decodes the retained coordinates into readable rhythm routes. RhythmFluxPat-XFE denotes the complete extraction, selection, classification and interpretation workflow.

The primary research question is whether the present cohort contains a separable EEG pattern under participant-held-out classifier testing. The second question is whether the coordinates that support this separation can be traced to explicit rhythm and channel routes. The study focuses on retrospective group separation. It does not estimate future offending, recidivism, dangerousness, moral responsibility or criminal intent. The recorded-offence group was recruited from a psychiatry service, whereas controls had no prior clinical diagnosis. The study therefore compares psychiatric-service adults with recorded violent offences and non-clinical adults. It does not compare offenders with non-offenders.

### 1.1. Neurocriminology and Related Work

The available neurocriminology literature does not support a universal EEG signature of violent offending. Earlier studies mainly focused on visible EEG abnormalities and broad spectral differences [2,4]. Later studies examined more specific clinical and behavioural subgroups. Psychopathy-related differences were evaluated among violent offenders [5], while frontal alpha asymmetry was investigated in relation to callous-unemotional traits [6]. These studies provided useful evidence, but their participant groups and clinical characteristics differed substantially.

The focus of the field has also expanded towards dynamic EEG properties. Oscillatory responses during reactive aggression were investigated under a controlled laboratory task [7]. Resting-network stability was examined in relation to trait aggression rather than documented criminal behaviour [8]. Clinical studies assessed oscillatory and synchronisation patterns in violent offenders with substance dependence [9], violent patients with schizophrenia [10] and patients with major depressive disorder who had different levels of violence risk [11].

These findings should not be treated as equivalent observations. Trait aggression, recorded violent offending and clinically assessed violence risk represent different outcomes. The associated samples also differ in diagnosis, treatment status, substance exposure and recruitment setting. A useful computational framework should therefore retain detailed rhythm and sensor information. A single global spectral value may not adequately represent this heterogeneity.

High-dimensional EEG representations also require disciplined feature selection. Minimum redundancy maximum relevance was developed to preserve class relevance while reducing redundancy among selected variables [15]. Neighbourhood component analysis provides a supervised approach based on local neighbourhood structure [16]. ReliefF evaluates feature relevance through differences between neighbouring observations from the same and opposite classes [17]. These methods offer different views of feature importance and may therefore retain different EEG coordinates.

The k-nearest-neighbour classifier provides a transparent decision rule based on local sample similarity [18]. The tkNN framework extends this principle through the systematic evaluation of neighbour count, distance metric and distance weighting [19]. This structure is suitable for an explainable feature-engineering workflow because the final decision remains linked to the selected input coordinates.

Feature selection alone does not provide a complete explanation. A selected numerical index must be connected to the original EEG representation. Directed Lobish was developed to convert selected EEG features into explicit rhythm and channel statements [20]. DRLobish further supports source–rhythm–target interpretation through a structured symbolic EEG language. These approaches provide a reversible link between feature selection and sensor-level reporting.

Neurocriminology has progressed from general biological associations towards questions of prediction, explanation and responsible use [1]. However, prospective study designs and incremental validation remain limited in violence neuroprediction research [21]. The present study therefore addresses a narrower objective. Retrospective group separation is evaluated, and the selected EEG coordinates are reported through an auditable interpretation framework.

A previous ChMinMaxPat study applied explainable EEG feature engineering to violence- and stress-related classification [22]. The present study extends this research direction through rhythm-resolved and temporally directed channel transitions. Neurocriminology defines the scientific domain of the study. HumanForensics defines the computational requirements for traceability, participant-disjoint testing, readable outputs and explicit restrictions on use.

HumanForensics is used here as a study-specific operational label for three computational requirements: transparent feature provenance, participant-disjoint evaluation and explicit safeguards against individual forensic interpretation. It is not presented as an established external diagnostic standard.

### 1.2. Literature Gaps

Three main limitations remain in explainable EEG studies of aggression and recorded violent offending. First, EEG recordings commonly produce several segments from each participant. Random placement of these segments in both training and test sets may allow participant-specific patterns to appear on both sides of the evaluation. The resulting performance may reflect participant recognition rather than group-level generalisation. Validation procedures for small biomedical data sets must therefore preserve participant independence [23]. Small sample sizes can also produce substantial uncertainty in cross-validation estimates [24].

Second, selected EEG features are often reported only as numerical indices. This approach limits physiological interpretation because the original rhythm and sensor coordinates remain hidden. A selected feature should be reversible to its rhythm layer, source sensor and target sensor. Without this relationship, the explanation remains incomplete.

Third, feature selection and model optimisation may bias the reported performance. General feature-selection principles require a clear separation between variable selection and final model assessment [25]. When cross-validation is used both for model selection and performance reporting, the estimated error may become optimistic [26]. Strong performance within a single development cohort should therefore not be interpreted as evidence of a validated biomarker.

The development analysis nested only classifier selection; feature ranking and prefix selection used all participants. Such selection can inflate accuracy. The fixed-panel accuracies are therefore reported as optimistic estimates. Reversible rhythm and sensor coordinates provide feature traceability.

### 1.3. Theoretical Basis of RhythmFluxPat

Conventional EEG rhythm analysis commonly summarises activity through average power or global spectral values. These measures are informative, but short-term temporal organisation and sensor-level transitions may be lost. RhythmFluxPat was designed to preserve this information within a deterministic feature space.

The model represents EEG activity through five conventional frequency bands: delta, theta, alpha, beta and gamma. Five within-rhythm layers describe temporal channel transitions within the same rhythm. Four additional layers describe ordered cross-rhythm relationships: alpha-to-theta, gamma-to-alpha, theta-to-beta and beta-to-gamma.

Each rhythm layer is transformed into a directed channel-transition matrix. The channel at a given temporal rank is treated as the source. The channel at the corresponding rank after a short temporal delay is treated as the target. Several millisecond-scale delays are used to represent short-term transitions at different temporal intervals.

The direction of each route is defined by temporal order within the algorithm. It does not represent anatomical connectivity, effective connectivity or causal neural influence. A source-to-target route means that two channels occupied corresponding ranks at ordered time points within a rhythm map. RhythmFluxPat should therefore be interpreted as a rank-resolved temporal transition descriptor.

Ranking turns each time sample into a permutation of the 32 channels. Without value weights, each sample adds one permutation matrix to the transition count, so the count matrix is doubly stochastic (Appendix C). Its diagonal measures how often channels keep their amplitude rank (rank persistence). Its off-diagonal entries measure how often one channel takes the rank previously held by another channel (rank exchange). At the lags used here, the delta and theta layers mainly describe rank persistence, because 1–8 samples cover at most 12.5% of a delta cycle and 25% of a theta cycle. RhythmFluxPat is therefore described as a rank-persistence and rank-exchange descriptor. It does not measure signal propagation.

Each rhythm layer contains all ordered source–target channel pairs, including self-transitions. A deterministic vectorisation order is used. Every feature index can therefore be decoded into three components: rhythm layer, source channel and target channel. This reversibility forms the basis of the subsequent DRLobish interpretation.

### 1.4. Neurocriminology and HumanForensics Model

The proposed framework combines the scientific perspective of neurocriminology with the computational principles of HumanForensics. Neurocriminology defines the brain–behaviour research question. HumanForensics provides the requirements for traceability, explainability and responsible interpretation. Within this framework, each computational output can be linked to its original EEG coordinate.

RhythmFluxPat produces a high-dimensional but reversible EEG representation. Each feature corresponds to a defined rhythm layer, source channel and target channel. CWImRMR reduces this feature space by retaining coordinates with high class relevance and limited redundancy. The selected feature panel is then classified with tkNN. Finally, DRLobish converts the retained feature indices into readable source–rhythm–target expressions.

**Figure 1:**
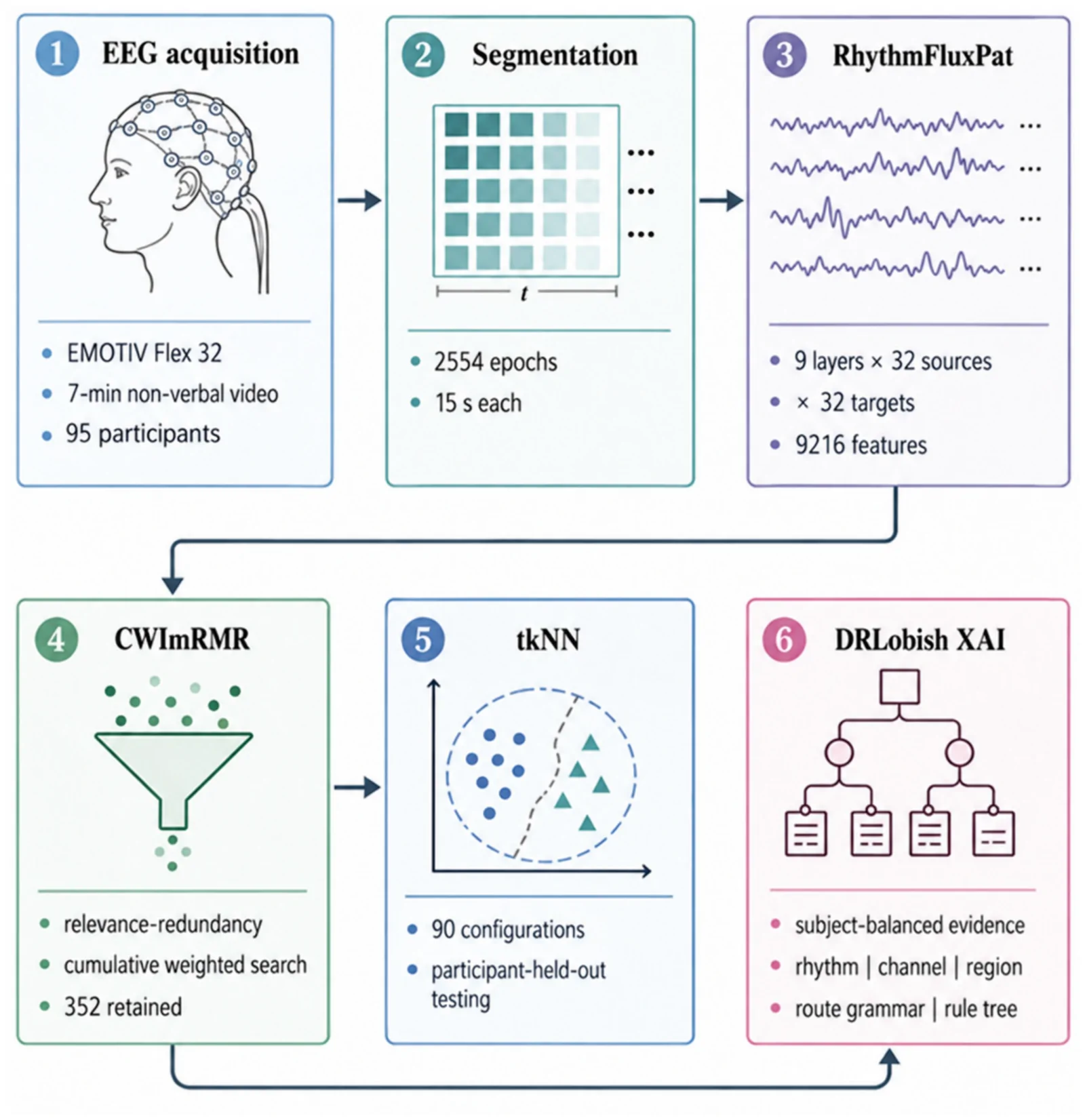
RhythmFluxPat–CWImRMR–tkNN–DRLobish workflow. A 15 s EEG epoch is converted into nine 32 × 32 transition layers. The resulting feature space is reduced through CWImRMR and classified under participant-held-out tkNN testing. Each retained coordinate is decoded into its rhythm layer, source channel, target channel and reporting grammar.

The complete RhythmFluxPat–CWImRMR–tkNN–DRLobish workflow is presented in Figure 1. As shown in Figure 1, a 15 s EEG epoch is first transformed into nine rhythm-specific transition layers. The resulting 9216 features are ranked and reduced through CWImRMR. The selected coordinates are evaluated under participant-held-out tkNN testing. The retained feature indices are then decoded through DRLobish.

Figure 1 also shows the separation between the prediction and interpretation stages. RhythmFluxPat, CWImRMR and tkNN form the feature-engineering and classification path. DRLobish forms the interpretation path. DRLobish is applied after feature selection and does not create additional classifier inputs. It also does not modify the stored predictions.

This architecture provides an auditable path from the original EEG epoch to the final participant-level output. An EEG epoch is first linked to a feature vector. Each selected feature is then linked to a rhythm and channel route. The resulting route can subsequently be linked to a participant-balanced class summary. Therefore, no anonymous latent representation is required for the interpretation stage.

The HumanForensics framework also defines explicit limits on model use. Computational traceability does not establish causality, external validity or suitability for automated forensic decisions. The identified routes represent cohort-level sensor-space associations. They do not represent anatomical pathways, effective connectivity or causal neural influence. The framework does not infer dangerousness, future conduct, criminal intent, moral responsibility or legal responsibility.

### 1.5. Contributions and Innovations

The study makes three main contributions.

- First, an explainable rhythm-resolved EEG feature-engineering framework is introduced for neurocriminology. Each EEG epoch is represented through directed and reversible channel-transition coordinates.
- Second, classifier fitting and tuning use participant-disjoint folds. A second classifier family and a comparative feature-selection analysis test the robustness of the result. The accuracies are reported as optimistic estimates, not as generalisation performance.
- Third, the selected feature panel is converted into readable source–rhythm–target routes. This process separates predictive performance from neurophysiological interpretation and forensic use.

The principal technical innovations are summarised as follows:

- RhythmFluxPat generates five within-rhythm and four cross-rhythm-transition layers from robustly normalised Hilbert rhythm maps.
- Each feature retains a deterministic relationship with its rhythm layer, source channel and target channel.
- CWImRMR evaluates cumulative mRMR prefixes instead of fixing the number of selected features in advance.
- tkNN evaluates transparent nearest-neighbour configurations based on neighbour count, distance metric and distance weighting.
- DRLobish produces participant-balanced route summaries through reversible channel-level and regional representations.
- A lower-capacity surrogate model is used as an explanation audit and is not presented as a replacement for the primary classifier.
- Together, these components provide an auditable path from EEG activity to participant-level classification and rhythm-route interpretation.

## 2. Materials and Methods

### 2.1. Participants, Task and EEG Acquisition

The study cohort consisted of 95 adults. The control group included 51 participants, comprising 6 women and 45 men. Their ages ranged from 19 to 42 years, with a mean age of 30.5 ± 5.8 years. The recorded-offence group included 44 participants, comprising 4 women and 40 men. Their ages ranged from 22 to 67 years, with a mean age of 44.5 ± 11.3 years. Participants in the recorded-offence group were recruited from the Psychiatry Service of Firat University Hospital. Information on the recruitment source of the control group was unavailable. The recorded-offence group therefore combines psychiatric-service status with recorded-offence status.

The control group consisted of participants with no history of violent offences identified in the official records examined for the study and no prior clinical diagnosis. The recorded-offence group consisted of participants with a recorded violent offence. Information on offence subtype, offence date, judicial status and the interval between the offence and EEG acquisition was unavailable. Accordingly, the group definition represents recorded-offence status and does not provide a direct measure of current aggression or future violent behaviour. The contrast is therefore between psychiatric-service adults with recorded violent offences and non-clinical adults, not between offenders and non-offenders.

The demographic characteristics and evaluable EEG data are summarised in Table 1. As shown in Table 1, the groups differed in mean age and in the range of usable epochs contributed by each participant.

Age was not included as a predictor. The groups differed in mean age by 14 years (30.5 versus 44.5 years; Table 1). This imbalance could contribute to EEG separation; excluding age from the predictor matrix does not remove age-related signal differences. Recomputed from the group summaries (30.51 ± 5.85 versus 44.48 ± 11.33 years), the difference was 13.97 years (Welch t(62.2) = 7.37, *p* = 4.7 × 10^−10^; 95% CI 10.18–17.76 years; Hedges’ g = 1.57). Sex proportions did not differ (Fisher exact *p* = 0.75). Age is therefore a strong competing explanation for any group separation (Section 4.4).

As presented in Table 1, 2554 non-overlapping 15 s EEG epochs were included in the analysis. The control group contributed 1430 epochs, and the recorded-offence group contributed 1124 epochs. The median number of epochs per participant was 28 in both groups. However, control participants contributed between 28 and 30 epochs, whereas participants in the recorded-offence group contributed between 7 and 28 epochs.

The experimental condition consisted of a seven-minute excerpt from a non-verbal audiovisual stimulus featuring relaxing natural scenery accompanied by calming background music [27]. Information on the exact start and end points of the excerpt, presentation medium, display settings, and sound level was unavailable. EEG recordings were performed in a quiet environment to minimise the influence of external environmental stimuli, and during the recording, participants listened only to the audio of the video and focused solely on the visuals within the video. Data collection was carried out with the informed voluntary consent of the participants.

The stimulus was described as a 7 min exposure. However, 30 non-overlapping 15 s epochs represent 450 s (7.5 min), exceeding that nominal duration. Acquisition timestamps that reconcile stimulus exposure with the retained recording duration are not reported. The relation between these durations therefore cannot be resolved from the available record. The reported epoch counts are retained without assuming that recording and stimulus durations were identical. The excerpt timestamps, presentation hardware and sound level were not logged.

The video was used to provide a common audiovisual condition during EEG acquisition. Attention, relaxation and meditative engagement were not assessed. Therefore, the experimental condition was treated as standardised audiovisual exposure rather than a verified meditation or mindfulness intervention.

EEG signals were recorded at a sampling frequency of 256 Hz with an EMOTIV Flex 32 wireless EEG system. The fixed channel order was Cz, Fz, Fp1, F7, F3, FC1, C3, FC5, FT9, T7, CP5, CP1, P3, P7, PO9, O1, Pz, Oz, O2, O10, P8, P4, CP2, CP6, T8, FT10, FC6, C4, FC2, F4, F8 and Fp2. Each 15 s epoch contained 3840 samples for each of the 32 channels.

Artefact control was performed during EEG acquisition. Recordings with visually apparent artefacts were reacquired. Signals that remained visibly contaminated after reacquisition were excluded. Independent component analysis, component rejection, regression-based correction, automated artefact removal, bad-channel interpolation and post hoc signal repair were not applied. Artefact management was therefore based on visual assessment, reacquisition and exclusion rather than computational signal correction.

Quantitative artefact scores, formal visual-rejection thresholds and participant-level rejection counts were not recorded. Information on reference and ground electrode locations, impedance criteria, online acquisition filters and notch-filter settings was also unavailable. These parameters are included among the recommended reporting elements for EEG studies [28,29].

The study was conducted in accordance with the Declaration of Helsinki and approved by the Non-Invasive Ethics Committee of Firat University (protocol code 2025/16-19, 6 November 2025). De-identified EEG recordings and group labels were used in the analysis. The data were not used for treatment, sentencing, parole or other individual clinical or legal decisions.

Information on medication use, sedative exposure, psychiatric diagnosis, substance use, sleep and vigilance state, handedness, education, head injury, psychometric measures and offence subtype was unavailable. These factors were considered unmeasured cohort characteristics because they may affect EEG activity. Table 1 describes the analysed cohort. Counts of screened, reacquired and excluded recordings are not fully reported, so a complete recruitment and exclusion flow cannot be reconstructed. Psychiatric status is not an unmeasured covariate here. It is part of the group definition and cannot be removed statistically in this cohort.

All 95 participants were adults aged 19–67 years. No age-matched, age-adjusted or age-stratified analysis was completed. Sex proportions were reported, but no matched analysis was performed. The effects of age and unmeasured clinical characteristics cannot be separated from recorded-offence status in this cohort.

### 2.2. RhythmFluxPat-XFE Pipeline

The computational workflow consisted of four main stages: RhythmFluxPat feature extraction, cumulative-weighted feature selection, tkNN classification and DRLobish-based interpretation. The complete workflow is illustrated in Figure 1.

Participant-labelled EEG recordings were first divided into non-overlapping 15 s epochs. RhythmFluxPat was then applied to each epoch to generate a reversible rhythm-transition feature vector. Four feature-ranking methods were evaluated within the cumulative-weighted selection framework. The selected feature subsets were assessed with the tkNN classifier, and the retained feature indices were subsequently decoded through DRLobish.

The control group was coded as class 1, and the recorded-offence group was coded as class 2. Participant identifiers were preserved throughout the analysis. During classifier-stage leave-one-subject-out evaluation, all epochs from one participant were assigned to the test fold, while the epochs from the remaining participants were used for classifier training.

The prediction and interpretation stages were kept separate. RhythmFluxPat features and the selected coordinates were used as classifier inputs. DRLobish was applied after feature selection and classification to convert the retained feature indices into readable source–rhythm–target routes.

#### 2.2.1. RhythmFluxPat

RhythmFluxPat generates one feature for every ordered source–target channel pair within each rhythm layer. Five layers represent within-rhythm temporal transitions, and four layers represent directed cross-rhythm transitions. Self-transitions are retained because the same channel may occupy a corresponding temporal rank at the current and future time points.

Each layer contains a 32 × 32 directed transition matrix, corresponding to 1024 ordered channel pairs. The nine layers therefore produce 9216 features for each EEG epoch. The rhythm layers, frequency ranges and corresponding feature-index intervals are presented in Table 2.

Table 2 fixes the layer order as A, B, G, T, D, AT, GA, TB and BG. The first five layers represent within-rhythm transitions; the last four represent ordered cross-rhythm relationships. Each feature index therefore maps deterministically to a rhythm layer, source channel and target channel.

Each EEG epoch contained 3840 samples per channel at a sampling frequency of 256 Hz. The epoch matrix was oriented as samples × channels and converted to double precision. Non-finite values were replaced with zero before feature extraction.

Robust global normalisation was applied to each epoch before frequency decomposition. The epoch median was subtracted from each value, and the result was divided by 1.4826 times the median absolute deviation. This transformation reduced the effect of isolated high-amplitude observations while preserving the relative temporal and spatial ordering of the EEG values.

The normalised EEG signals were decomposed into delta, theta, alpha, beta and gamma bands. Fourth-order Butterworth band-pass filters were applied in both forward and reverse directions. This zero-phase filtering procedure prevented the phase delay associated with one-directional filtering.

The Hilbert transform was applied to the filtered rhythm signals. Instantaneous envelopes were calculated for all five rhythms. Instantaneous phase values were also calculated for the rhythms used in cross-rhythm map construction. The five within-rhythm layers were generated directly from the corresponding rhythm envelopes.

The alpha-to-theta, gamma-to-alpha, theta-to-beta and beta-to-gamma layers were constructed from the source-rhythm envelope and the target-rhythm signal at a one-sample temporal lead. At 256 Hz, one sample corresponds to approximately 3.91 ms. The cross-rhythm maps combined envelope information, phase alignment and bounded directional contrast. Therefore, these maps represented temporally ordered rhythm relationships within the proposed algorithm.

For each rhythm map, the 32 channels were ranked from the largest to the smallest value at every time sample. A channel occupying a given rank at the current sample was treated as the source. The channel occupying the same rank at a future sample was treated as the target. Because every rank is linked at every sample, each channel appears once as a source and once as a target per sample and lag (Section 1.3).

Four temporal lags were used: 1, 2, 4 and 8 samples. These lags correspond to approximately 3.91, 7.81, 15.63 and 31.25 ms, respectively. The associated lag weights were 0.50, 0.25, 0.15 and 0.10. Thus, shorter temporal transitions received greater weight. These lags span 0.20–1.56% of a cycle at 0.5 Hz and 1.56–12.5% at 4 Hz. Delta-layer features therefore index rank persistence, not transitions within an oscillatory cycle.

The resulting weighted transitions formed a 32 × 32 directed matrix for each layer. Rows represented the current source channels, and columns represented the future target channels. A bounded value-dependent term was applied so that stronger paired observations contributed more to the transition matrix without dominating the transition count.

The observed transition matrix was subsequently compared with the expected transition pattern derived from the source and target marginal distributions. Transitions that could be largely explained by the marginal channel frequencies were down-weighted. Transitions that occurred more frequently than expected retained greater importance. The marginal-contrast coefficient was set to 0.12 for within-rhythm layers and 0.14 for cross-rhythm layers. Each resulting layer was then normalised to unit sum. Without value weights, the source and target marginals are uniform, so the expected matrix is constant (Appendix C). The contrast step then lowers every entry above α/1024 by the same amount and shrinks smaller entries in proportion. It cannot single out or suppress self-transitions.

The complete RhythmFluxPat feature-extraction procedure is summarised in Algorithm 1.
**Algorithm 1.** RhythmFluxPat feature construction.**Input:** one 15 s EEG epoch with 32 channels. **Output:** one 9216-element directed rhythm-route vector.**Step****Operation**1Orient the epoch as samples x channels, convert to double precision, replace non-finite values with zero, and verify 3840 samples per channel at 256 Hz.2Apply robust global z normalization using the epoch median and 1.4826 x median absolute deviation.3Apply fourth-order zero-phase Butterworth band-pass filters for delta, theta, alpha, beta and gamma (30–80 Hz).4Calculate Hilbert envelopes for all five bands and phases for theta, alpha, beta and gamma.5Use envelopes as the five within-rhythm maps; construct AT, GA, TB and BG from one-sample-led target envelopes/phases, phase alignment and bounded directional contrast.6Rank the 32 channels at each sample from largest to smallest map value. Row-wise robust standardisation does not change this order; its values are used only by the bounded weight in Step 8.7At lags 1, 2, 4 and 8, link the channel at each current rank to the channel at the same future rank; use lag weights 0.50, 0.25, 0.15 and 0.10.8Modulate each source-target event with a bounded value-dependent weight so stronger paired observations contribute slightly more without dominating the transition count. The weight uses the row-standardised map values.9Apply marginal contrast with alpha = 0.12 for within-rhythm layers and 0.14 for cross-rhythm layers, then normalize each layer to unit sum.10Vectorize each layer with source channel varying fastest, followed by target channel and layer, so every feature index can be decoded deterministically.11Concatenate layers A, B, G, T, D, AT, GA, TB and BG.

As presented in Algorithm 1, RhythmFluxPat follows a deterministic extraction procedure. The layer order and vectorisation rule remain fixed for every EEG epoch. Consequently, the numerical position of each feature is preserved across participants and can be decoded without an additional feature-mapping model.

The source-to-target direction represents temporal order within the rank-transition procedure. It does not represent anatomical connectivity, effective connectivity, information transfer or causal neural influence. RhythmFluxPat should therefore be interpreted as a rhythm-resolved temporal channel-transition descriptor. Ranking is invariant to any positive row-wise location–scale transformation, so the robust standardisation does not change which channel occupies which rank (Appendix C). It is kept only because the Step-8 weight uses standardised values.

#### 2.2.2. CWImRMR Feature Selection

RhythmFluxPat generated 9216 features for each EEG epoch. A feature-selection stage was therefore applied to reduce dimensionality and retain a compact set of class-relevant coordinates. Four supervised feature-ranking methods were evaluated: neighbourhood component analysis (NCA), chi-square, minimum redundancy maximum relevance (mRMR) and ReliefF.

NCA evaluates features according to their contribution to local class separation. Chi-square measures the dependence between individual features and class labels. mRMR prioritises features with high class relevance and limited mutual redundancy. ReliefF estimates feature importance through local differences between observations from the same and opposite classes. The four methods were evaluated under the same cumulative-weighted selection procedure.

Before feature ranking, cohort-level min–max normalisation was applied to the complete 2554 × 9216 feature matrix. Ranking scores were shifted to non-negative values and normalised by total score mass; a cumulative score curve was then calculated. As this preprocessing and the supervised feature selection were performed before the outer LOSO folds, information from held-out participants could influence the fixed 352-feature panel. Training-only z-scoring during classifier fitting does not remove this feature-selection limitation.

The candidate search started with the first feature prefix that reached 5% of the cumulative score mass. The prefix size was subsequently increased one feature at a time up to a maximum of 500 features. Each candidate prefix was evaluated with the complete 90-candidate tkNN search. Thus, the selected feature count was not fixed before classification.

The feature-ranking method and prefix size that produced the highest classifier-stage leave-one-subject-out accuracy were retained. The best-performing branch was obtained with mRMR and contained 352 features. This cumulative-weighted mRMR configuration was termed CWImRMR. This choice used all participants, so the selected maximum is optimistic (Appendix B).

Comparison of feature-selection methods. The four cumulative-weighted branches were evaluated using the same analysis procedure. The retained panels contained 305 NCA, 142 chi-square, 352 mRMR and 202 ReliefF coordinates. The mRMR method selected the 352-coordinate feature panel used for interpretation. The selected panel comprised 176 coordinates assigned to each class, with leading routes T8 → T8 (AUC 0.793; \|g\| = 1.064) and C4 → CP6 (AUC 0.770; \|g\| = 1.024). Six pairwise intersection panels were also formed, with 1 to 44 coordinates. Each of the ten panels was classified with the same participant-held-out tkNN routine. The ranked branch outputs were then combined by iterative majority voting (IMV) of the 3 to 10 best branches. All panels were selected with full-cohort labels. These results are therefore development-stage estimates (Section 4.2).

The complete CWImRMR selection procedure is summarised in Algorithm 2.
**Algorithm 2.** CWImRMR selection in the development analysis.**Input:** 2554 × 9216 feature matrix, class labels and participant identifiers. **Output:** selected CWImRMR development panel.**Step****Operation**1Apply cohort-level min-max normalization to the complete matrix.2Rank all features with MATLAB mRMR.3Shift scores to non-negative values and normalize their total mass.4Start at the first prefix reaching 5% cumulative score mass.5Evaluate every prefix from that point to 500 features with the 90-candidate tkNN classifier-stage LOSO routine.6Select the prefix with the highest observed epoch accuracy.7Retain the first 352 ranked features for the winning mRMR branch.8Treat the resulting maximum as an exploratory selected estimate because the same LOSO stream was used for selection and reporting.

As presented in Algorithm 2, CWImRMR combines feature ranking with a cumulative-prefix search. The procedure does not assume that a predefined number of features is optimal. Instead, candidate feature counts are evaluated according to the distribution of ranking-score mass and the observed classifier-stage performance.

The final CWImRMR panel retained 352 of the original 9216 RhythmFluxPat features (3.82%). Each selected index remained traceable to its rhythm layer, source channel and target channel during DRLobish interpretation. The fixed panel had been selected using all participant labels before classifier-nested validation.

The class assignment of the selected coordinates was performed after feature selection. Participant-balanced class means were calculated for each retained feature, and the feature was assigned to the class with the larger mean value. This post-selection procedure produced 176 control-assigned and 176 recorded-offence-assigned coordinates. The equal class distribution was not imposed during feature ranking, feature-prefix selection or classifier training.

The feature count and tkNN configuration were selected from the same classifier-stage LOSO prediction stream used to report the observed performance. Therefore, the selected result represents a development-stage estimate rather than a fully nested confirmatory estimate. 

The 352-feature development panel yielded 94.01% selected classifier-stage epoch accuracy. With that panel fixed, classifier-nested participant-level LOSO yielded 93.66%. This fixed-panel value is optimistic because the panel was selected with all participants (Appendix B).

#### 2.2.3. tkNN Classification

The selected RhythmFluxPat feature subsets were classified with the t algorithm-based k-nearest-neighbour framework, termed tkNN [19]. tkNN extends the conventional k-nearest-neighbour classifier through a systematic search over neighbour count, distance metric and distance-weighting rule. 

The classifier-selection procedure was implemented as follows. The search space contained 90 candidate configurations. The number of neighbours was varied from 1 to 10. Three distance metrics were evaluated: city-block, Euclidean and cosine distance. Three distance-weighting rules were also evaluated: equal, inverse-distance and squared-inverse-distance weighting. Each neighbour count was combined with every distance metric and weighting rule. Classifier performance was evaluated through leave-one-subject-out cross-validation. In each fold, all epochs from one participant were assigned to the test set. The epochs from the remaining 94 participants were used for classifier training. This procedure prevented epochs from the same participant from appearing simultaneously in the classifier training and test sets. Predictor standardisation was fitted independently within each classifier training fold. The estimated standardisation parameters were then applied to the epochs of the held-out participant. Thus, the held-out participant did not contribute to the mean and scale parameters used during classifier fitting. Each tkNN candidate was evaluated across all 95 participant-held-out folds. The predictions obtained for the held-out epochs were concatenated after all participants had served as the test participant. Epoch-level performance was then calculated from the complete concatenated prediction stream. The tkNN candidate with the highest observed epoch-level accuracy was retained as the selected classifier-stage configuration. The complete classification procedure is summarised in Algorithm 3.
**Algorithm 3.** Classifier-stage tkNN LOSO selection procedure.**Input:** selected feature matrix, labels and 95 participant identifiers. **Output:** selected classifier-stage LOSO prediction stream.**Step****Operation**1Create 90 candidates from k = 1–10, three distances and three distance-weight rules.2Hold out one participant and place all of that participant’s epochs in the test fold.3Fit predictor standardization and the current kNN candidate using the remaining participants only.4Predict all epochs from the held-out participant.5Repeat until every participant has served as the test participant.6Concatenate held-out predictions and calculate epoch performance for that candidate.7Repeat the LOSO cycle for all 90 candidates and retain the highest-accuracy stream.8This describes the initial classifier-selection procedure. The completed classifier-nested outer LOSO evaluation is described in Section 2.2.5.

Algorithm 3 describes the initial classifier-selection procedure. Participants were kept separate during classifier fitting and prediction. The completed classifier-nested evaluation is described separately in Section 2.2.5.

Epoch-level predictions were also grouped according to participant identity. A participant-level decision was obtained through majority voting across all available epochs from that participant. Therefore, the epoch-level analysis measured prediction consistency across individual EEG windows, whereas the participant-level analysis summarised the dominant prediction for each participant.

The classifier-selection records contain the tkNN search space and final prediction stream but not the winning neighbour count, distance metric or weighting rule. The MATLAB (2023b) release was also not recorded. These missing settings cannot be recovered from aggregate accuracy.

**Figure A1:**
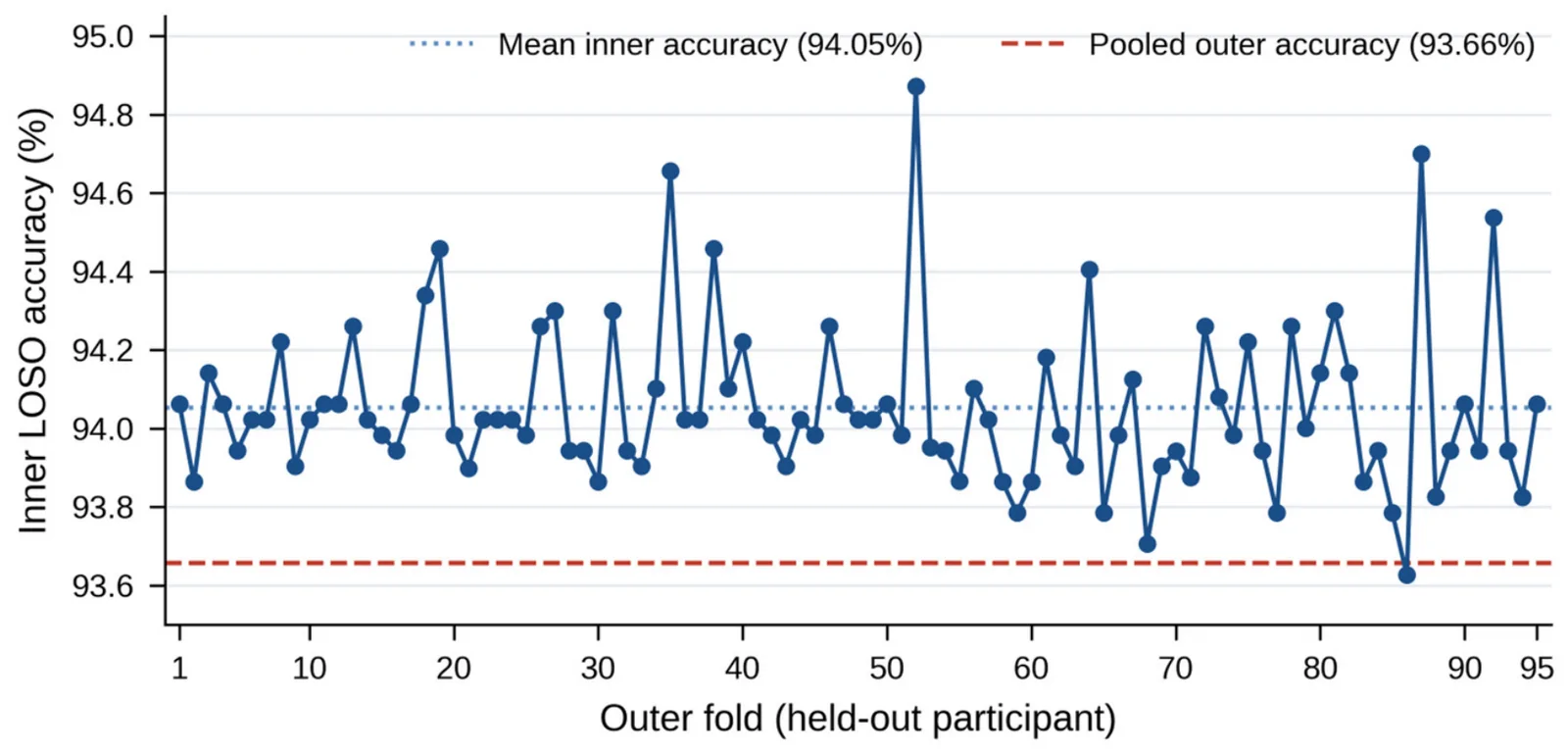
Reported inner accuracy for each of 95 outer folds in the fixed-panel classifier-nested analysis. The dashed line marks pooled outer accuracy (93.66%); the dotted line marks mean inner accuracy (94.05%). Their difference does not quantify feature-selection bias.

**Figure A2:**
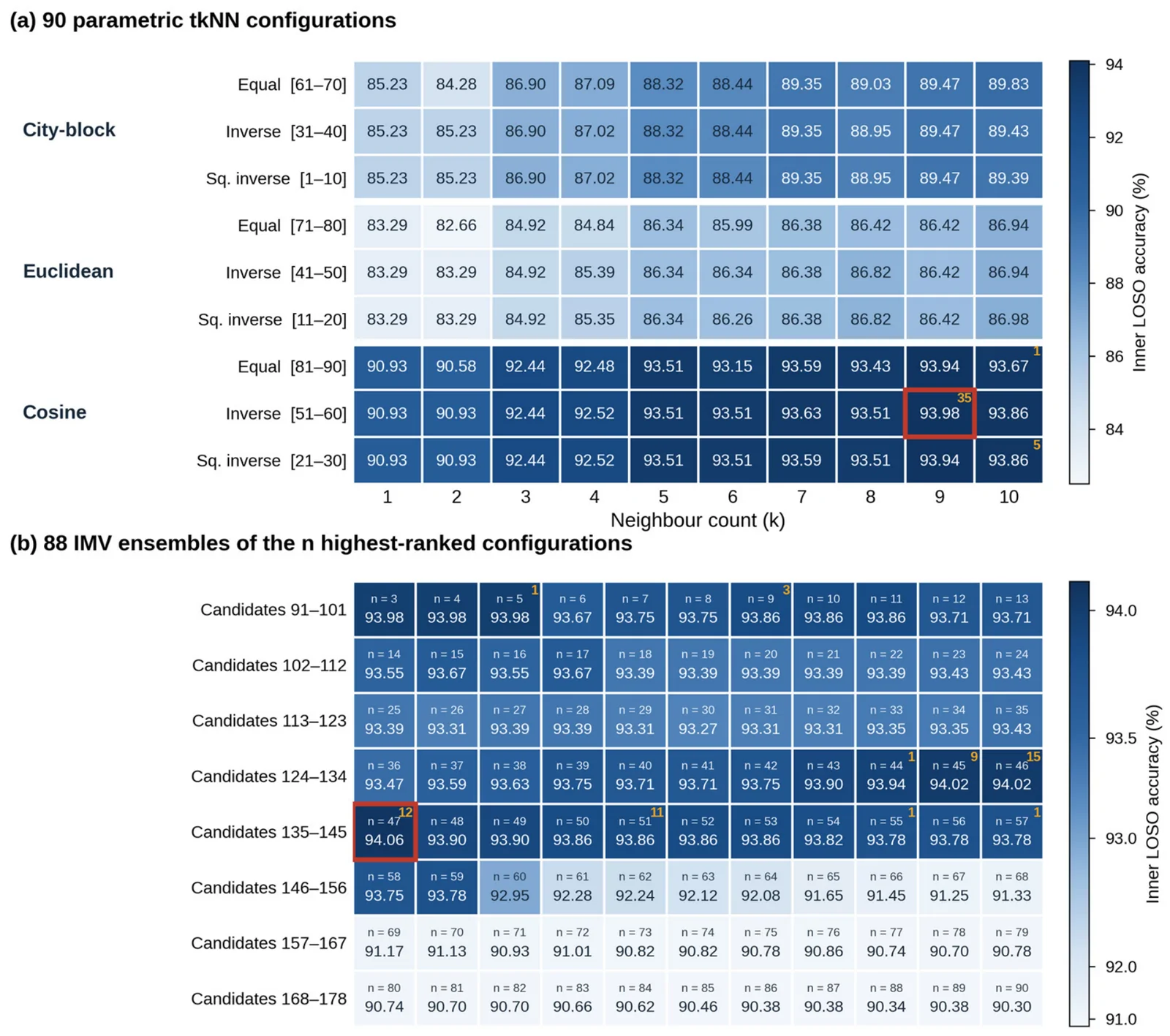
Inner LOSO accuracy (%) of the 178 classifier candidates in the outer fold that held out participant 95 (94 development participants; 2526 epochs). (**a**) The 90 tkNN configurations by distance metric, weighting rule and neighbour count; candidate indices are given in brackets. (**b**) The 88 IMV ensembles, where n is the number of pooled configurations. Red outlines mark the best candidate in each panel, and amber numbers show how often a candidate was selected across the 95 outer folds. Panel (**b**) uses a non-linear colour scale to separate values near the top of the range. Complete candidate results were not stored for the other 94 outer folds. All values are inner-loop estimates, not outer-test results.

**Figure A3:**
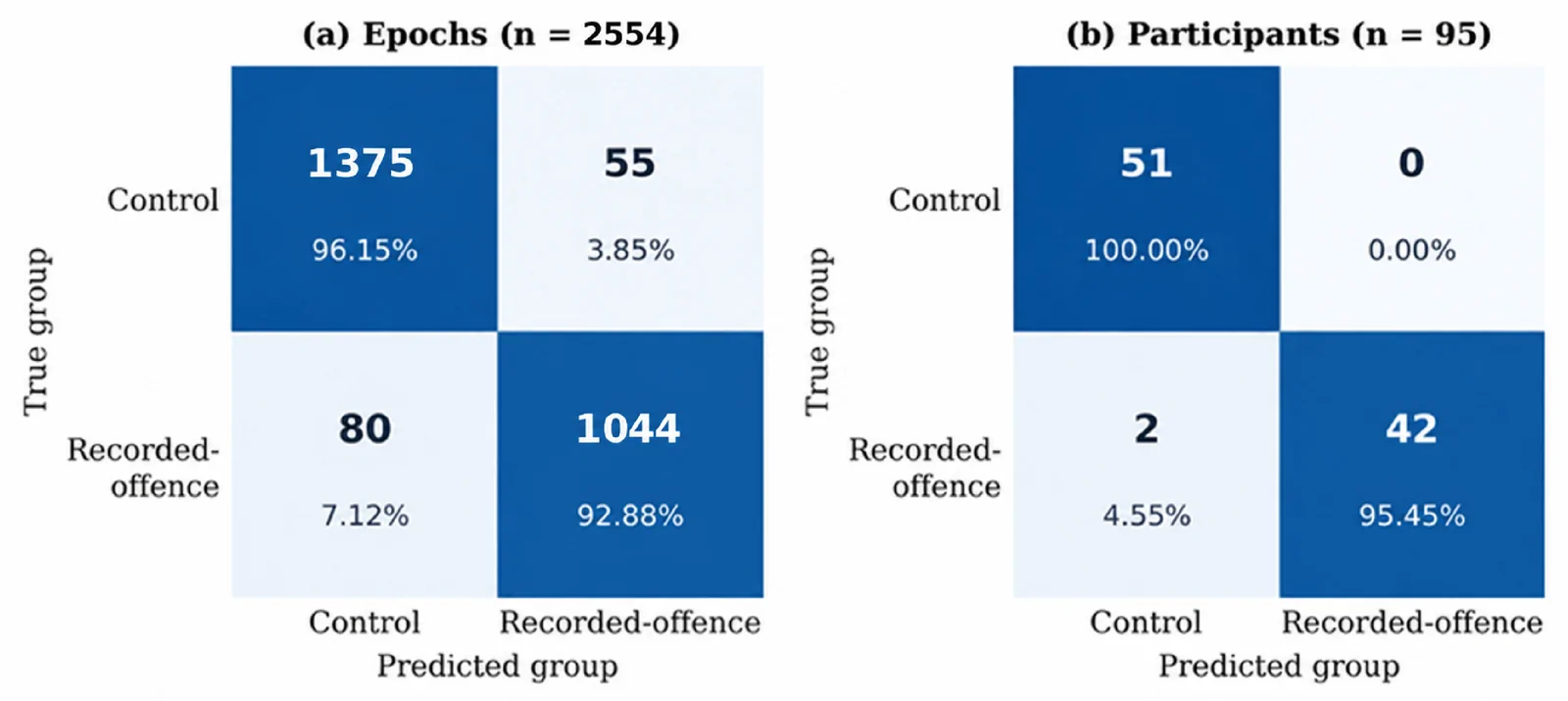
Confusion matrices of the nested SVM with the fixed 352-feature panel. (**a**) Epochs (n = 2554). (**b**) Participants (n = 95). Rows show observed groups and columns predicted groups; each cell gives the count and the row percentage. The panel was selected with full-cohort labels.

The classifier-nested search evaluated the same 90 tkNN configurations and 88 iterative majority-voting (IMV) ensembles (Section 2.2.5). The selected candidate index was recorded for every outer fold. Appendix A reports the inner accuracy for each outer fold and summarises the selected indices, which follow the enumeration order of the classifier-nested LOSO script. Figure A1 presents the selected candidate indices across the outer folds. Figure A2 maps each index to its distance metric, weighting rule and neighbour count or ensemble size. Figure A3 summarises the relationship between these selected indices and the pooled outer accuracy.

The selected-model result was the highest-accuracy candidate from the development-stage LOSO search. Feature ranking, prefix selection and classifier selection used the same reporting stream. This initial selected-model analysis result is distinct from the completed classifier-nested analysis in Section 2.2.5.

#### 2.2.4. DRLobish Interpretation

DRLobish was applied after feature selection and classification to convert the retained RhythmFluxPat coordinates into readable EEG routes. The interpretation stage did not generate additional classifier inputs and did not modify the stored tkNN predictions.

Each of the 352 selected feature indices was decoded into three components: rhythm layer, source channel and target channel. The fixed RhythmFluxPat vectorisation order allowed this decoding to be performed deterministically. Therefore, every selected numerical feature could be traced back to its original EEG coordinate.

For regional reporting, the 32 EEG channels were mapped to 14 predefined regions: left frontal (FL), right frontal (FR), left temporal (TL), right temporal (TR), left parietal (PL), right parietal (PR), left occipital (OL), right occipital (OR), left central (CL), right central (CR), Cz, Fz, Pz and Oz. The original channel-level coordinates were retained alongside the regional representations.

The canonical DRLobish structure contains 1280 source–rhythm–target symbols derived from 16 regions and five rhythm classes [20]. The present EEG montage did not contain the AL and AR regions. Therefore, the regional reporting structure was adapted to the 14 available regions. The A, B, G, T and D labels followed the conventional DRLobish rhythm grammar. The AT, GA, TB and BG labels represented the ordered cross-rhythm layers defined by RhythmFluxPat.

To prevent participants with more epochs from having greater influence on the interpretation results, feature values were first averaged within each participant. Class-level summaries were then calculated from these participant-level means.

Each selected route was characterised through three complementary measures:

1. Participant-balanced area under the receiver operating characteristic curve;
2. Absolute Hedges’ g effect size;
3. Feature-rank priority within the CWImRMR panel.

Participant-balanced AUC described the degree of class separation without depending on the direction of the difference [30]. Absolute Hedges’ g quantified the standardised magnitude of the difference between the participant-level class means [31]. Feature-rank priority assigned greater importance to features retained earlier in the CWImRMR ranking.

The DRLobish atomic evidence score combined these three measures. AUC contributed 50% of the score, absolute Hedges’ g contributed 35% and feature-rank priority contributed 15%. AUC was rescaled from chance-level separation to perfect separation. The effect-size contribution was bounded to prevent a single extreme value from dominating the evidence score.

Each selected feature was assigned to the class with the larger participant-balanced mean. Class-specific route strength was then calculated by combining the normalised class mean and atomic evidence score. The normalised class mean contributed 55%, and the atomic evidence score contributed 45%. Several channel-level coordinates could map to the same regional route. In these cases, the regional aggregation score was calculated as 70% of the maximum atomic evidence and 30% of the mean atomic evidence. This rule emphasised the strongest channel-level coordinate while retaining information from the remaining coordinates mapped to the same regional route.

Each decoded route was written in the form:

**Source region—Rhythm transition → Target region**

The label ‘Offence’ denotes the recorded-offence group throughout the graphical results. DRLobish uses four grammar categories: loop, edge, corner and Symbolic Route Type IV. Type IV is an algorithmic reporting category and does not measure anatomical disconnection, axonal damage, disrupted functional connectivity or causal pathways.

The complete DRLobish interpretation procedure is summarised in Algorithm 4.
**Algorithm 4.** DRLobish selected-feature reporting workflow.**Input:** The selected indices, selected feature values, labels and participant identifiers. **Output:** Descriptive route cards, tables and surrogate tree.**Step****Operation**1Decode each selected index into layer, source channel and target channel.2Map channels to 14 reporting regions while retaining the original channel identities.3Write Source—Rhythm → Target and attach the code-defined loop, edge, corner or Type IV label.4Average epoch values within participant before class-level evidence calculation.5Calculate separability AUC, absolute Hedges’ g and rank prior.6Calculate atomic evidence with fixed weights 0.50, 0.35 and 0.15.7Assign the feature to the class with the larger participant-balanced mean.8Calculate class-specific route strength with fixed 0.55 mean and 0.45 evidence weights.9Aggregate repeated regional routes as 0.70 maximum plus 0.30 mean atomic evidence.10Report participant-balanced AUC, effect size and route score together; fit the three-feature surrogate only as an explanation audit.

As shown in Algorithm 4, the interpretation was based on participant-balanced summaries rather than raw epoch counts. This procedure prevented participants with a larger number of usable epochs from dominating the class-level route scores. 

AUC, Hedges’ g and feature-rank priority were interpreted together. AUC described class separation, Hedges’ g described effect magnitude and feature rank reflected the position of the coordinate in the selected panel. No single measure was treated as an independent diagnostic threshold. 

The DRLobish outputs represent reversible sensor-space descriptions of the selected feature panel. They do not represent anatomical tracts, source-localised cortical activation, effective connectivity or causal neural pathways. The resulting route scores were used for descriptive interpretation of the classifier input space.

DRLobish provides feature traceability by decoding each selected input into its rhythm, source sensor and target sensor. It does not explain an individual tkNN prediction. Atomic evidence uses fixed weights of 0.50 for participant-balanced AUC, 0.35 for absolute Hedges’ g and 0.15 for ranking priority. Class-specific route strength uses 0.55/0.45 for the normalised class mean and atomic evidence; regional pooling uses 0.70/0.30 for maximum and mean atomic evidence. These descriptive weights were not validated against physiological criteria. They act after classification and can change route ordering but cannot change predictions.

#### 2.2.5. Classifier-Nested Participant-Level Validation

**Figure 2:**
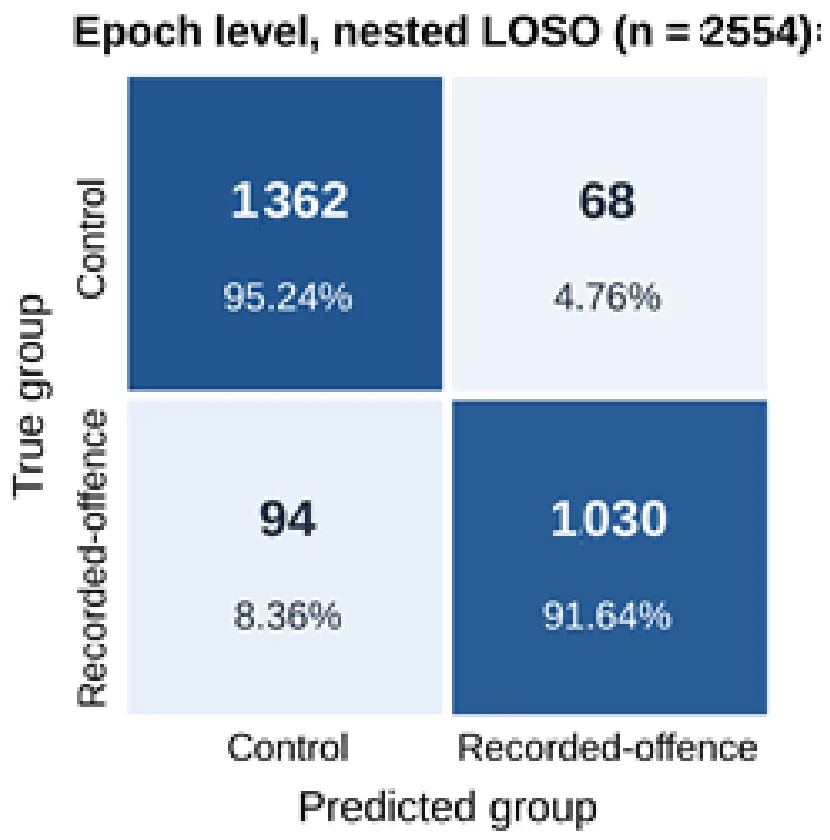
Epoch-level confusion matrix for classifier-nested participant-level LOSO with the fixed 352-feature panel (n = 2554). Rows show observed groups and columns show predicted groups; each cell gives the count and row percentage. Feature selection was performed before outer splitting. Group labels describe recorded history, not current violence or future risk.

**Figure 3:**
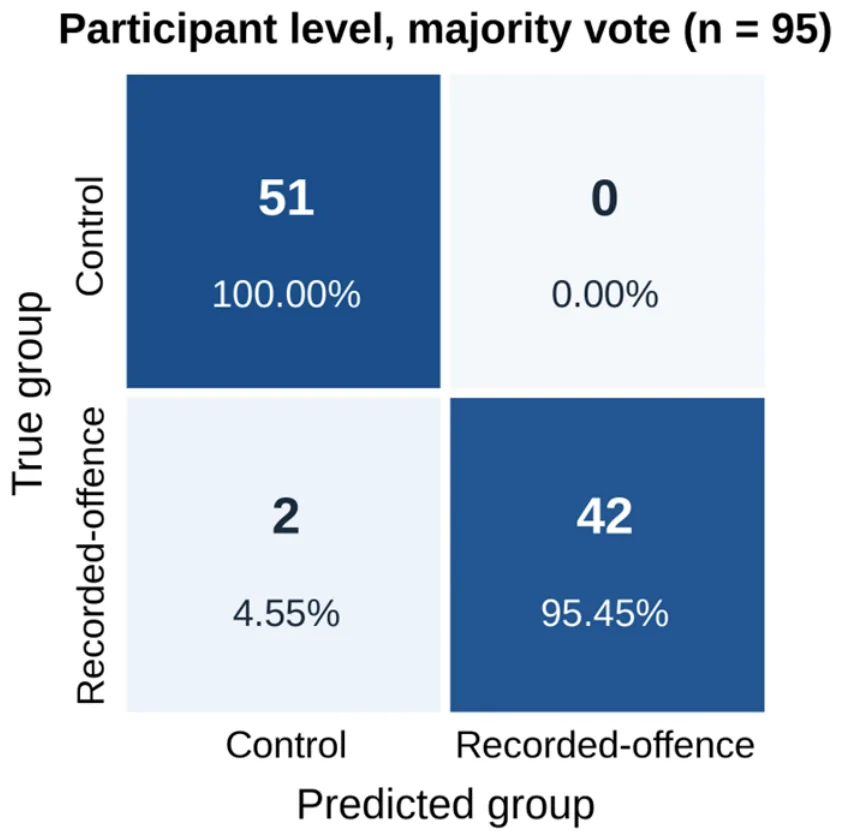
Participant-level majority-vote confusion matrix for classifier-nested participant-level LOSO with the fixed 352-feature panel (n = 95). Each participant contributes one aggregated decision; each cell gives the count and row percentage. Votes use the same held-out epoch predictions as Figure 2 and do not form a second independent test.

Classifier-nested participant-level LOSO used 95 outer folds. One participant was held out for testing, and the remaining 94 formed the development set. Inner LOSO over those 94 participants evaluated 90 tkNN configurations and 88 IMV ensembles formed from the 3 to 90 highest-ranked configurations. Candidates were ranked by pooled inner epoch accuracy. The selected classifier or ensemble was refitted on all 94 development participants and applied once to the outer test participant. Predictor z-scoring was fitted within each inner training fold and, for final refitting, on the outer training set only. The 352 CWImRMR features remained fixed across outer folds because they had been selected using the full cohort. Thus, classifier selection was nested, but feature ranking and prefix selection were not. Outer-held-out predictions formed the epoch confusion matrix; majority voting formed the participant matrix (Figure 2 and Figure 3). Table A1 reports inner accuracies, and Appendix A summarises the selected candidate indices. The saved label, prediction, and participant-index vectors were checked independently, and both confusion matrices and the participant votes were reproduced (Appendix D). This check does not show that feature selection was nested.

Code for a matched band-power comparison is provided in Supplementary Code S1. It has not yet been applied to the original epochs (Section 4.2 and Section 4.4).

#### 2.2.6. Leakage Simulation

A simulation measured how much accuracy cohort-wide selection can produce without any signal. The simulated data copied the cohort structure: 51 and 44 participants, 1430 and 1124 epochs (7–30 per participant) and 9216 features. Each feature had a participant random effect with an intraclass correlation of 0.5, and the labels carried no information. Three schemes were applied to the same data: the initial classifier-selection procedure, the fixed-panel classifier-nested scheme used in this study and a fully nested scheme. In the fully nested scheme, mRMR ranking, prefix search and tkNN/IMV choice were refitted inside each outer fold on the training participants only. For speed, mRMR pre-filtered 1000 features by relevance, and prefixes up to 200 features were searched in steps of 10. Three noise seeds were run. A fourth data set added a 0.5-SD group shift to 50 features to check that the fully nested scheme still recovers a real signal. Its results are given in Appendix B.

#### 2.2.7. Nested Support-Vector Machine

A support-vector machine (SVM) was added to test whether the result depends on the tkNN search. It used the same participant-held-out outer folds. Within each outer training set, predictors were z-scored. The kernel and its hyperparameters were then chosen by participant-grouped five-fold cross-validation (linear: C ∈ {0.001, 0.01, 0.1, 1}; radial basis function: C ∈ {0.1, 1, 10, 100}, γ ∈ {0.25, 1, 4}/p, where p is the number of features). The selected model was refitted on all training participants and applied once to the held-out participant. Participant decisions used majority voting. Epoch-level 95% intervals used a participant-cluster bootstrap (2000 resamples), and participant-level intervals used the Wilson method. As in the tkNN analysis, the feature panel was fixed before outer splitting. This analysis therefore nests classifier choice but not feature selection.

## 3. Results

### 3.1. Evaluation Settings

The final development matrix contained 2554 EEG epochs, 9216 initial RhythmFluxPat features and 95 participant identifiers. The control group was treated as the negative class, and the recorded-offence group was treated as the positive class.

Classifier performance was evaluated through participant-held-out leave-one-subject-out cross-validation. In each fold, all epochs from one participant were assigned to the test set. The epochs from the remaining 94 participants were used for classifier fitting. This procedure was repeated until every participant had served as the held-out test participant.

The held-out epoch predictions from all 95 folds were concatenated to calculate epoch-level performance. Thus, each epoch-level prediction was generated in a classifier fold that excluded all epochs from the corresponding participant.

Participant-level decisions were obtained through majority voting across the available epoch predictions of each participant. The class predicted for more than half of the participants’ epochs was assigned as the final participant-level decision. No tied majority vote occurred among the 95 participants. Accuracy, recorded-offence sensitivity, control specificity and balanced accuracy were calculated from the corresponding confusion matrices. Recorded-offence sensitivity represented the proportion of participants or epochs from the recorded-offence group that were classified correctly. Control specificity represented the proportion of controls that were classified correctly. Balanced accuracy was calculated as the mean of recorded-offence sensitivity and control specificity. Positive predictive value, negative predictive value, F1 score and Matthews correlation coefficient were also calculated for the epoch-level analysis. The recorded-offence group was used as the positive class for these measures.

Wilson 95% confidence intervals were calculated for participant-level accuracy, sensitivity and specificity. Each participant contributed one majority-vote outcome. These intervals describe uncertainty in the observed proportions; they do not account for the fixed-panel selection bias or dependence between overlapping cross-validation training sets.

The initial classifier-selection procedure yielded 94.01% epoch accuracy for panel and classifier selection. The classifier-nested analysis is the reference analysis of the fixed panel. Each participant contributes one majority-vote decision; this aggregation does not remove upstream feature-selection bias. Both values depend on a panel selected with all participants and are optimistic.

### 3.2. Classification Results

All accuracies in this section use the panel selected on the full cohort, so they are optimistic. The values below therefore describe the selected panel within this cohort. They do not estimate performance on new participants.

In the classifier-nested LOSO evaluation, RhythmFluxPat–CWImRMR–tkNN correctly classified 2392 of 2554 EEG epochs (93.66%). The initial classifier-selection procedure correctly classified 2401 epochs (94.01%). The aggregate difference was nine correct epochs, or 0.35 percentage points. This comparison does not imply that only nine individual predictions differed.

Under classifier-nested LOSO, 1362 of 1430 control epochs were classified correctly and 68 were assigned to the recorded-offence group; 1030 of 1124 recorded-offence epochs were classified correctly and 94 were assigned to the control group. Figure 2 shows the nested epoch-level confusion matrix. In the selected-model evaluation, 1361 control and 1040 recorded-offence epochs were classified correctly, with 69 and 84 errors, respectively.

As shown in Figure 2, the classifier-nested LOSO analysis achieved 91.64% recorded-offence sensitivity, 95.24% control specificity and 93.44% balanced accuracy. The nested epoch-level accuracy (93.66%) was close to, but distinct from, the original selected-stage estimate (94.01%).

For the classifier-nested epoch predictions, positive predictive value was 93.81%, negative predictive value 93.54%, F1 score 92.71% and Matthews correlation coefficient 0.871, with the recorded-offence group as the positive class. Epochs are clustered within participants. Naive binomial intervals over 2554 epochs were therefore not reported; participant-resampled epoch intervals remain unavailable.

The 95 recorded inner-fold accuracies in Table A1 had a mean of 94.05% (SD 0.21%; median 94.02%; range 93.63–94.87%). The pooled outer epoch accuracy was 93.66%. Their numerical gap (0.40 percentage points) does not estimate or rule out feature-selection optimism.

Epoch-level predictions were subsequently aggregated through majority voting for each participant. The final participant-level confusion matrix is presented in Figure 3.

Majority voting on the classifier-nested LOSO predictions classified 93 of 95 participants correctly (97.89%; Wilson 95% confidence interval 92.65–99.42%). This is one participant fewer than the initial classifier-selection count of 94, and the epoch confusion matrices also differ. It remains conditional on the cohort-selected feature panel.

Majority voting classified all 51 control participants and 42 of the 44 recorded-offence participants correctly; both errors were offence-group participants assigned to the control group. These two participants contributed 8 and 28 epochs, of which 3 and 12, respectively, were assigned to the recorded-offence group. No second sample was involved. Each decision was formed from that person’s own held-out epoch predictions.

The participant-level recorded-offence sensitivity was 95.45%, and the control specificity was 100.00%. The resulting balanced accuracy was 97.73%. No participant produced a tied majority vote, and no abstention rule was required. The corresponding Wilson 95% confidence intervals were 84.87–98.74% for sensitivity and 93.00–100.00% for specificity.

Table 3 summarises the classifier-nested epoch-level and participant-level performance results.

As shown in Table 3, majority voting yielded higher numerical accuracy than individual-epoch classification. This reflects aggregation of correlated EEG epochs for a given participant and is not evidence from an additional independent cohort.

The participant-level majority-vote result is an aggregation of the nested held-out epoch predictions, not an independent classification experiment. The nested epoch and participant results each have a different denominator and are not interchangeable estimates.

The nested SVM (Section 2.2.7) classified 2419/2554 epochs correctly (94.71%; participant-cluster bootstrap 95% CI 92.30–96.88%). Recorded-offence sensitivity was 92.88%, control specificity 96.15% and balanced accuracy 94.52%. It classified 93/95 participants correctly (97.89%; Wilson 95% CI 92.65–99.42%; Figure A3). A linear kernel was chosen in 81 of 95 outer folds, with C = 0.001 in 76 folds. The SVM and tkNN errors were not identical: the 8-epoch participant missed by tkNN was classified correctly by the SVM. Two classifier families thus gave similar results. The result therefore does not depend on the tkNN search. It still carries the optimism of cohort-wide feature selection. Supplementary Table S1 gives the fold-wise SVM record for all 95 participants.

### 3.3. XAI Results

All explanation results were generated from the 352 features retained by CWImRMR. Each feature was decoded into its rhythm layer, source channel and target channel. Participant-balanced class means were then used to assign each coordinate to the class with the larger mean value. The resulting feature panel contained 176 control-assigned coordinates and 176 recorded-offence-assigned coordinates. This equal distribution emerged from the participant-balanced class means and was not imposed during feature selection or classifier training. All XAI results describe the full-cohort development panel. They are descriptive and are not used for performance claims.

#### 3.3.1. Route-Level DRLobish Results

Within the control-assigned feature set, gamma-to-alpha accounted for 19.91% of the evidence distribution. Alpha-to-theta accounted for 18.12%, and within-rhythm gamma accounted for 15.56%. Edge-labelled routes represented 33.68% of the control-assigned structural grammar.

**Figure 4:**
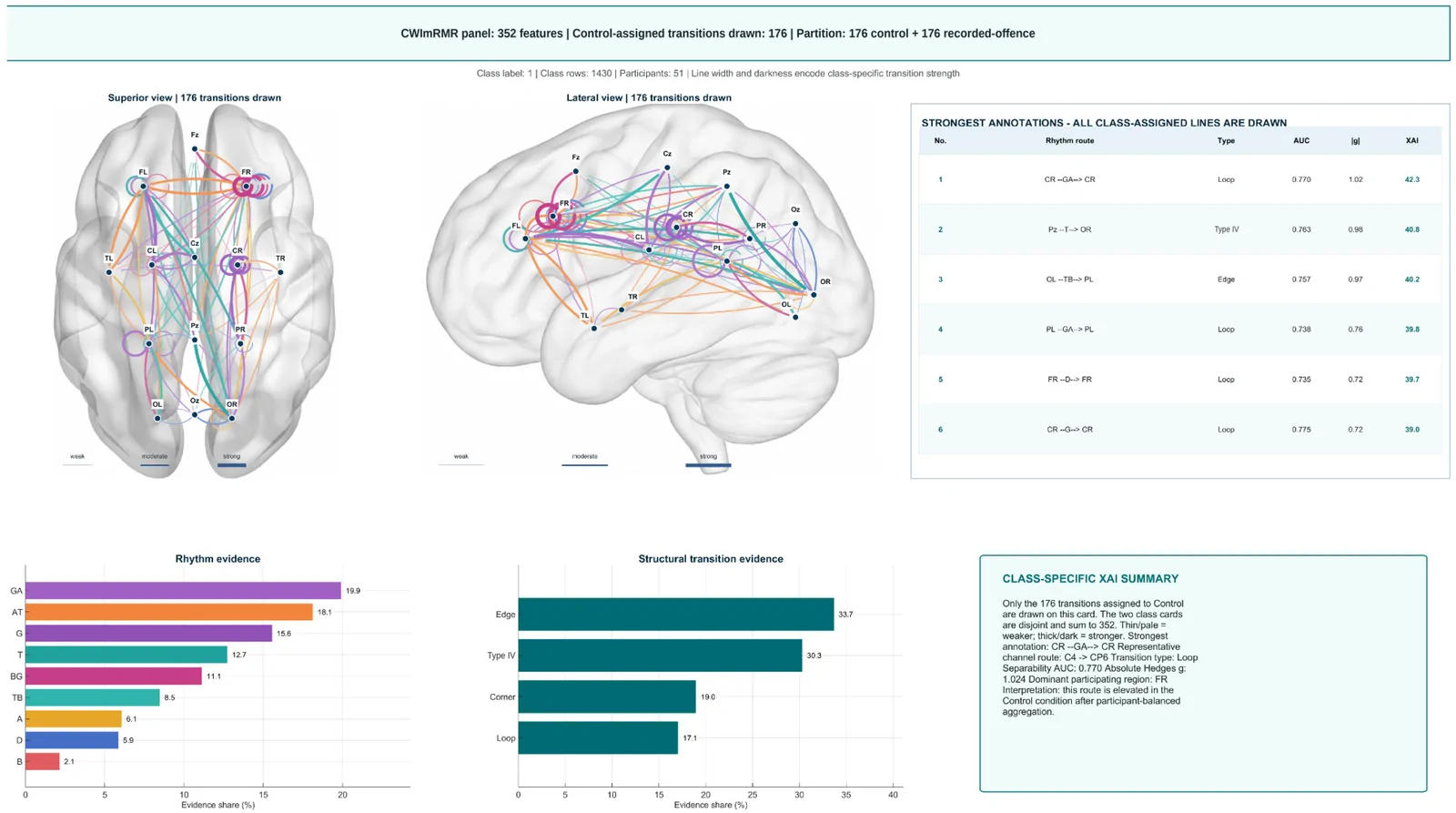
Control-assigned DRLobish route card. All 176 selected control-assigned transitions are displayed. Line properties encode descriptive route strength; no route is presented as a validated biomarker.

The leading aggregated control route was a right-central gamma-to-alpha loop. At channel level, this route was represented by C4 → CP6. It achieved a participant-balanced AUC of 0.770, an absolute Hedges’ g of 1.024 and an XAI score of 42.3. The complete control-assigned DRLobish route card is presented in Figure 4.

As shown in Figure 4, the control-assigned evidence was distributed across within-rhythm and cross-rhythm routes. Gamma-to-alpha and alpha-to-theta transitions represented the largest rhythm-level contributions. The displayed line properties represent descriptive route strength and do not define diagnostic thresholds.

The recorded-offence-assigned feature set also contained 176 coordinates. Type IV-labelled routes accounted for 60.11% of its structural grammar, and edge, corner and loop labels accounted for 17.57%, 12.64% and 9.68%, respectively. Type IV is a code-defined DRLobish category and does not describe anatomical or functional disconnection.

Delta accounted for 16.33% of the recorded-offence-assigned evidence distribution, and beta-to-gamma accounted for 16.59%. The highest-scoring recorded-offence-assigned coordinate was a right-temporal alpha-to-theta loop represented by T8 → T8. This coordinate achieved a participant-balanced AUC of 0.793, an absolute Hedges’ g of 1.064 and an XAI score of 59.2. This top coordinate is a self-transition at a peripheral temporal sensor. It indexes how persistently T8 kept its alpha-to-theta rank, and it may be sensitive to temporal muscle activity.

**Figure 5:**
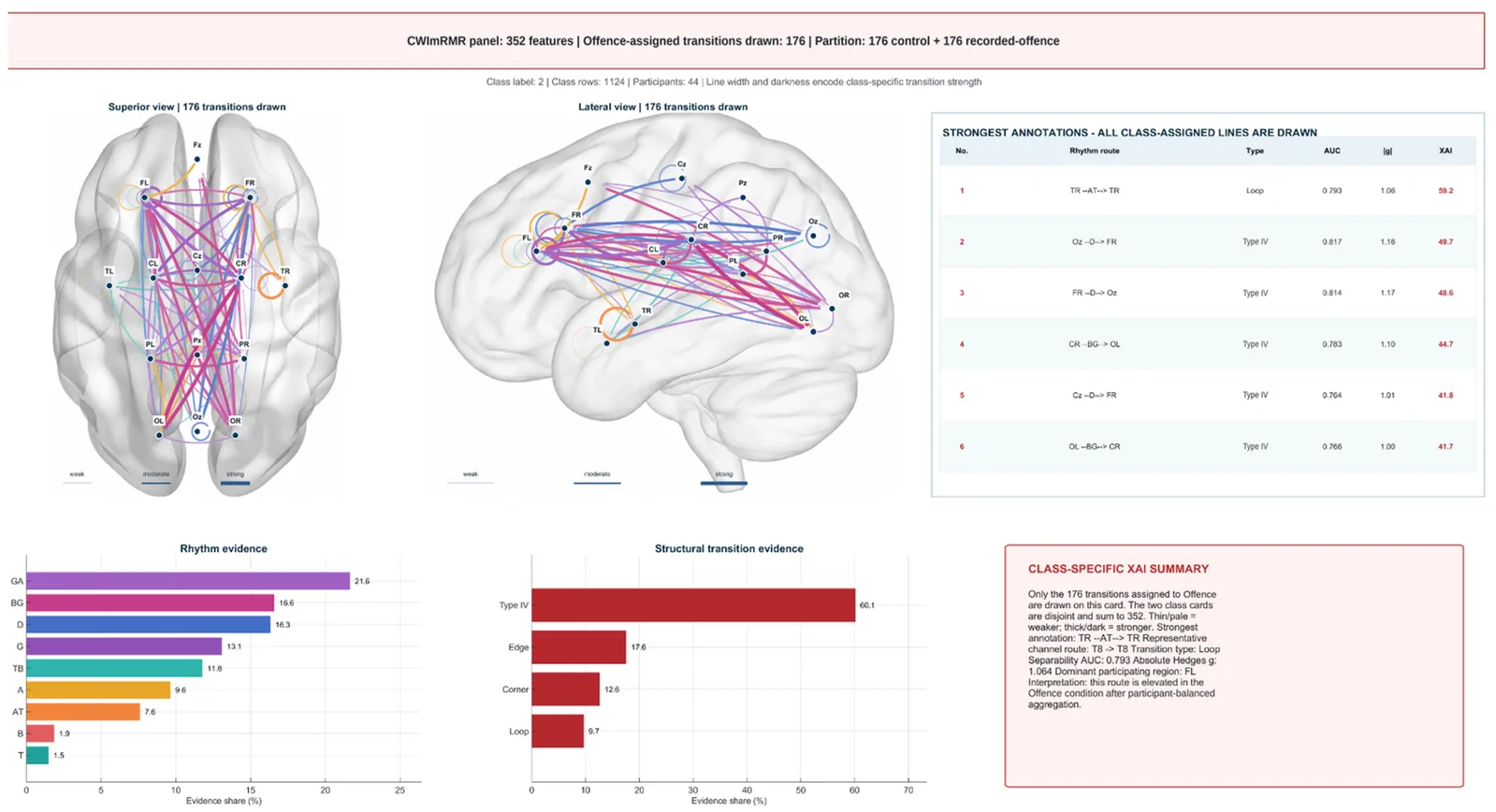
Offence-assigned DRLobish route card. The 176 selected transitions are shown with the same scaling as Figure 4. Type IV is a symbolic grammar label and does not measure structural or functional disconnection. In the graphic, ‘Offence’ denotes the recorded-offence group.

Reciprocal occipital-to-right-frontal and right-frontal-to-occipital delta routes were also among the highest-scoring coordinates. A right-central-to-left-occipital beta-to-gamma route was another prominent coordinate. The recorded-offence-assigned route card is presented in Figure 5.

Figure 5 shows that the recorded-offence-assigned panel contained a greater contribution from delta, beta-to-gamma and Type IV-labelled routes. These findings describe the composition of the selected feature panel. They do not establish that the displayed routes are specific biomarkers of violent offending.

The highest-ranked class-assigned routes are summarised in Table 4.

As presented in Table 4, the leading routes differed between the two class-assigned panels. Gamma-to-alpha and theta-related routes were present among the highest-ranked control coordinates. Delta and beta-to-gamma routes were more frequent among the highest-ranked recorded-offence coordinates.

The largest AUC in Table 4 was obtained by the recorded-offence-assigned Oz—D → FR route, with an AUC of 0.817. The reciprocal FR—D → Oz route achieved a similar AUC of 0.814. However, the final route order was not determined by AUC alone. The XAI score also incorporated effect size and CWImRMR feature-rank priority.

#### 3.3.2. Channel and Rhythm-Layer Distributions

**Figure 6:**
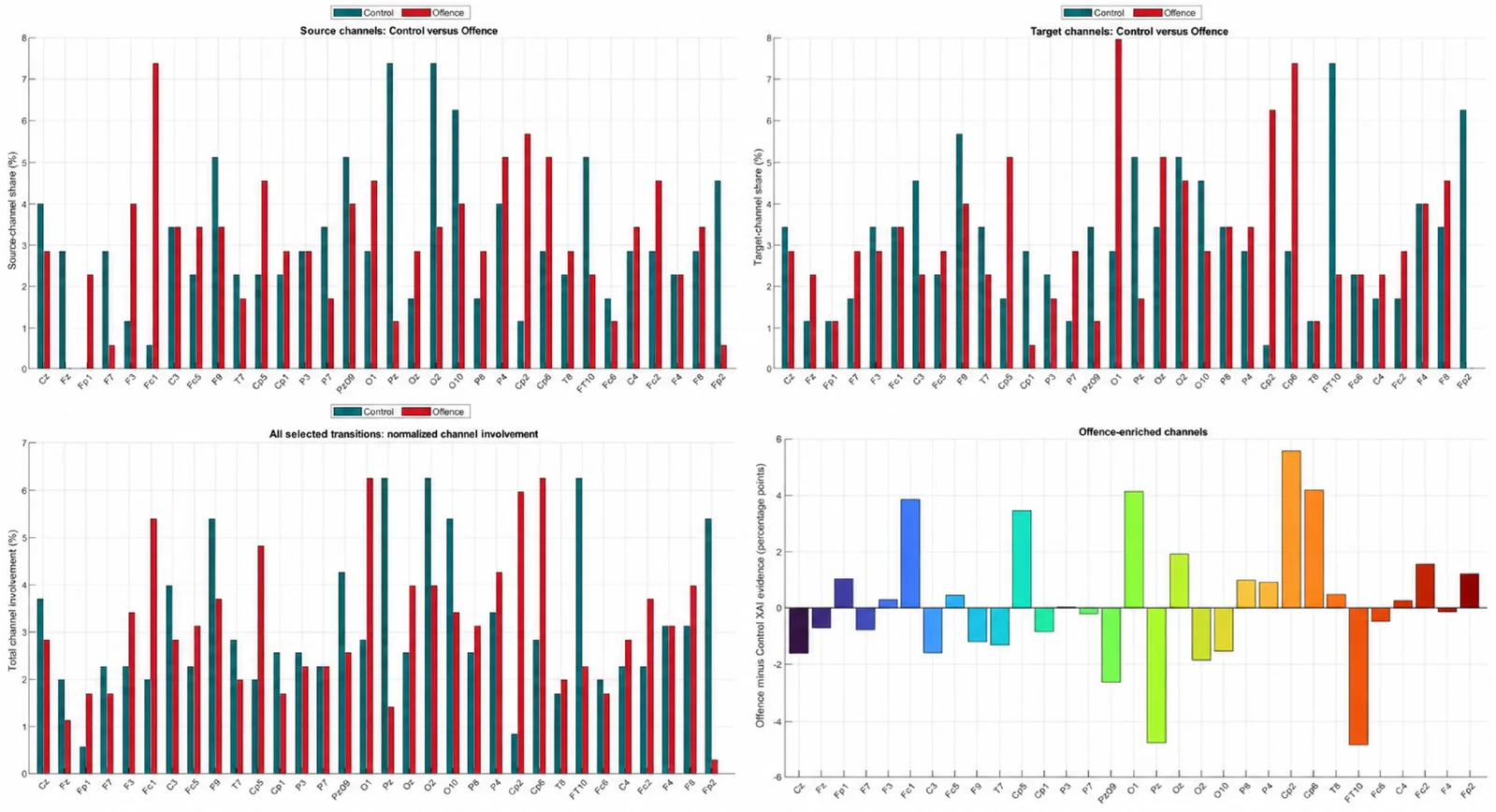
Channel involvement within the selected DRLobish routes. Source, target and combined involvement shares are normalised within each class. The final panel presents the recorded-offence-group minus control-group difference. In the graphic, ‘Offence’ denotes the recorded-offence group.

Channel-level summaries were calculated to preserve information that could be reduced during regional aggregation. The largest recorded-offence-group enrichments were observed for CP2, CP6, O1, FC1 and CP5. The largest control-group enrichments were observed for Fp2, FT10, Pz and PO9. The channel-involvement distributions are presented in Figure 6.

As shown in Figure 6, channel involvement differed between the two class-assigned panels. These values represent the proportional involvement of each channel within the 352 selected coordinates. They do not represent EEG amplitude, source-localised activation or inferential statistical significance.

Rhythm-layer distributions were also calculated separately for within-rhythm and cross-rhythm coordinates. Among the five within-rhythm layers, delta showed the largest recorded-offence group enrichment, with a difference of +10.46 percentage points. Theta was more prominent in the control-assigned panel, with a recorded-offence-minus-control difference of −11.24 percentage points.

Among the cross-rhythm layers, beta-to-gamma showed a recorded-offence-group enrichment of +5.47 percentage points. Theta-to-beta showed an enrichment of +3.29 percentage points. Alpha-to-theta was more prominent in the control-assigned panel, with a difference of −10.52 percentage points. Gamma-to-alpha contributed to both class-assigned panels.

**Figure 7:**
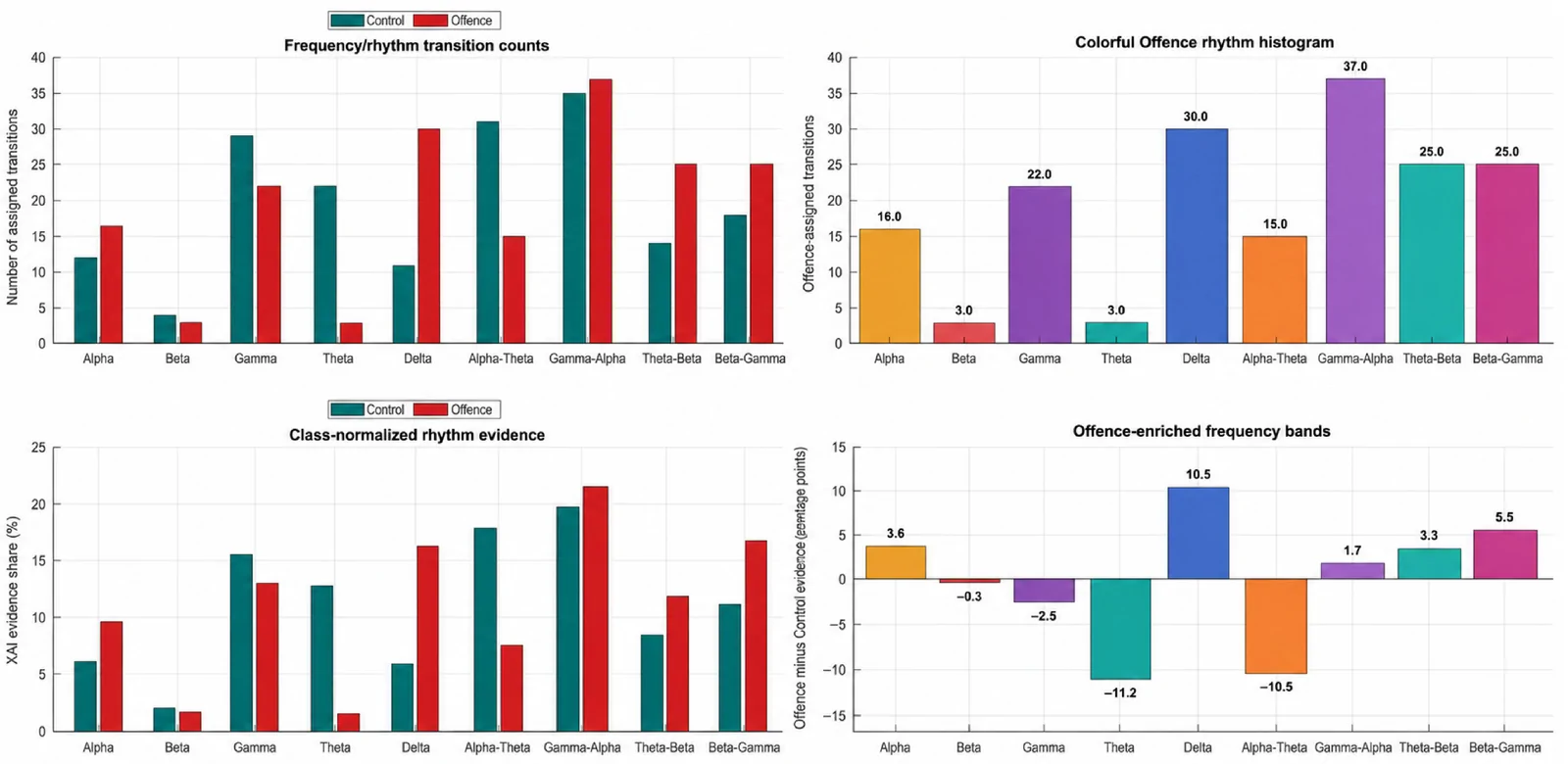
Rhythm-layer composition of the 352 selected transitions. The figure presents feature counts, within-class evidence shares and recorded-offence-group minus control-group differences. In the graphic, ‘Offence’ denotes the recorded-offence group.

The rhythm-layer composition of the selected coordinates is presented in Figure 7.

Figure 7 shows that the two class-assigned feature sets were equal in number but differed in rhythm composition. Control-assigned evidence was distributed mainly across gamma, theta, gamma-to-alpha and alpha-to-theta routes. Recorded-offence-assigned evidence contained a larger contribution from delta and beta-to-gamma routes.

The observed delta enrichment was interpreted cautiously because delta activity may be affected by vigilance, drowsiness, medication and slow artefacts. Therefore, the delta distribution was treated as a descriptive property of the selected panel rather than as a violence-specific EEG finding.

**Figure 8:**
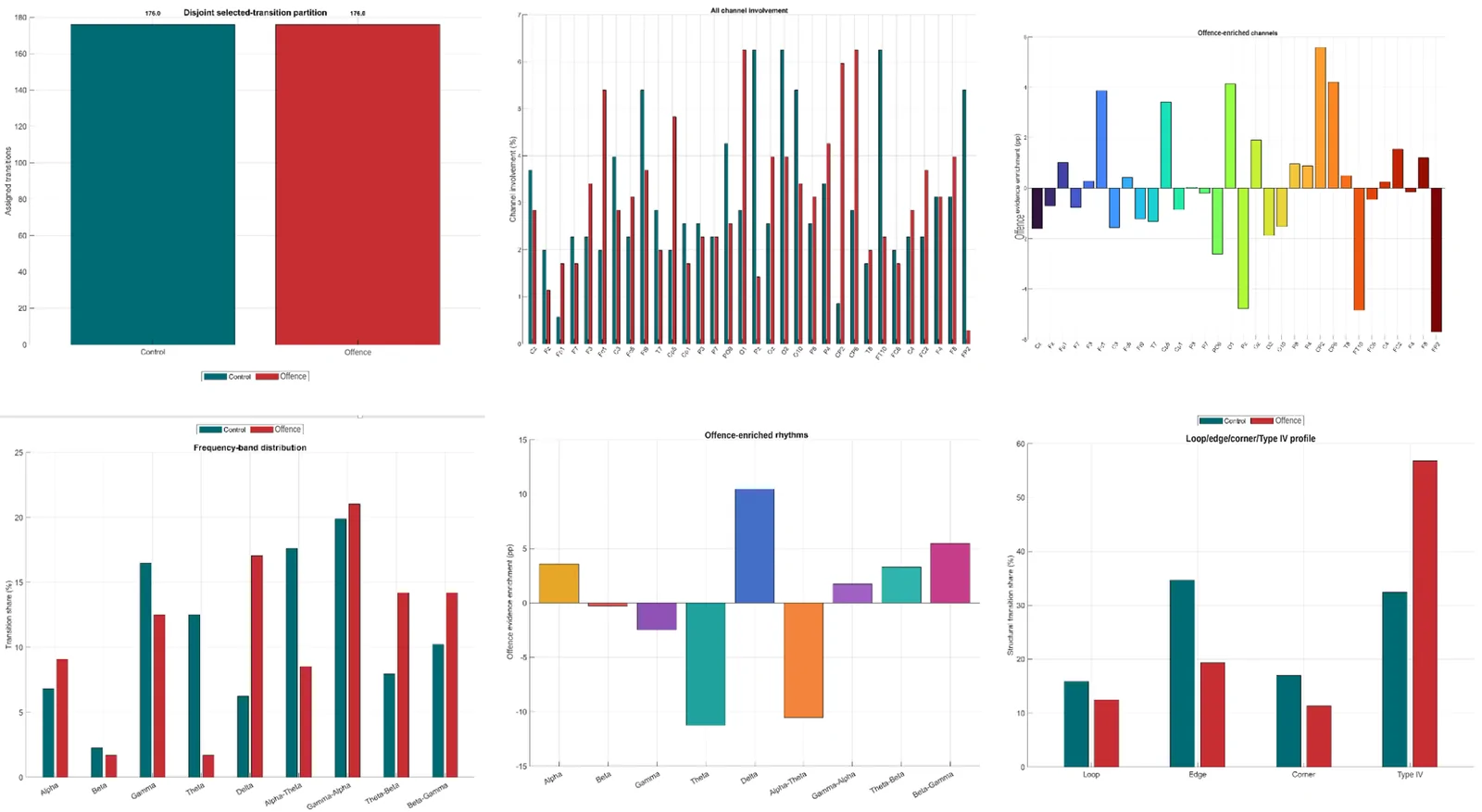
Cohort-level DRLobish dashboard. The panels summarise class assignment, channel involvement, rhythm-layer distributions, enrichment differences and symbolic structural grammar within the selected feature panel. In the graphic, ‘Offence’ denotes the recorded-offence group, and Type IV denotes a symbolic DRLobish grammar category.

The combined DRLobish summaries are presented in Figure 8.

As presented in Figure 8, the selected control- and recorded-offence-assigned coordinates differed across rhythm, channel and structural-grammar summaries. The regional distributions remained sensor-space descriptions. They cannot identify the cortical sources that generated the recorded EEG activity.

#### 3.3.3. Surrogate-Tree Audit

A compact decision-tree model was fitted as a lower-capacity audit of the selected feature space. The surrogate model used three of the 352 CWImRMR features.

Under participant-wise LOSO evaluation, the surrogate tree correctly classified 44 controls and 38 participants from the recorded-offence group. Seven controls were assigned to the recorded-offence group, and six recorded-offence-group participants were assigned to the control group. The resulting participant-level accuracy was 86.32%.

The full-cohort explanatory tree used three coordinates: delta FC2 → F8, gamma-to-alpha FT9 → CP6 and delta FT10 → FT10. The tree generated threshold-based decisions from these coordinates. All three coordinates involve a peripheral sensor (F8, FT9 or FT10), and one is a self-transition. The surrogate may therefore be sensitive to ocular or muscle activity.

The surrogate model was used only to assess whether a small subset of readable coordinates could approximate part of the class structure captured by the full feature panel. It did not replace the selected CWImRMR–tkNN classifier and was not treated as an externally calibrated participant-level decision rule.

#### 3.3.4. Descriptive Checks on Route Scoring and Selection Dependence

We quantified cross-selector agreement in the comparative analysis. The 352 CWImRMR features shared 9 coordinates with the 305 CWINCA features (Jaccard similarity 1.39%). They shared 44 coordinates with the 142 CWIChi2 features (9.78%) and 9 with the 202 CWIReliefF features (1.65%). Only one coordinate was kept by all four selectors: the delta Oz → Oz self-transition. Thus, the retained coordinates depend strongly on the selection criterion. These values are not repeat-sampling stability estimates for CWImRMR.

For the six leading routes in each class (Table 4), the Spearman rank correlations between XAI score and AUC were 0.143 for controls and 0.771 for the recorded-offence group; the corresponding correlations with absolute Hedges’ g were 0.986 and 0.600. These are descriptive checks on the six-route summaries, not weight-perturbation results for all 352 features. Route-level AUC and Hedges’ g values are reported descriptively; no route-wise hypothesis tests were carried out, so no multiple-comparison correction was required.

Finally, the three-feature surrogate tree achieved 86.32% participant-level classification accuracy in its own LOSO test, compared with 97.89% for the primary model. These two accuracies are not a direct fidelity metric, because fidelity requires paired predictions on the same participants. DRLobish is therefore described as reversible feature-level interpretation rather than as an explanation of each individual tkNN decision.

#### 3.3.5. Mathematical Bounds on Surrogate Prediction Agreement

The primary participant-level confusion matrix reported 51/51 controls and 42/44 recorded-offence participants correctly classified, whereas the three-feature surrogate correctly classified 44/51 controls and 38/44 recorded-offence participants. From these group-specific counts alone, 44 controls must have identical surrogate and primary predictions. Depending on how many of the two primary-model recorded-offence errors coincide with surrogate errors, agreement within the recorded-offence group may be 36, 38 or 40 participants. Thus the unobserved overall paired agreement is mathematically bounded between 80/95 (84.21%) and 84/95 (88.42%). These bounds are not an observed fidelity estimate and do not recover paired participant predictions.

### 3.4. Feature Structure

Each 32 × 32 layer contains 32 self-transitions, so 288 of the 9216 features (3.125%) are self-transitions. A random 352-feature panel would contain 11 of them on average (95% range 5–18). Features that touch at least one of FT9, FT10, Fp1, Fp2, F7, F8, T7 or T8 make up 4032 of the 9216 coordinates (43.75%); a random panel would contain 154 of them (95% range 136–172). Two leading coordinates in this study are peripheral self-transitions: T8 → T8 in the alpha-to-theta layer and FT10 → FT10 in the delta layer.

## 4. Discussion

The fixed-panel analysis yielded 93.66% epoch accuracy and 97.89% participant-majority accuracy. A nested SVM gave similar values (94.71%; 93/95 participants), and CWImRMR remained the best branch in the comparative feature-selection analysis (93.85%). Because the panel was selected with all participants, these values are internal estimates. RhythmFluxPat preserves rhythm and sensor coordinates, and DRLobish decodes them into readable sensor-space descriptions.

The principal contribution of the study was not limited to classification performance. The proposed framework established a traceable connection between the original EEG epoch, the selected feature index and the resulting source–rhythm–target expression. This structure allowed the classifier input space to be examined without relying on anonymous latent representations.

The two class-assigned feature panels differed in rhythm composition. As presented in Figure 4, Figure 5, Figure 6, Figure 7 and Figure 8, delta and beta-to-gamma routes were more prominent in the recorded-offence-assigned panel. Theta and alpha-to-theta routes were more prominent in the control-assigned panel. The selected coordinates also differed in their channel involvement and symbolic DRLobish grammars.

These findings should be interpreted as cohort-specific properties of the selected feature space. They do not establish a universal EEG signature of violent offending. The recorded-offence group was recruited from a psychiatry service, while the recruitment source of the control group was unavailable. The two groups also differed in mean age and in the range of usable EEG epochs. Medication, sedation, psychiatric diagnosis, substance exposure, vigilance and other clinically relevant variables were not available. The comparison is between psychiatric-service adults with recorded offences and non-clinical adults. Psychiatric status and treatment are part of the contrast, so an EEG difference may reflect psychiatric illness or its treatment rather than offence history.

The delta result is especially sensitive to these limitations. Delta activity may be affected by drowsiness, reduced vigilance, medication, sedation and slow artefacts. The larger delta contribution in the recorded-offence-assigned panel may therefore reflect a combination of clinical state, vigilance, age and recording characteristics. It should not be interpreted as a violence-specific neural marker.

The distributed involvement of frontal, central, temporal, parietal and occipital channels also requires cautious interpretation. These sensor-space routes may reflect differences in attention, arousal, visual processing, reference effects or treatment state. DRLobish identifies the coordinates used to describe the selected feature panel, but it does not identify the cortical generators of the recorded signals.

The initial classifier-selection analysis (94.01%) and the fixed-panel evaluation (93.66%) share the same cohort-wide feature selection. The two values are therefore optimistic in the same way, and neither establishes generalisability. The 14-year age difference is a second and independent threat (Section 2.1).

Within these boundaries, the findings support the methodological value of the proposed HumanForensics framework. Neurocriminology defines the scientific question concerning brain–behaviour associations. HumanForensics provides traceability, readable computational evidence and explicit restrictions on interpretation. The framework does not convert classification performance into a claim of a criminal brain phenotype.

### 4.1. Biological, Psychiatric and Digital-Forensic Interpretation

The selected routes are candidate EEG features for retrospective cohort comparison. Their biological relevance lies in the rhythm and sensor coordinates they retain, which permit specific physiological hypotheses to be tested. Delta and beta-to-gamma routes were more prominent in the recorded-offence-assigned panel, whereas theta and alpha-to-theta routes were more prominent in the control-assigned panel. These differences concern the composition and descriptive weighting of selected transitions. They do not directly demonstrate increased delta power, reduced theta power or stronger coupling between rhythms. A discriminative coordinate becomes a credible biomarker only when its measurement is reproducible and its association with a defined biological or clinical outcome survives independent validation.

One plausible explanation for the delta pattern is a difference in vigilance during passive viewing. Slow cortical and thalamocortical activity changes with the sleep–wake state; greater synchronisation of slow activity can accompany reduced arousal. This provides a physiological hypothesis for the prominence of delta-derived transitions, rather than evidence that the participants were drowsy. Delta activity can also occur during wakefulness. Medication, clinical state and slow ocular or movement artefacts provide alternative explanations. Sleep staging, vigilance measures and artefact-controlled spectral analysis would be needed to determine whether the selected delta coordinates track these processes. The present transition features alone cannot identify their physiological cause.

Theta and alpha-to-theta routes were more prominent in the control-assigned feature panel. Previous studies have associated meditation and mindfulness-related conditions with changes in theta and alpha activity [12,13,14]. A plausible interpretation is variation in sustained attention, sensory gating and engagement with the audiovisual stimulus. Theta-related activity may accompany attentional control, whereas alpha modulation may reflect how sensory input is selected or suppressed. These processes can vary even when participants receive the same stimulus. Mindfulness-related oscillatory changes also need not be driven by arousal alone. Here, passive viewing did not establish a meditative state, and no behavioural measure of attention was collected. The alpha-to-theta label denotes an ordered feature transition; it does not demonstrate alpha–theta coupling or superior inhibitory control.

The beta-to-gamma pattern may reflect differences in the temporal organisation of fast activity during sensory engagement. Neural gamma activity arises through interactions between excitatory and inhibitory neuronal populations, but a scalp-derived beta-to-gamma transition does not measure that balance. It therefore cannot establish cortical hyperexcitability or a neurotransmitter abnormality. A second explanation is residual cranial muscle activity, which can contribute substantially to scalp signals in the beta and gamma ranges. This possibility is relevant to temporal and peripheral sensors. Analyses controlling for muscle contamination and separating oscillatory peaks from broadband activity would help distinguish neural from non-neural contributions. RhythmFluxPat does not estimate phase–amplitude coupling or effective connectivity.

The regional pattern is compatible with several physiological processes. Occipital involvement may reflect visual input, while frontal and central involvement may relate to attention and task state during viewing. Thus, differences in sensory engagement could contribute to the occipital–frontal delta and central–occipital beta-to-gamma coordinates. These are testable explanations, not identified cortical pathways. Scalp electrodes record mixtures of generators, and reference choice and volume conduction can affect apparent interchannel relationships. A reciprocal sensor route therefore does not demonstrate bidirectional cortical communication. Source-resolved EEG and an attention-controlled task would be needed to evaluate an anatomical or functional interpretation.

Type IV accounted for 60.11% of the symbolic grammar in the recorded-offence-assigned panel. This percentage describes selected routes, not the proportion of participants with a biological abnormality. Type IV is a code-defined category and does not indicate tissue damage or disrupted connectivity. Likewise, a large route-level AUC, effect size or XAI score identifies a useful descriptive feature within this cohort; it does not establish biological specificity. The leading delta and cross-rhythm coordinates are therefore priorities for replication. Their value as candidate biomarkers depends on reproducibility, stability across recordings and selectors, and an association that remains after demographic, clinical and recording factors are controlled.

The intended biomarker outcome must also be defined. Recorded-offence status is a historical group label, not a direct measure of current aggression, inhibitory control or future violence. A reproducible EEG association with this label could still reflect age, treatment, psychiatric state or vigilance. To support a biological interpretation, the same prespecified coordinates should be tested against independently measured attention, arousal or behavioural control. To support clinical utility, they should add information beyond demographic and clinical predictors in an external cohort. The present results identify candidate features for these tests; they do not establish a biomarker of an individual behavioural disposition.

From a psychiatric perspective, the contrast is not simply between offenders and non-offenders. The recorded-offence group came from a psychiatry service, whereas controls had no prior clinical diagnosis, so psychiatric status and treatment are built into the group comparison. This matters for the observed pattern. Sedating and antipsychotic medications can increase slow-wave activity, and benzodiazepines are known to raise fast beta activity, so the delta and beta-to-gamma routes could partly reflect treatment rather than offence history. Disorders that are common in forensic psychiatric settings, including substance use, psychotic, mood and personality disorders, also have their own EEG correlates. The most informative next comparison would therefore include psychiatric patients without violent offences, assessed with structured diagnostic interviews, medication records and standardised measures of aggression and impulsivity. Even then, an EEG pattern would serve at most as an adjunct to structured clinical assessment, not as a substitute for it.

From a digital-forensics perspective, the main strength of the framework is its audit trail. Each selected feature can be traced back to a rhythm, a source sensor and a target sensor, and each participant decision can be traced back to stored epoch predictions. Forensic soundness, however, needs more than traceability. Raw recordings would require integrity checks, documented provenance and a chain of custody, and every processing step would need fixed software versions and complete logs. The present run does not meet these requirements: the MATLAB release was not recorded, and complete candidate-level inner results were kept for only one outer fold. Scientific evidence is also judged by its known error rate in independent samples and by accepted operating procedures, and neither exists yet for this model. Neural recordings are sensitive personal data as well, so any applied use would need strict access control and data-protection safeguards. For these reasons, the framework is offered as a transparent research tool, not as a digital-forensic method for casework.

The findings are relevant to retrospective group-level EEG analysis. They are not suitable for individual diagnosis, future-violence prediction, sentencing, parole assessment or attribution of legal and moral responsibility. Explainability improves computational transparency, but it does not establish external validity, fairness or suitability for high-stakes forensic decision-making [32].

### 4.2. Comparative Results

The four feature-ranking methods produced distinct feature panels. Table 5 presents the comparative evaluation of the four selectors and their six pairwise intersections. Table 6 reports IMV ensembles of the ranked branches. All reported values were calculated from the held-out predictions (Appendix D). Each branch value is the best of several prefix and classifier settings. It is therefore open to selection optimism. The participant counts are not adjusted for this selection.

**Figure 9:**
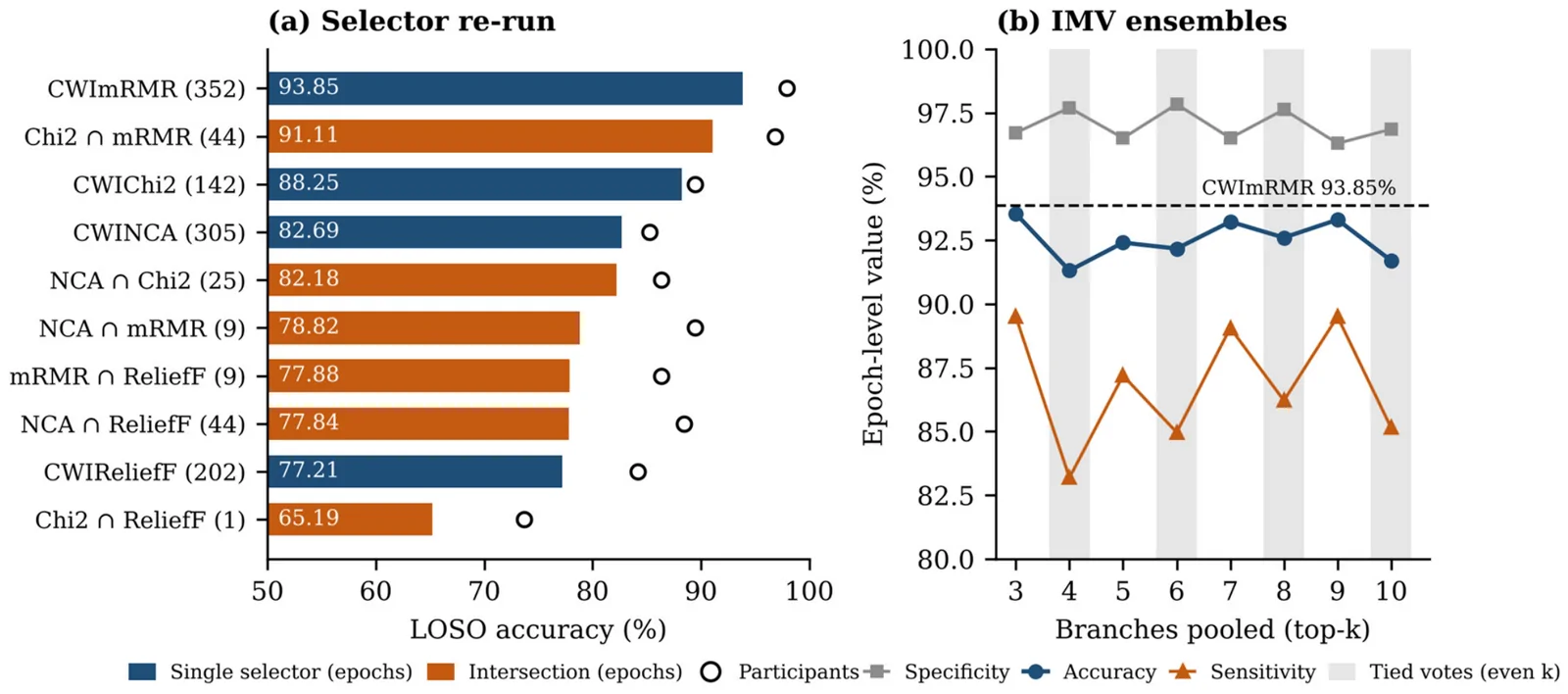
Comparison of feature-selection methods. (**a**) Epoch accuracy (bars) and participant accuracy (circles) for the four selector panels and the six intersection panels; feature counts are given in brackets. (**b**) IMV of the 3 to 10 best branches. The dashed line marks CWImRMR alone, and grey bands mark even-sized ensembles with tied votes. All panels were selected with full-cohort labels.

As shown in Table 5 and Figure 9a, CWImRMR achieved the highest accuracy among the evaluated feature-selection methods. It classified 2397/2554 epochs correctly (93.85%; participant-cluster bootstrap 95% CI 91.26–95.99%) with 352/9216 features (3.82%). This is 4 epochs (0.16 percentage points) below the selected-model accuracy of 94.01% for the same panel. Majority voting classified 93/95 participants correctly (97.89%). Two of these votes were 50:50 ties, one in each group. The mode rule assigned both ties to the control class. If ties are counted as errors, 92/95 participants were correct. All 95 participants contributed to panel selection. These values therefore do not show unbiased comparative performance.

The 44 coordinates shared by CWIChi2 and CWImRMR classified 2327/2554 epochs (91.11%) and 92/95 participants (96.84%). This is 2.74 percentage points below the full panel at epoch level. CWIChi2 kept 142 features and reached 88.25%. CWINCA kept 305 features and reached 82.69%. CWIReliefF kept 202 features and reached 77.21%. The two nine-coordinate intersections reached 78.82% (NCA ∩ mRMR) and 77.88% (mRMR ∩ ReliefF). Both were at least 15.04 percentage points below CWImRMR. The single delta Oz → Oz coordinate (Chi2 ∩ ReliefF) reached 65.19%.

Panel size alone did not set accuracy. The 44-coordinate Chi2 ∩ mRMR panel outperformed the 142-, 202- and 305-coordinate single-selector panels. Panels with nine or fewer coordinates lost much of the separation. The four ranking criteria also kept very different feature sets.

IMV did not improve on the best single branch (Table 6; Figure 9b). The top-3 ensemble pooled CWImRMR, Chi2 ∩ mRMR and CWIChi2. It classified 2389/2554 epochs (93.54%) and 92/95 participants (96.84%). This is 8 epochs fewer than CWImRMR alone. Its predictions differed from CWImRMR on 142 epochs. It corrected 67 errors and introduced 75 new ones. Ensembles of 3 to 10 branches reached 91.31–93.54% epoch accuracy and 89–92 correct participants. Even-sized ensembles produced 107–145 tied epoch votes. The mode rule assigned these ties to the control class. Their recorded-offence sensitivity was therefore lower (83.19–86.21%) than that of odd-sized ensembles (87.19–89.50%).

Feature overlap between the panels was limited. CWIChi2 and CWImRMR shared 44 features. CWINCA and CWImRMR shared nine, as did CWIReliefF and CWImRMR. Only the delta Oz → Oz self-transition was common to all four selectors. The retained EEG coordinates therefore depended strongly on the ranking criterion.

Therefore, the presence of a coordinate in one selected panel should not be interpreted as evidence of feature stability. The selected routes describe the present development analysis. Their stability would require repeated selection analysis or independent validation.

The reported differences between the selectors also represent observed development-stage performance. Feature count and tkNN configuration were optimised within the same classifier-stage LOSO stream. Accordingly, Table 5 and Table 6 compare the behaviour of the selection branches within the present cohort rather than their unbiased generalisation to an independent sample.

Direct accuracy comparisons with previous aggression- or offence-related EEG studies were not performed. Earlier studies used different participant groups, clinical conditions, EEG systems, recording tasks, preprocessing procedures and validation units. Their findings provide neurobiological and methodological context, but they do not form a directly comparable performance benchmark.

The present analyses cannot show whether RhythmFluxPat improves on conventional spectral features. Code for a matched comparison with absolute, relative and combined band power is provided in Supplementary Code S1. It uses the same fold-internal selection and classifier search. This comparison is required before any claim of higher accuracy. In this study, the contribution of RhythmFluxPat is the traceability of its inputs, not a proven accuracy gain over band power.

### 4.3. Interpretation and Use Boundaries

The fixed-panel result was 93.66% epoch accuracy and 97.89% participant-majority accuracy, and the initial classifier-selection accuracy was 94.01%. Both depend on cohort-wide feature selection and are optimistic (Appendix B). Classifier nesting does not remove the age imbalance, the psychiatric-service recruitment or unmeasured clinical confounding.

Epoch-level and participant-level results describe different aspects of model behaviour. Epoch-level accuracy reflects the consistency of classification across individual 15 s EEG windows. Participant-level accuracy reflects the dominant prediction obtained through majority voting across the available epochs of each participant. The participant-level result is therefore an aggregation of epoch predictions rather than an independent validation experiment.

All 95 participants were included in the participant-level evaluation. No participant was excluded from majority voting, and no abstention rule was applied. The equal assignment of 176 selected features to each class emerged from participant-balanced class means after feature selection. This equal distribution was not imposed during model training and does not imply biological symmetry between the groups.

The three-feature surrogate tree provided a lower-capacity representation of the selected feature space. Its participant-level LOSO accuracy of 86.32% was lower than that of the full CWImRMR–tkNN model. The surrogate therefore served only as an explanation audit and was not treated as a replacement classifier.

DRLobish routes represent reversible descriptions of sensor-space feature coordinates. They do not correspond to anatomical tracts, cortical sources, effective connectivity or causal neural pathways. Similarly, the loop, edge, corner and Type IV labels are elements of the symbolic reporting grammar and should not be interpreted as anatomical structures or disconnection patterns.

The DRLobish route scores were calculated through fixed and transparent combinations of participant-balanced AUC, absolute Hedges’ g, feature-rank priority and class-specific mean values. These scores were used to organise and describe the selected feature panel. They were not designed as clinical thresholds, diagnostic scores or calibrated measures of individual risk.

Neurocriminology defines the scientific context of the study, while HumanForensics provides the computational framework for traceability and readable evidence. Neither concept supports automated forensic decision-making. The model should not be used to diagnose an individual, estimate future violence, determine dangerousness, guide sentencing or parole decisions, or infer moral and legal responsibility.

### 4.4. Limitations and Future Work

Feature ranking and prefix selection used all 95 participants before the outer LOSO split; only classifier choice was nested. This design can inflate accuracy (Appendix B). The reported accuracies are therefore optimistic. Their size cannot be separated from selection artefact without a fully nested analysis of the real data with a full-pipeline permutation test. This validation procedure is a necessary next step [23,24,25,26], and the code for it is provided in Supplementary Code S1. A nested SVM gave similar results (Section 3.2). The separation therefore does not depend on the tkNN search. It still carries the feature-selection optimism. The matched band-power comparison (Section 4.2) is the second required step.

The recorded-offence group was on average 14 years older than controls (44.5 versus 30.5 years). Although age was not an explicit model input, age-related EEG variation could contribute to the observed separation. No age-matched, age-adjusted or age-stratified analysis was completed. Medication, psychiatric diagnosis, sedation, substance exposure, sleep, vigilance and the control recruitment pathway were also not adequately documented. The observed differences therefore cannot be attributed uniquely to recorded-offence status. The recorded-offence group also came from a psychiatry service, whereas controls were non-clinical; this contrast is part of the design.

The number of usable epochs also differed between groups. Control participants contributed between 28 and 30 epochs, whereas participants in the recorded-offence group contributed between 7 and 28 epochs. This difference may reflect variation in recording yield, data quality or artefact burden. One of the two participant-level errors involved a participant with only 8 usable epochs. Visibly contaminated recordings were reacquired or excluded, but quantitative artefact scores and participant-level rejection counts were not recorded.

These limitations are particularly relevant to the observed delta enrichment. Delta activity may be influenced by age, drowsiness, reduced vigilance, medication, sedation and slow artefacts. Therefore, the higher delta contribution in the recorded-offence-assigned feature panel cannot be interpreted as a violence-specific EEG pattern. RhythmFluxPat mainly encodes amplitude-rank persistence at short lags, and its layers contain many self-transitions. Peripheral sensors that often carry ocular and muscle artefacts contribute to leading coordinates. These properties may contribute to the observed separation.

The experimental condition also imposed limitations. A common meditation-video excerpt was used to standardise audiovisual exposure, but attention, relaxation and meditative engagement were not assessed. The exact start and end points of the excerpt, presentation medium, display settings and sound level were unavailable. Consequently, the internal cognitive state produced by the stimulus could not be verified or compared between participants. The nominal stimulus duration also differed from the duration implied by the maximum retained epoch count (Section 2.1).

The following analyses are not supported by the available data export: end-to-end nested feature ranking and prefix selection on the original EEG feature matrix; a complete participant-label permutation test; observed age-adjusted or age-matched EEG comparisons; matched nested band-power baselines; feature extraction across the complete cohort (feature extraction was independently verified for two epochs; Appendix C) and ablation of delta, peripheral-channel, self-transition and Algorithm 1 step-6 components; independent replication, model calibration and decision-curve analysis. These tests require the original feature matrix, fold-wise training logs or new matched clinical cohorts; they have not been replaced by model-based estimates or simulations. Its intersection panels and the IMV ensembles also use full-cohort selection. They do not remove this bias.

This cross-sectional, retrospective study examined group separation within one cohort. It did not test prospective violence prediction, recidivism or incremental value beyond clinical and demographic information. Prospective validation remains limited in research on violence neuroprediction [21].

Biomarker validation should therefore proceed from measurement to mechanism and then to utility. Repeated recordings should test coordinate stability; vigilance and artefact measures should test the proposed physiological explanations; matched or adjusted external cohorts should test specificity and incremental value. Until these steps are completed, the routes remain candidate EEG features for retrospective cohort comparison.

## 5. Conclusions

RhythmFluxPat represents each EEG epoch through reversible source–rhythm–target coordinates. CWImRMR reduced 9216 features to a 352-feature development panel, which was used for participant-held-out tkNN classification. This workflow supports transparent retrospective EEG comparison.

With the 352-feature panel fixed, classifier-nested participant-held-out LOSO correctly classified 2392/2554 epochs (93.66%) and 93/95 participants (97.89%). All 51 controls and 42 of 44 recorded-offence participants were correctly classified by majority vote. These exported outcomes were independently verified, but the panel had been selected with all 95 participant labels; the accuracies must therefore be interpreted as optimistic internal results. A nested SVM under the same folds gave similar values (94.71% of epochs; 93/95 participants). The result therefore does not depend on the classifier family. In the comparative feature-selection analysis, CWImRMR remained the best branch (2397/2554 epochs; 93.85%). Neither the six intersection panels nor IMV exceeded it.

DRLobish decoded the selected inputs into sensor-space rhythm routes. Delta and beta-to-gamma routes were more prominent in the recorded-offence-assigned panel; theta and alpha-to-theta routes were more prominent in the control-assigned panel. Their biological relevance can be tested through hypotheses concerning vigilance, attention and sensory engagement. These patterns describe selected transitions rather than measured changes in band power, cortical connectivity or individual classifier decisions. They provide candidate coordinates for biomarker validation, not validated biological markers. At the short lags used, delta-layer routes index rank persistence.

DRLobish provides feature-level traceability. Cross-selector Jaccard overlaps and leading-route correlations are descriptive checks, not full-panel weight perturbation or resampling stability tests.

The identified routes represent cohort-specific sensor-space associations. They do not establish anatomical connectivity, causal neural mechanisms or a universal EEG signature of violent offending. The model should not be used for individual diagnosis, future-violence prediction or legal decision-making.

The cohort-wide supervised feature ranking and prefix selection were not repeated within outer folds; a fully nested end-to-end real-data estimate and participant-level permutation test remain unavailable. The groups also differ by about 14 years in age and combine psychiatric-service status with recorded-offence status. Consequently, independent generalisation and offence-specific biomarkers cannot be inferred from this cohort.

## Figures and Tables

**Table 1:** Cohort characteristics, demographic information and evaluable EEG units.

Characteristic	Control Group	Recorded-Offence Group	Total
Participants enrolled	51	44	95
Women	6	4	10
Men	45	40	85
Age range, years	19–42	22–67	19–67
Age, mean ± SD, years	30.51 ± 5.85	44.48 ± 11.33	36.98 ± 11.22
15 s EEG epochs	1430	1124	2554
Median epochs per participant	28	28	28
Range of epochs per participant	28–30	7–28	7–30
Participant majority-vote decisions	51	44	95
Participant-level classification errors	0	2	2

**Table 2:** RhythmFluxPat rhythm layers, frequency ranges and feature-index intervals.

Layer	Code	Rhythm Transition	Feature Indices
1	A	Alpha (8–13 Hz) -> alpha	1–1024
2	B	Beta (13–30 Hz) -> beta	1025–2048
3	G	Gamma (30–80 Hz) -> gamma	2049–3072
4	T	Theta (4–8 Hz) -> theta	3073–4096
5	D	Delta (0.5–4 Hz) -> delta	4097–5120
6	AT	Alpha (8–13 Hz) -> theta (4–8 Hz)	5121–6144
7	GA	Gamma -> alpha	6145–7168
8	TB	Theta -> beta	7169–8192
9	BG	Beta -> gamma	8193–9216

**Table 3:** Fixed-panel classifier-nested leave-one-subject-out (LOSO) performance at epoch and participant levels. The 352-feature panel was chosen using the full cohort; therefore, both estimates are optimistic.

Evaluation Level	Accuracy	Recorded-Offence Sensitivity	Control Specificity	Balanced Accuracy
Epochs (n = 2554)	93.66%	91.64%	95.24%	93.44%
Participants (n = 95)	97.89%	95.45%	100.00%	97.73%

**Table 4:** Highest-ranked class-assigned DRLobish routes according to participant-balanced AUC, absolute Hedges’ g and XAI evidence score.

Class	Rank	Decoded Route	Grammar	AUC	|g|	XAI Score
Control	1	CR—GA → CR	Loop	0.770	1.02	42.3
Control	2	Pz—T → OR	Type IV	0.763	0.98	40.8
Control	3	OL—TB → PL	Edge	0.757	0.97	40.2
Control	4	PL—GA → PL	Loop	0.738	0.76	39.8
Control	5	FR—D → FR	Loop	0.735	0.72	39.7
Control	6	CR—G → CR	Loop	0.775	0.72	39.0
Recorded-offence	1	TR—AT → TR	Loop	0.793	1.06	59.2
Recorded-offence	2	Oz—D → FR	Type IV	0.817	1.16	49.7
Recorded-offence	3	FR—D → Oz	Type IV	0.814	1.17	48.6
Recorded-offence	4	CR—BG → OL	Type IV	0.783	1.10	44.7
Recorded-offence	5	Cz—D → FR	Type IV	0.764	1.01	41.8
Recorded-offence	6	OL—BG → CR	Type IV	0.766	1.00	41.7

**Table 5:** Comparative evaluation of feature-selection methods and their pairwise intersections under participant-held-out LOSO (2554 epochs; 95 participants), ordered by epoch accuracy. All panels were selected with full-cohort labels, so the values are optimistic development-stage estimates. Sensitivity refers to the recorded-offence group and specificity to the control group. Participant votes follow the MATLAB mode rule, which assigns a 50:50 tie to the control class. The last column counts tied votes.

Panel	Features	Correct Epochs (Accuracy)	Sensitivity/Specificity (%)	Balanced Accuracy	Correct Participants (Accuracy)	Tied Votes
CWImRMR	352	2397 (93.85%)	92.17 / 95.17	93.67%	93 (97.89%)	2
Chi2 ∩ mRMR	44	2327 (91.11%)	88.70 / 93.01	90.85%	92 (96.84%)	0
CWIChi2	142	2254 (88.25%)	82.56 / 92.73	87.64%	85 (89.47%)	1
CWINCA	305	2112 (82.69%)	74.64 / 89.02	81.83%	81 (85.26%)	0
NCA ∩ Chi2	25	2099 (82.18%)	79.18 / 84.55	81.86%	82 (86.32%)	0
NCA ∩ mRMR	9	2013 (78.82%)	71.62 / 84.48	78.05%	85 (89.47%)	3
mRMR ∩ ReliefF	9	1989 (77.88%)	70.46 / 83.71	77.08%	82 (86.32%)	2
NCA ∩ ReliefF	44	1988 (77.84%)	72.33 / 82.17	77.25%	84 (88.42%)	3
CWIReliefF	202	1972 (77.21%)	74.02 / 79.72	76.87%	80 (84.21%)	4
Chi2 ∩ ReliefF	1	1665 (65.19%)	56.58 / 71.96	64.27%	70 (73.68%)	2

**Table 6:** Iterative majority voting (IMV) of the ranked branches in Table 5. Each ensemble pools the k best branch predictions. The mode rule assigns tied votes to the control class. The values inherit the full-cohort selection of the pooled panels.

Ensemble	Branches Pooled	Correct Epochs (Accuracy)	Sensitivity/Specificity (%)	Correct Participants (Accuracy)	Tied Epoch Votes
Top-3	CWImRMR, Chi2 ∩ mRMR, CWIChi2	2389 (93.54%)	89.50/96.71	92 (96.84%)	0
Top-4	+CWINCA	2332 (91.31%)	83.19/97.69	89 (93.68%)	145
Top-5	+NCA ∩ Chi2	2360 (92.40%)	87.19/96.50	89 (93.68%)	0
Top-6	+NCA ∩ mRMR	2354 (92.17%)	84.96/97.83	90 (94.74%)	124
Top-7	+mRMR ∩ ReliefF	2381 (93.23%)	89.06/96.50	90 (94.74%)	0
Top-8	+NCA ∩ ReliefF	2365 (92.60%)	86.21/97.62	90 (94.74%)	107
Top-9	+CWIReliefF	2383 (93.30%)	89.50/96.29	91 (95.79%)	0
Top-10	+Chi2 ∩ ReliefF	2342 (91.70%)	85.14/96.85	91 (95.79%)	109

**Figure A4:**
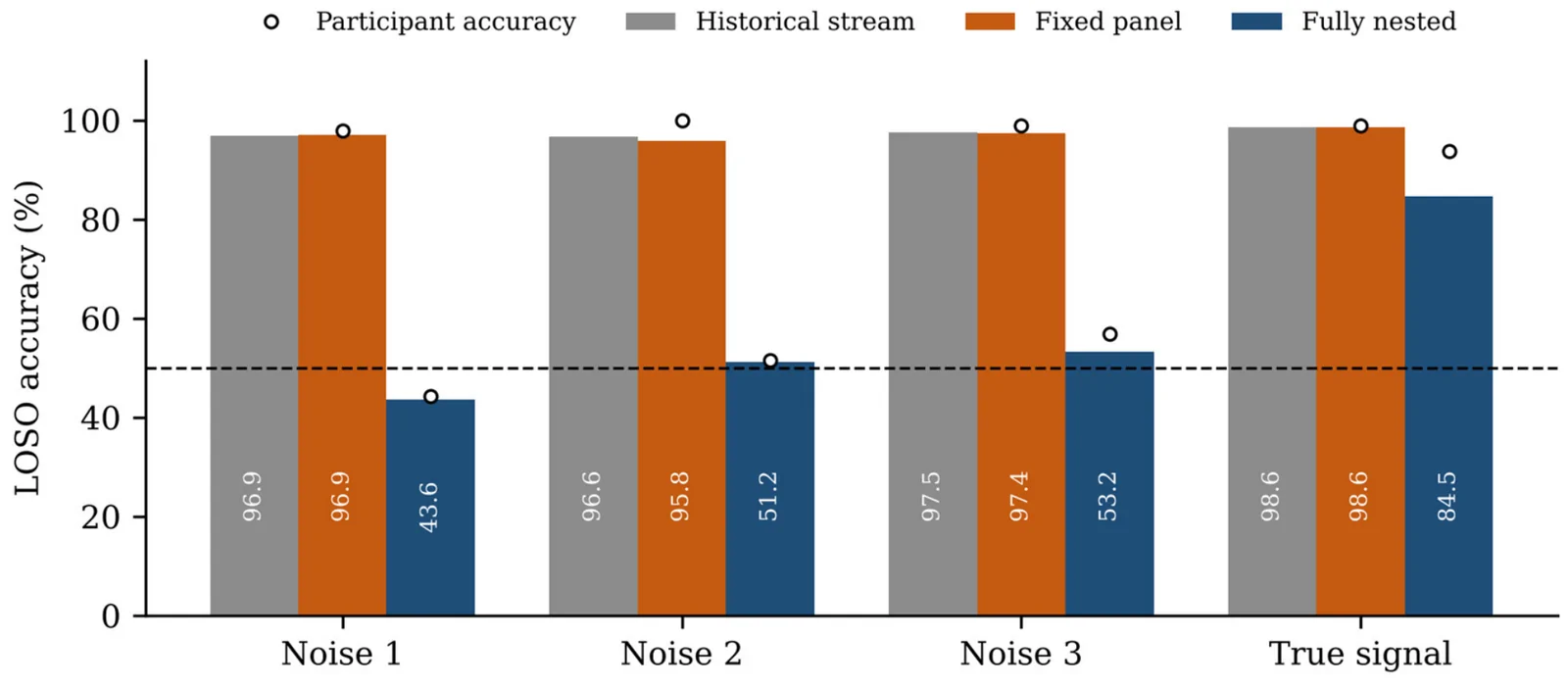
Leakage simulation with the cohort structure of this study. Bars show epoch accuracy and circles show participant accuracy for the initial classifier-selection procedure, the fixed-panel classifier-nested scheme and the fully nested scheme, on three pure-noise data sets and one data set with a true group effect. The dashed line marks chance (50%). Simulated data only; no study data were used.

**Figure A5:**
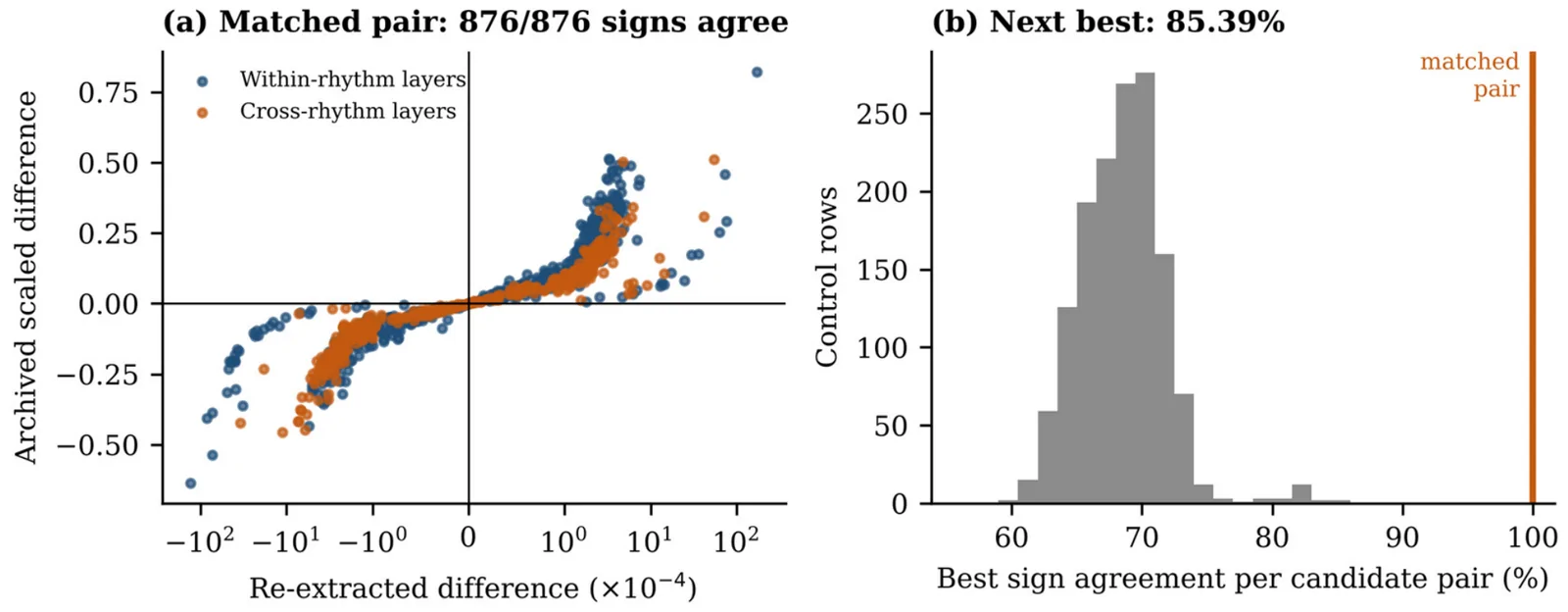
Trace of two raw epochs in the saved panels. (**a**) Independently extracted versus saved scaled between-epoch differences for the 876 selected coordinates; every point lies in the first or third quadrant (symmetric log scale on the horizontal axis). (**b**) Best sign agreement for every other candidate row pair. The matched pair reaches 100%; the next best reaches 85.39%.

## Data Availability

De-identified EEG data may be requested from the corresponding author, subject to participant consent, ethical approval, and institutional restrictions. The Supplementary Archive, contains eight Python scripts for RhythmFluxPat feature extraction, analysis of archived MATLAB (2023b) results, tkNN classification, classifier-nested SVM evaluation, performance verification, raw-epoch structural checks, and generation of supporting tables and figures. The scripts include procedures for generating participant-level classification records and Figure 9, Figure A3 and Figure A5. The archive does not include the original EEG recordings, feature matrices, saved prediction files, MATLAB (2023b) source code, or precomputed supplementary tables. These materials are therefore not represented as directly downloadable Supplementary Files. The Python scripts are intended for use with Python 3.11 and require the corresponding input data for full execution.

## Supplementary Materials

The following supporting information can be downloaded at https://www.mdpi.com/article/10.3390/diagnostics16213585/s1, Table S1: Per-participant classification results for the nested support-vector machine (SVM; one outer fold per participant) and the CWImRMR-based tkNN evaluation (Table 5). Both analyses used the fixed 352-feature panel selected using full-cohort class labels. Participant-level predictions were determined by majority voting according to the MATLAB mode rule. In the event of a 50:50 tie, the control class was assigned, and the result was marked as “tie”; Supplementary Code S1.

## Abbreviations

The following abbreviations are used in this manuscript:

EEG	Electroencephalography
NCA	Neighbourhood Component Analysis
mRMR	Minimum Redundancy Maximum Relevance
CWImRMR	Cumulative-Weighted Minimum Redundancy Maximum Relevance
tkNN	t Algorithm-Based k-Nearest-Neighbour
LOSO	Leave-One-Subject-Out
XAI	Explainable Artificial Intelligence
AUC	Area Under the Receiver Operating Characteristic Curve
SD	Standard Deviation
AT	Alpha-to-Theta
GA	Gamma-to-Alpha
TB	Theta-to-Beta
BG	Beta-to-Gamma
FL	Left Frontal
FR	Right Frontal
TL	Left Temporal
TR	Right Temporal
PL	Left Parietal
PR	Right Parietal
OL	Left Occipital
OR	Right Occipital
CL	Left Central
CR	Right Central
CI	Confidence Interval
CWINCA	Cumulative-Weighted Neighbourhood Component Analysis
CWIChi2	Cumulative-Weighted Chi-Square
CWIReliefF	Cumulative-Weighted ReliefF
DRLobish	Directed Rhythm Lobish
ROC	Receiver Operating Characteristic
IMV	Iterative Majority Voting

## Appendix A

Appendix A reports the inner-accuracy summary for the classifier-nested analysis. Table A1 lists 95 values, and Figure A1 plots them against the pooled outer accuracy. A single tkNN configuration was selected in 41 outer folds, always with cosine distance: k = 9 with inverse-distance weighting (candidate 59) in 35 folds, k = 10 with squared-inverse weighting (candidate 30) in 5 and k = 10 with equal weighting (candidate 90) in 1. An IMV ensemble was selected in the remaining 54 folds. In 50 of these, it pooled the 44 to 57 highest-ranked configurations (candidates 132–145), and in 4 it pooled 5 or 9 configurations (candidates 93 and 97). Figure A2 maps every candidate index to its configuration. The summary does not include fold-wise feature-selection outputs. It is not a full reproducibility record.

**Table A1:** Reported inner LOSO accuracy (%) for each outer fold of the fixed-panel classifier-nested analysis. Fold numbers identify held-out participants; selected candidate indices are summarised in the preceding paragraph.

Fold	Inner	Fold	Inner	Fold	Inner	Fold	Inner	Fold	Inner
1	94.06	20	93.98	39	94.10	58	93.86	77	93.78
2	93.86	21	93.90	40	94.22	59	93.78	78	94.26
3	94.14	22	94.02	41	94.02	60	93.86	79	94.00
4	94.06	23	94.02	42	93.98	61	94.18	80	94.14
5	93.94	24	94.02	43	93.90	62	93.98	81	94.30
6	94.02	25	93.98	44	94.02	63	93.90	82	94.14
7	94.02	26	94.26	45	93.98	64	94.41	83	93.86
8	94.22	27	94.30	46	94.26	65	93.78	84	93.94
9	93.90	28	93.94	47	94.06	66	93.98	85	93.78
10	94.02	29	93.94	48	94.02	67	94.12	86	93.63
11	94.06	30	93.86	49	94.02	68	93.71	87	94.70
12	94.06	31	94.30	50	94.06	69	93.90	88	93.83
13	94.26	32	93.94	51	93.98	70	93.94	89	93.94
14	94.02	33	93.90	52	94.87	71	93.88	90	94.06
15	93.98	34	94.10	53	93.95	72	94.26	91	93.94
16	93.94	35	94.66	54	93.94	73	94.08	92	94.54
17	94.06	36	94.02	55	93.87	74	93.98	93	93.94
18	94.34	37	94.02	56	94.10	75	94.22	94	93.82
19	94.46	38	94.46	57	94.02	76	93.94	95	94.06

The inner search can also be examined candidate by candidate. Figure A2 shows the inner LOSO accuracy of all 178 candidates for the outer fold in which participant 95 was held out, so that 94 participants and 2526 epochs formed the development set. Complete candidate-level results were stored only for this fold; for the other 94 outer folds, the run log kept the selected candidate and its inner accuracy (Table A1). Panel (a) arranges the 90 tkNN configurations by distance metric, weighting rule and neighbour count. Panel (b) shows the 88 IMV ensembles in order of size, from the 3 to the 90 highest-ranked configurations.

Within this fold, distance metric had the largest effect (Figure A2a). Cosine configurations reached 90.58–93.98%, compared with 84.28–89.83% for city-block and 82.66–86.98% for Euclidean distance. Accuracy also rose with neighbour count, by 3.61 percentage points on average from k = 1 to k = 10, whereas the three weighting rules differed by only 0.15 points on average at a given metric and neighbour count. The best single configuration combined cosine distance, inverse-distance weighting and k = 9 (93.98%). In Figure A2b, ensembles of the 3 to 57 highest-ranked configurations stayed between 93.27% and 94.06%. The ensemble of 47 configurations was best and was the candidate selected for this outer fold. Adding weaker configurations beyond 57 lowered accuracy steadily, to 90.30% when all 90 were pooled. Selections across the 95 outer folds came from the same high-accuracy region, as summarised at the start of this appendix. These inner accuracies describe model selection in a single fold and are not outer-test performance, which is reported in Table 3 and Figure 2 and Figure 3.

Figure A3 shows the confusion matrices of the nested SVM (Section 2.2.7). All 51 controls were classified correctly, and 42 of 44 recorded-offence participants were classified correctly.

## Appendix B

Figure A4 shows the leakage simulation described in Section 2.2.6. The same simulated matrices were passed to the three evaluation schemes, so the bars differ only in where feature selection took place.

On pure noise, the initial classifier-selection procedure reached 96.63–97.49% epoch accuracy and the fixed-panel scheme 95.81–97.38% (participants 97.89–100.00%). The fully nested scheme returned 43.62–53.21% (participants 44.21–56.84%), at or below chance; values below 50% are common for leave-one-out designs on null data. With a true group effect, the fully nested scheme reached 84.53% epoch and 93.68% participant accuracy. Cohort-wide selection can therefore produce accuracies of the size reported in Section 3.2 without any signal, and a fully nested scheme removes this effect while still recovering a real signal (Figure A4).

## Appendix C

Without value weights, each sample adds one permutation matrix per lag, so all row and column sums of the count matrix are equal. A numerical check on a 3840 × 32 random-walk map gave the same sum for every row and column (15,345), and the marginal-product expected matrix was constant. The marginal-contrast step therefore lowers every entry above α/1024 by the same amount (α^2^/1024) and scales smaller entries by 1 − α; diagonal and off-diagonal entries are treated alike. Channel ranks were identical with and without row-wise robust standardisation.

At 256 Hz, lags of 1, 2, 4 and 8 samples cover 0.20%, 0.39%, 0.78% and 1.56% of a cycle at 0.5 Hz, and 1.56%, 3.13%, 6.25% and 12.5% at 4 Hz. For theta (4–8 Hz), the longest lag covers 12.5–25% of a cycle; for gamma (30–80 Hz), it covers 0.94–2.5 cycles. Ranking uses Hilbert envelopes, which vary more slowly than the carrier, so these fractions are an upper bound on the change seen by the ranking. Slow-band layers are therefore expected to be dominated by rank persistence.

The same checks were performed on two raw EEG epochs, one from each group (3840 × 32 samples; 15 s at 256 Hz). An equivalent Python implementation of RhythmFluxPat.m was used. In both epochs, channel ranks were identical with and without row-wise robust standardisation in all nine maps. Every unweighted count matrix had equal row and column sums at all four lags (3839 at lag 1). Each of the nine layers summed to one. The two feature vectors were then located in the saved panels. Min–max scaling keeps the sign of any between-epoch differences. One row pair matched this sign for all 876 coordinates selected by the four selectors (876/876; Figure A5). The next-best pair matched 85.39%. The matched rows belonged to the expected participants, and their positions within the participant blocks matched the epoch numbers of the two files. The released extractor therefore reproduces the stored coordinates for these two epochs. This check covers two epochs only. It is not a cohort-wide re-extraction.

## Appendix D. Independent Validation of the Saved Held-Out Predictions

The saved MATLAB prediction export (nested_loso_cv.mat) contains the true labels (actual_labels), the held-out predictions (tahmin_loso) and the participant indices (indices) for the 2554 EEG epochs. An independent Python script calculated the confusion matrices and performance metrics from these saved predictions without fitting any model. The exported predictions correspond to classifier-nested LOSO on the cohort-selected 352-feature panel. They do not document a fully nested re-ranking or prefix search within every outer training fold.

Epoch-level matrix (actual rows; predicted columns): controls 1362 | 68; recorded-offence 94 | 1030. Participant-level majority-vote matrix: controls 51 | 0; recorded-offence 2 | 42. The participant errors correspond to IDs 53 and 57. Epoch accuracy was 2392/2554 (93.66%), and participant accuracy was 93/95 (97.89%).

Participant-level Wilson 95% intervals: accuracy 92.65–99.42%; recorded-offence sensitivity 84.87–98.74%; control specificity 93.00–100.00%. The intervals quantify binomial uncertainty in the 95 participant decisions conditional on the saved predictions; they do not correct cohort-wide supervised feature selection or prove transportability.

Supplementary Code S1 provides the verification script; running it on the prediction file writes the 95-participant voting table. The raw EEG feature matrix and the fold-wise feature-selection logs are not part of this export, so the export cannot show full-pipeline nesting.

The comparative feature-selection results were independently verified. An independent script recomputed every value in Table 5 and Table 6 from the saved selection indices, panel matrices, held-out predictions and IMV outputs. All 64 consistency checks passed. Each intersection panel contained exactly the shared columns of its parent panels. An independent Python implementation of the 178-candidate tkNN search matched each saved prediction vector on 98.79–99.84% of epochs. On the CWImRMR panel, the matching setting (cosine distance, inverse-distance weights, k = 9) gave 2401/2554 correct epochs in Python and 2397/2554 in MATLAB. Small implementation details can therefore move a branch accuracy by a few epochs. The comparative analysis uses one cohort-wide selection per selector. It therefore does not document nested feature selection.
