# Supplementary material for: Explainable Rhythm-Resolved EEG for Retrospective Comparison of Psychiatric-Service Adults with Recorded Violent Offences and Non-Clinical Controls
Source: Diagnostics (Basel). 2026 Oct 30;16(21):3585. doi: 10.3390/diagnostics16213585 (PMC13761518; doi:10.3390/diagnostics16213585)
Supplement: Supplementary file 1 [file diagnostics-16-03585-s001.zip › diagnostics-4619618-supplementary.pdf]

Supplementary Table S1. Per-participant classification results for the nested support-vector machine (SVM; one outer fold per participant) and the CWImRMR-based tkNN evaluation (Table 5). Both analyses used the fixed 352-feature panel selected using full-cohort class labels. Participant-level predictions were determined by majority voting according to the MATLAB mode rule. In the event of a 50:50 tie, the control class was assigned, and the result was marked as "tie".

ID	Group	Epochs	tkNN: correct epochs	tkNN: vote	SVM: selected setting	SVM: inner accuracy (%)	SVM: correct epochs	SVM: vote
1	Control	28	28	Control	Linear, C = 0.001	94.26	27	Control
2	Control	28	28	Control	Linear, C = 0.001	93.94	28	Control
3	Control	28	26	Control	Linear, C = 0.001	94.22	27	Control
4	Control	28	28	Control	Linear, C = 0.001	94.81	15	Control
5	Control	28	28	Control	Linear, C = 0.001	94.38	28	Control
6	Control	28	28	Control	Linear, C = 0.001	94.10	28	Control
7	Control	28	28	Control	Linear, C = 0.001	94.34	28	Control
8	Control	28	27	Control	Linear, C = 0.1	94.97	20	Control
9	Control	28	28	Control	Linear, C = 0.001	94.02	28	Control
10	Control	28	28	Control	Linear, C = 0.001	94.10	28	Control
11	Control	28	26	Control	Linear, C = 0.001	94.14	28	Control
12	Control	28	26	Control	Linear, C = 0.001	94.42	26	Control
13	Control	28	21	Control	Linear, C = 0.001	94.14	26	Control
14	Control	28	27	Control	Linear, C = 0.001	93.90	28	Control
15	Control	28	28	Control	Linear, C = 0.001	93.98	28	Control
16	Control	28	28	Control	RBF, C = 1, γ = 1/p	94.06	28	Control
17	Control	28	28	Control	RBF, C = 1, γ = 1/p	93.94	28	Control
18	Control	28	21	Control	Linear, C = 0.001	94.58	22	Control
19	Control	28	17	Control	Linear, C = 0.001	94.14	25	Control
20	Control	28	28	Control	Linear, C = 0.001	93.98	28	Control
21	Control	30	30	Control	RBF, C = 1, γ = 0.25/p	94.10	30	Control
22	Control	28	28	Control	Linear, C = 0.001	94.14	28	Control
23	Control	28	28	Control	Linear, C = 0.001	94.14	28	Control
24	Control	28	28	Control	Linear, C = 0.001	94.42	27	Control
25	Control	28	28	Control	Linear, C = 0.001	93.98	28	Control
26	Control	28	23	Control	Linear, C = 0.001	94.54	20	Control
27	Control	28	27	Control	Linear, C = 0.001	94.42	28	Control
28	Control	28	28	Control	Linear, C = 0.001	94.46	28	Control
29	Control	28	28	Control	Linear, C = 0.001	94.66	28	Control
30	Control	28	28	Control	Linear, C = 0.001	94.62	28	Control
31	Control	28	24	Control	Linear, C = 0.001	94.77	27	Control
32	Control	28	28	Control	Linear, C = 0.001	94.34	28	Control
33	Control	28	28	Control	Linear, C = 0.001	94.38	28	Control
34	Control	28	26	Control	Linear, C = 0.001	94.46	28	Control
35	Control	28	14	Control (tie)	Linear, C = 0.001	94.70	20	Control
36	Control	28	28	Control	Linear, C = 0.001	94.22	28	Control
37	Control	28	28	Control	Linear, C = 0.001	94.42	28	Control
38	Control	28	26	Control	Linear, C = 0.001	94.42	28	Control

ID	Group	Epochs	tkNN: correct epochs	tkNN: vote	SVM: selected setting	SVM: inner accuracy (%)	SVM: correct epochs	SVM: vote
39	Control	28	28	Control	Linear, C = 0.001	94.18	28	Control
40	Control	28	24	Control	Linear, C = 0.001	94.30	28	Control
41	Control	28	28	Control	Linear, C = 0.001	94.42	28	Control
42	Control	28	26	Control	Linear, C = 0.001	94.22	28	Control
43	Control	28	28	Control	Linear, C = 0.001	94.30	28	Control
44	Control	28	28	Control	Linear, C = 0.001	94.14	28	Control
45	Control	28	28	Control	Linear, C = 0.001	94.22	28	Control
46	Control	28	26	Control	Linear, C = 0.001	94.26	27	Control
47	Control	28	28	Control	Linear, C = 0.001	94.26	28	Control
48	Control	28	28	Control	Linear, C = 0.001	94.38	28	Control
49	Control	28	28	Control	Linear, C = 0.001	94.18	28	Control
50	Control	28	28	Control	Linear, C = 0.001	94.26	28	Control
51	Control	28	28	Control	Linear, C = 0.001	94.26	28	Control
52	Recorded offence	19	19	Recorded offence	Linear, C = 0.001	94.16	19	Recorded offence
53	Recorded offence	8	8	Recorded offence	Linear, C = 0.001	93.79	8	Recorded offence
54	Recorded offence	28	27	Recorded offence	Linear, C = 0.001	94.26	23	Recorded offence
55	Recorded offence	27	24	Recorded offence	Linear, C = 0.001	93.71	26	Recorded offence
56	Recorded offence	28	28	Recorded offence	Linear, C = 0.001	93.86	28	Recorded offence
57	Recorded offence	28	28	Recorded offence	RBF, C = 1, γ = 0.25/p	93.94	28	Recorded offence
58	Recorded offence	28	28	Recorded offence	RBF, C = 1, γ = 0.25/p	94.10	28	Recorded offence
59	Recorded offence	28	28	Recorded offence	Linear, C = 0.001	94.06	28	Recorded offence
60	Recorded offence	28	28	Recorded offence	RBF, C = 1, γ = 0.25/p	94.14	28	Recorded offence
61	Recorded offence	28	16	Recorded offence	RBF, C = 1, γ = 0.25/p	95.17	19	Recorded offence
62	Recorded offence	28	28	Recorded offence	Linear, C = 0.001	94.85	28	Recorded offence
63	Recorded offence	28	27	Recorded offence	Linear, C = 0.001	94.73	28	Recorded offence
64	Recorded offence	16	8	Control (tie) ✗	RBF, C = 0.1, γ = 0.25/p	93.93	6	Control ✗
65	Recorded offence	28	28	Recorded offence	Linear, C = 0.001	94.81	28	Recorded offence
66	Recorded offence	28	27	Recorded offence	Linear, C = 0.001	94.89	28	Recorded offence
67	Recorded offence	18	13	Recorded offence	Linear, C = 0.001	94.12	17	Recorded offence
68	Recorded offence	28	27	Recorded offence	Linear, C = 0.001	94.73	18	Recorded offence
69	Recorded offence	28	28	Recorded offence	Linear, C = 0.001	93.90	28	Recorded offence
70	Recorded offence	12	12	Recorded offence	Linear, C = 0.001	93.75	11	Recorded offence

ID	Group	Epochs	tkNN: correct epochs	tkNN: vote	SVM: selected setting	SVM: inner accuracy (%)	SVM: correct epochs	SVM: vote
71	Recorded offence	7	7	Recorded offence	Linear, C = 0.001	93.76	7	Recorded offence
72	Recorded offence	28	22	Recorded offence	Linear, C = 0.001	93.75	28	Recorded offence
73	Recorded offence	20	19	Recorded offence	Linear, C = 0.001	94.20	17	Recorded offence
74	Recorded offence	28	28	Recorded offence	Linear, C = 0.001	93.63	27	Recorded offence
75	Recorded offence	28	24	Recorded offence	Linear, C = 0.001	94.30	18	Recorded offence
76	Recorded offence	28	26	Recorded offence	RBF, C = 1, γ = 0.25/p	93.35	26	Recorded offence
77	Recorded offence	28	28	Recorded offence	Linear, C = 0.001	93.43	28	Recorded offence
78	Recorded offence	28	24	Recorded offence	Linear, C = 0.1	93.35	28	Recorded offence
79	Recorded offence	20	20	Recorded offence	Linear, C = 0.001	93.73	20	Recorded offence
80	Recorded offence	28	20	Recorded offence	RBF, C = 10, γ = 1/p	93.78	13	Control ✗
81	Recorded offence	28	22	Recorded offence	Linear, C = 0.001	93.75	19	Recorded offence
82	Recorded offence	28	26	Recorded offence	RBF, C = 1, γ = 0.25/p	93.31	28	Recorded offence
83	Recorded offence	28	28	Recorded offence	Linear, C = 0.001	93.55	28	Recorded offence
84	Recorded offence	28	25	Recorded offence	RBF, C = 1, γ = 0.25/p	93.47	28	Recorded offence
85	Recorded offence	28	28	Recorded offence	RBF, C = 1, γ = 1/p	93.43	28	Recorded offence
86	Recorded offence	28	28	Recorded offence	Linear, C = 0.001	93.75	28	Recorded offence
87	Recorded offence	26	26	Recorded offence	Linear, C = 0.01	93.75	26	Recorded offence
88	Recorded offence	27	27	Recorded offence	Linear, C = 0.001	93.87	27	Recorded offence
89	Recorded offence	28	28	Recorded offence	RBF, C = 1, γ = 0.25/p	93.71	28	Recorded offence
90	Recorded offence	28	26	Recorded offence	Linear, C = 0.001	93.63	28	Recorded offence
91	Recorded offence	28	28	Recorded offence	Linear, C = 0.01	93.90	27	Recorded offence
92	Recorded offence	28	11	Control ✗	Linear, C = 0.001	93.78	26	Recorded offence
93	Recorded offence	28	28	Recorded offence	Linear, C = 0.001	93.71	28	Recorded offence
94	Recorded offence	28	28	Recorded offence	Linear, C = 0.01	93.82	28	Recorded offence
95	Recorded offence	28	27	Recorded offence	Linear, C = 0.001	93.67	28	Recorded offence

Totals: The CWImRMR-based tkNN evaluation correctly classified 2,397/2,554 epochs and 93/95 participants. The nested SVM correctly classified 2,419/2,554 epochs and 93/95 participants. The symbol X indicates an incorrect participant-level prediction.

Supplementary Code S1

4.1. MATLAB/RhythmFluxPat.m

RhythmFluxPat feature extractor (Section 2.2.1, Algorithm 1)

```
function vek = RhythmFluxPat(sinyal)


    epsi = 1e-10;

    %% Size control
    [nSamples, nCh] = size(sinyal);

    if nSamples < nCh
        sinyal = sinyal';
        [nSamples, nCh] = size(sinyal);
    end

    if nSamples <= 128
        error('Signal length is too short for RhythmFluxPat_C9.');
    end

    sinyal = double(sinyal);
    sinyal(~isfinite(sinyal)) = 0;

    %% Automatic sampling frequency
    segmentDurationSec = 15;
    fs = nSamples / segmentDurationSec;

    %% Original robust global normalization
    sinyal = robust_global_z_RhythmFluxPat_C9(sinyal, epsi);

    %% Required EEG bands
    gammaHi = min(80, 0.45 * fs);

    DELTA = bpf_RhythmFluxPat_C9(sinyal, fs, 0.5, 4);
    THETA = bpf_RhythmFluxPat_C9(sinyal, fs, 4,   8);
    ALPHA = bpf_RhythmFluxPat_C9(sinyal, fs, 8,   13);
    BETA  = bpf_RhythmFluxPat_C9(sinyal, fs, 13,  30);
    GAMMA = bpf_RhythmFluxPat_C9(sinyal, fs, 30,  gammaHi);
```

```
%% Original analytic representation
DELTA_H = hilbert(DELTA);
THETA_H = hilbert(THETA);
ALPHA_H = hilbert(ALPHA);
BETA_H  = hilbert(BETA);
GAMMA_H = hilbert(GAMMA);

DELTA_ENV = abs(DELTA_H);
THETA_ENV = abs(THETA_H);
ALPHA_ENV = abs(ALPHA_H);
BETA_ENV  = abs(BETA_H);
GAMMA_ENV = abs(GAMMA_H);

THETA_PHI = angle(THETA_H);
ALPHA_PHI = angle(ALPHA_H);
BETA_PHI  = angle(BETA_H);
GAMMA_PHI = angle(GAMMA_H);

DELTA_ENV(~isfinite(DELTA_ENV)) = 0;
THETA_ENV(~isfinite(THETA_ENV)) = 0;
ALPHA_ENV(~isfinite(ALPHA_ENV)) = 0;
BETA_ENV(~isfinite(BETA_ENV))   = 0;
GAMMA_ENV(~isfinite(GAMMA_ENV)) = 0;

%% Original transition settings
lags = [1, 2, 4, 8];
lagW = [0.50, 0.25, 0.15, 0.10];

if nSamples <= max(lags) + 64
   error('Signal length is too short after lag control for RhythmFluxPat_C9.');
end

%% Layer allocation
nLayers = 9;
mats = zeros(nCh, nCh, nLayers);

%% Layers 1-5: retained single-band transition tables
mats(:,:,1) = make_transition_table_RhythmFluxPat_C9( ...
   ALPHA_ENV, nCh, lags, lagW, 0.12, epsi);
mats(:,:,2) = make_transition_table_RhythmFluxPat_C9( ...
   BETA_ENV, nCh, lags, lagW, 0.12, epsi);
mats(:,:,3) = make_transition_table_RhythmFluxPat_C9( ...
   GAMMA_ENV, nCh, lags, lagW, 0.12, epsi);
mats(:,:,4) = make_transition_table_RhythmFluxPat_C9( ...
```

```
        THETA_ENV, nCh, lags, lagW, 0.12, epsi);
    mats(:,:,5) = make_transition_table_RhythmFluxPat_C9( ...
        DELTA_ENV, nCh, lags, lagW, 0.12, epsi);

    %% Layers 6-9: retained directional cross-rhythm maps
    AT = directed_wave_map_RhythmFluxPat_C9( ...
        ALPHA_ENV, THETA_ENV, ALPHA_PHI, THETA_PHI, epsi);
    GA = directed_wave_map_RhythmFluxPat_C9( ...
        GAMMA_ENV, ALPHA_ENV, GAMMA_PHI, ALPHA_PHI, epsi);
    TB = directed_wave_map_RhythmFluxPat_C9( ...
        THETA_ENV, BETA_ENV, THETA_PHI, BETA_PHI, epsi);
    BG = directed_wave_map_RhythmFluxPat_C9( ...
        BETA_ENV, GAMMA_ENV, BETA_PHI, GAMMA_PHI, epsi);

    mats(:,:,6) = make_transition_table_RhythmFluxPat_C9( ...
        AT, nCh, lags, lagW, 0.14, epsi);
    mats(:,:,7) = make_transition_table_RhythmFluxPat_C9( ...
        GA, nCh, lags, lagW, 0.14, epsi);
    mats(:,:,8) = make_transition_table_RhythmFluxPat_C9( ...
        TB, nCh, lags, lagW, 0.14, epsi);
    mats(:,:,9) = make_transition_table_RhythmFluxPat_C9( ...
        BG, nCh, lags, lagW, 0.14, epsi);

    %% Final normalization and vectorization
    mats(~isfinite(mats)) = 0;

    for k = 1:nLayers
        currentMatrix = mats(:,:,k);
        currentMatrix(~isfinite(currentMatrix)) = 0;

        matrixSum = sum(currentMatrix(:));
        if matrixSum > 0
            currentMatrix = currentMatrix / matrixSum;
        end

        mats(:,:,k) = currentMatrix;
    end

    vek = mats(:)';
    vek(~isfinite(vek)) = 0;

end

function X = robust_global_z_RhythmFluxPat_C9(X, epsi)
```

```
    centerValue = median(X(:));
    madValue = median(abs(X(:) - centerValue));

    X = (X - centerValue) / (1.4826 * madValue + epsi);
    X(~isfinite(X)) = 0;

end


function Y = bpf_RhythmFluxPat_C9(X, fs, lo, hi)

    filterOrder = 4;
    nyquistFrequency = fs / 2;

    lowNormalized = max(lo / nyquistFrequency, 1e-4);
    highNormalized = min(hi / nyquistFrequency, 0.999);

    if highNormalized <= lowNormalized
        Y = zeros(size(X));
        return;
    end

    try
        [b, a] = butter(filterOrder, ...
            [lowNormalized, highNormalized], 'bandpass');
        Y = filtfilt(b, a, X);
    catch
        Y = X;
    end

    Y(~isfinite(Y)) = 0;

end


function Z = zmap_RhythmFluxPat_C9(M, epsi)

    meanValue = mean(M(:));
    standardDeviation = std(M(:));

    Z = (M - meanValue) / (standardDeviation + epsi);
    Z(~isfinite(Z)) = 0;

end
```

```
function Z = row_robust_z_RhythmFluxPat_C9(M, epsi)

    rowMedian = median(M, 2);
    centeredMatrix = M - rowMedian;

    rowScale = median(abs(centeredMatrix), 2);
    rowScale = 1.4826 * rowScale + epsi;

    Z = centeredMatrix ./ rowScale;
    Z(~isfinite(Z)) = 0;

end


function C = directed_wave_map_RhythmFluxPat_C9( ...
    sourceEnvelope, targetEnvelope, sourcePhase, targetPhase, epsi)

    targetEnvelopeLead = [ ...
        targetEnvelope(2:end,:); targetEnvelope(end,:)];
    targetPhaseLead = [targetPhase(2:end,:); targetPhase(end,:)];

    amplitudeMap = sqrt(abs( ...
        sourceEnvelope .* targetEnvelopeLead) + epsi);
    phaseMap = cos(sourcePhase - targetPhaseLead);

    sourceZ = row_robust_z_RhythmFluxPat_C9( ...
        sourceEnvelope, epsi);
    targetZ = row_robust_z_RhythmFluxPat_C9( ...
        targetEnvelopeLead, epsi);

    directionMap = tanh(targetZ - sourceZ);

    C = amplitudeMap .* phaseMap .* (1 + 0.25 * directionMap);
    C = zmap_RhythmFluxPat_C9(C, epsi);
    C(~isfinite(C)) = 0;

end


function M = make_transition_table_RhythmFluxPat_C9( ...
    map, nCh, lags, lagW, contrastAlpha, epsi)

    map(~isfinite(map)) = 0;
    map = row_robust_z_RhythmFluxPat_C9(map, epsi);
```

```
    rankOrder = rank_order_RhythmFluxPat_C9(map);
    M = zeros(nCh, nCh);

    for q = 1:numel(lags)
        M = M + lagW(q) * ...
            temporal_transition_RhythmFluxPat_C9( ...
            rankOrder, map, nCh, lags(q));
    end

    M = transition_contrast_RhythmFluxPat_C9( ...
        M, contrastAlpha, epsi);

    matrixSum = sum(M(:));
    if matrixSum > 0
        M = M / matrixSum;
    end

    M(~isfinite(M)) = 0;

end


function rankOrder = rank_order_RhythmFluxPat_C9(M)

    [nRows, nCh] = size(M);
    rankOrder = zeros(nRows, nCh);
    tieKey = 1e-12 * (1:nCh);

    for t = 1:nRows
        [~, order] = sort(M(t,:) + tieKey, 'descend');
        rankOrder(t,:) = order;
    end

end


function M = temporal_transition_RhythmFluxPat_C9( ...
    rankOrder, map, nCh, lagValue)

    if size(rankOrder,1) <= lagValue
        M = zeros(nCh, nCh);
        return;
    end
```

```
    rankCurrent = rankOrder(1:end-lagValue,:);
    rankFuture = rankOrder(1+lagValue:end,:);
    mapCurrent = map(1:end-lagValue,:);
    mapFuture = map(1+lagValue:end,:);

    [nRows, nRank] = size(rankCurrent);
    sourceChannel = rankCurrent(:);
    targetChannel = rankFuture(:);
    rowID = repmat((1:nRows)', nRank, 1);

    sourceValue = mapCurrent(sub2ind( ...
        size(mapCurrent), rowID, sourceChannel));
    targetValue = mapFuture(sub2ind( ...
        size(mapFuture), rowID, targetChannel));

    transitionWeight = 1 + 0.25 * tanh( ...
        0.5 * (sourceValue + targetValue));
    transitionWeight(~isfinite(transitionWeight)) = 1;
    transitionWeight(transitionWeight < 0) = 0;

    linearIndex = sub2ind([nCh, nCh], ...
        sourceChannel, targetChannel);

    M = reshape(accumarray( ...
        linearIndex, transitionWeight, [nCh*nCh, 1]), ...
        nCh, nCh);
    M(~isfinite(M)) = 0;

end


function contrastedMatrix = transition_contrast_RhythmFluxPat_C9( ...
    M, alpha, epsi)

    M(~isfinite(M)) = 0;
    matrixSum = sum(M(:));

    if matrixSum <= 0
        contrastedMatrix = M;
        return;
    end

    probability = M / (matrixSum + epsi);
    rowProbability = sum(probability, 2);
    columnProbability = sum(probability, 1);
```

```
    expectedMatrix = rowProbability * columnProbability;
    expectedMatrix = expectedMatrix / ...
        (sum(expectedMatrix(:)) + epsi);

    residualMatrix = probability - alpha * expectedMatrix;
    residualMatrix(residualMatrix < 0) = 0;

    contrastedMatrix = (1 - alpha) * probability + ...
        alpha * residualMatrix;
    contrastedMatrix(~isfinite(contrastedMatrix)) = 0;

end
```

4.2. MATLAB/RUN_RhythmFluxPat_SmokeTest.m

Functional verification of RhythmFluxPat.m

```
% Basic functional verification test. Run in MATLAB with Signal Processing Toolbox.
% This test checks dimensions, finiteness and deterministic vectorization.
rng(123); X = randn(3840,32);
a = RhythmFluxPat(X); b = RhythmFluxPat(X);
assert(isrow(a) && numel(a) == 9*32*32);
assert(all(isfinite(a)));
assert(isequal(a,b));
assert(all(a>=0));
% For nine transition matrices, a nonempty layer sums to one.
L=reshape(a,[32,32,9]);
for k=1:9, s=sum(L(:,:,k),'all'); assert(abs(s-1)<1e-8 || s==0); end
fprintf('RhythmFluxPat functional test passed.\n');
```

4.3. MATLAB/DRLobishIndex.m

Feature-index decoding (Section 2.2.4)

```
function [layerCode, source, target] = DRLobishIndex(index)
% Exactly matches manuscript's fixed 1-based feature vectorization.
channels={'Cz','Fz','Fp1','F7','F3','FC1','C3','FC5','FT9','T7', ...
 'CP5','CP1','P3','P7','PO9','O1','Pz','Oz','O2','O10','P8','P4', ...
 'CP2','CP6','T8','FT10','FC6','C4','FC2','F4','F8','Fp2'};
codes={'A','B','G','T','D','AT','GA','TB','BG'};
assert(index==floor(index) && index>=1 && index<=9216);
layer=floor((index-1)/1024)+1;
k=mod(index-1,1024);
source=channels{mod(k,32)+1};target=channels{floor(k/32)+1};
layerCode=codes{layer};
end
```

4.4. Python/verify_nested_loso.py

Verification of held-out classification predictions (Appendix D)

```
"""Recalculate segment-wise and subject-wise Nested LOSO results from MATLAB.

Usage: python verify_nested_loso.py nested_loso_cv.mat
Requires: numpy, scipy, scikit-learn, statsmodels.
Class coding: 1 = Control, 2 = recorded Offence.
"""
import csv
import sys
from pathlib import Path
from collections import Counter
import numpy as np
from scipy.io import loadmat
from sklearn.metrics import confusion_matrix, matthews_corrcoef
from statsmodels.stats.proportion import proportion_confint


def summary(y, yhat):
    C = confusion_matrix(y, yhat, labels=[1, 2])
    tn, fp, fn, tp = C.ravel()
    metrics = {
        "accuracy": (tn + tp) / C.sum(),
        "offence_sensitivity": tp / (tp + fn),
        "control_specificity": tn / (tn + fp),
        "balanced_accuracy": (tp / (tp + fn) + tn / (tn + fp)) / 2,
        "precision_offence": tp / (tp + fp),
        "negative_predictive_value": tn / (tn + fn),
        "F1_offence": 2 * tp / (2 * tp + fp + fn),
        "MCC": matthews_corrcoef(y, yhat),
    }
    return C, metrics


def run(mat_):
    data = loadmat(mat_)
    true = data["actual_labels"].ravel().astype(int)
    predicted = data["tahmin_loso"].flat[0].ravel().astype(int)
    subjects = data["indices"].ravel().astype(int)
    assert len(true) == len(predicted) == len(subjects)
    assert set(np.unique(true)) == {1, 2}
    assert np.array_equal(true, data["y"].ravel())
    rows, subject_true, subject_prediction = [], [], []
    for subject_id in sorted(np.unique(subjects)):
        mask = subjects == subject_id
```

```
        class_true = np.unique(true[mask])
        if len(class_true) != 1:
            raise ValueError(f"Subject {subject_id} has conflicting true labels.")
        votes_control = int(np.sum(predicted[mask] == 1))
        votes_offence = int(np.sum(predicted[mask] == 2))
        if votes_control == votes_offence:
            raise ValueError(f"Subject {subject_id} has a tied majority vote.")
        majority = 1 if votes_control > votes_offence else 2
        subject_true.append(int(class_true[0]))
        subject_prediction.append(majority)
        rows.append([int(subject_id), int(class_true[0]), int(mask.sum()),
                     votes_control, votes_offence, majority, int(majority == class_true[0])])

    cm_segment, ms = summary(true, predicted)
    cm_subject, mp = summary(subject_true, subject_prediction)
    print("Class 1: Control; Class 2: Offence")
    for label, C, metrics in [("Segment-wise", cm_segment, ms),
                              ("Subject-wise", cm_subject, mp)]:
        print(f"\n{label} confusion matrix (actual rows; predicted columns):")
        print(C)
        for k, v in metrics.items():
            unit = "" if k == "MCC" else "%"
            value = v if k == "MCC" else v * 100
            print(f"  {k}: {value:.4f}{unit}")
    print("\nWilson participant-level 95% confidence intervals:")
    tn, fp, fn, tp = cm_subject.ravel()
    for label, good, total in [("Accuracy", tn + tp, cm_subject.sum()),
                               ("Offence sensitivity", tp, tp + fn),
                               ("Control specificity", tn, tn + fp)]:
        low, high = proportion_confint(good, total, method="wilson")
        print(f"  {label}: {100 * low:.2f}–{100 * high:.2f}%")
    errors = [r[0] for r in rows if r[-1] == 0]
    print("Misclassified subject IDs:", errors)
    save_to = Path(mat_).with_name("verified_nested_loso_subjects.csv")
    with save_to.open('w', newline='', encoding='utf-8') as f:
        w = csv.writer(f)
        w.writerow(['Subject_ID', 'Actual', 'Number_of_segments', 'Control_votes',
                    'Offence_votes', 'Majority_prediction', 'Correct'])
        w.writerows(rows)
    print("Saved:", save_to)


if __name__ == '__main__':
    if len(sys.argv) != 2:
        sys.exit('Usage: python verify_nested_loso.py nested_loso_cv.mat')
```

```
    run(sys.argv[1])
```

4.5. Python/validation/structure_checks.py

Summary-level and structural checks (Sections 2.1 and 3.4, Appendix C, Table 5)

```
"""Summary-level and structural checks (Section 2.1, Section 3.4, Appendix C, Table 5).
Writes results/checks/structure_checks.json."""
import json, os
import numpy as np
from scipy import stats

from rfp import features as F
from rfp.stats import welch_from_summary, wilson, hypergeom_summary

OUT = "results/checks"
os.makedirs(OUT, exist_ok=True)
R = {}

# 1. age and sex from the group summaries
R["age_2dec"] = welch_from_summary(30.51, 5.85, 51, 44.48, 11.33, 44)
R["age_1dec"] = welch_from_summary(30.5, 5.8, 51, 44.5, 11.3, 44)
R["sex_fisher_p"] = float(stats.fisher_exact([[6, 45], [4, 40]])[1])
R["pooled_age_mean"] = (51 * 30.51 + 44 * 44.48) / 95
_m = R["pooled_age_mean"]
R["pooled_age_sd"] = float(np.sqrt((50 * 5.85**2 + 43 * 11.33**2 + 51 * (30.51 - _m)**2 + 44 *
(44.48 - _m)**2) / 94))

# 2. lag as a fraction of one cycle at each band edge
R["lag_cycle_fraction"] = {}
for b, (lo, hi) in F.BANDS.items():
    R["lag_cycle_fraction"][b] = {int(L): {"ms": 1000 * L / F.FS, "at_low_edge": L / F.FS * lo,
                                  "at_high_edge": L / F.FS * hi} for L in F.LAGS}

# 3. structural counts and random-selection expectations for a 352-feature panel
sl, pe = F.self_loop_indices(), F.peripheral_indices()
both = np.union1d(sl, pe)
R["structure"] = {"n_total": 9216, "n_self": int(sl.size), "n_peripheral": int(pe.size),
            "n_24ch": 9 * 24 * 24, "n_24ch_no_self": int(9216 - both.size),
            "n_self_or_peripheral": int(both.size)}
R["random352"] = {"self": hypergeom_summary(9216, sl.size, 352),
            "peripheral": hypergeom_summary(9216, pe.size, 352),
            "self_or_peripheral": hypergeom_summary(9216, both.size, 352)}

# 4. doubly stochastic check of the unweighted rank-transition matrix
rng = np.random.default_rng(7)
```

```
M = rng.normal(size=(3840, 32)).cumsum(0)            # random-walk map
T = np.zeros((32, 32))
order = np.argsort(-M, axis=1, kind="stable")
for L in F.LAGS:
    T += np.bincount((order[:-L] * 32 + order[L:]).ravel(), minlength=1024).reshape(32, 32)
R["doubly_stochastic"] = {"row_sum_range": [float(T.sum(1).min()), float(T.sum(1).max())],
                          "col_sum_range": [float(T.sum(0).min()), float(T.sum(0).max())]}
E = np.outer(T.sum(1), T.sum(0)) / T.sum()
R["doubly_stochastic"]["expected_matrix_is_constant"] = bool(np.allclose(E, E[0, 0]))

# 5. step 6: ranking invariance under positive row-wise affine standardisation
Z = (M - np.median(M, 1, keepdims=True)) / (1.4826 * np.median(np.abs(M - np.median(M, 1, keepdims=True)), 1, keepdims=True))
R["step6_rank_identical"] = bool(np.array_equal(np.argsort(-M, 1, kind="stable"), np.argsort(-Z, 1, kind="stable")))

# 6. Table 5: development-stage maxima with naive and cluster-bounded intervals
tab5 = {"CWINCA": (291, 0.8121), "CWIChi2": (142, 0.8833), "CWImRMR": (352, 0.9401), "CWIReliefF": (210, 0.7694)}
R["table5"] = {}
for k, (nf, acc) in tab5.items():
    c = int(round(acc * 2554))
    R["table5"][k] = {"n_features": nf, "correct": c, "acc": c / 2554,
                      "wilson_epochs": wilson(c, 2554),
                      "wilson_neff95": wilson(acc * 95, 95)}  # ICC = 1 bound: n_eff = participants

# 7. fixed-panel participant-level intervals (reported counts)
R["fixed_panel"] = {"part_ci": wilson(93, 95), "sens_ci": wilson(42, 44), "spec_ci": wilson(51, 51)}

json.dump(R, open(os.path.join(OUT, "structure_checks.json"), "w"), indent=1, default=float)
print(json.dumps(R, indent=1, default=float))
```

4.6. Python/validation/simulate_leakage.py

Leakage simulation (Section 2.2.6, Appendix B)

```
"""Leakage demonstration on simulated data with the study's cohort structure.

Three evaluation schemes are applied to the same simulated matrix:
  initial_selection  : ranking + prefix + tkNN chosen on one LOSO stream (all labels)
  fixed_panel : panel chosen on all labels, tkNN/IMV nested per outer fold
  full_nested : ranking + prefix + tkNN/IMV refitted inside each outer fold

Usage: python simulate_leakage.py --out results/sim --seeds 1 2 3 --signal-seed 11
```

```
(settings used for Appendix B: defaults)
"""
import argparse, json, os, time
import numpy as np

from rfp.simdata import noise_features
from rfp import nested
from rfp.stats import metrics, majority, wilson


def run_one(X, y, g, kw, n_jobs):
    out = {}
    t = time.time()
    h = nested.initial_selection_stream(X, y, g, **kw)
    out["initial_selection_epoch_acc"] = h["acc"]
    out["initial_selection_prefix"] = int(h["prefix"])
    panel = h["order"][:h["prefix"]]
    fp = nested.fixed_panel(X, y, g, panel, n_jobs=n_jobs)
    m = metrics(y, fp["pred"]); yt, pt, _, _ = majority(y, fp["pred"], g, fp["score"])
    out["fixed_panel_epoch_acc"] = m["acc"]; out["fixed_panel_part_acc"] = float((yt == pt).mean())
    fn = nested.full_nested(X, y, g, n_jobs=n_jobs, **kw)
    m = metrics(y, fn["pred"]); yt, pt, _, _ = majority(y, fn["pred"], g, fn["score"])
    out["full_nested_epoch_acc"] = m["acc"]; out["full_nested_part_acc"] = float((yt == pt).mean())
    out["full_nested_part_correct"] = int((yt == pt).sum())
    out["full_nested_part_wilson"] = wilson(int((yt == pt).sum()), yt.size)
    out["full_nested_prefix_median"] = float(np.median([f["prefix"] for f in fn["folds"]]))
    out["seconds"] = time.time() - t
    return out


def main():
    ap = argparse.ArgumentParser()
    ap.add_argument("--out", default="results/sim")
    ap.add_argument("--seeds", type=int, nargs="+", default=[1, 2, 3])
    ap.add_argument("--signal-seed", type=int, default=11)
    ap.add_argument("--icc", type=float, default=0.5)
    ap.add_argument("--d", type=int, default=9216)
    ap.add_argument("--max-feat", type=int, default=200)
    ap.add_argument("--prefix-step", type=int, default=10)
    ap.add_argument("--prefilter", type=int, default=1000)
    ap.add_argument("--n-jobs", type=int, default=-1)
    a = ap.parse_args()
    os.makedirs(a.out, exist_ok=True)
```

```python
    kw = dict(ranker="mrmr", max_feat=a.max_feat, prefix_step=a.prefix_step,
              ranker_kw={"prefilter": a.prefilter})
    res = {"settings": vars(a), "null": [], "signal": None}
    for s in a.seeds:
        X, y, g = noise_features(d=a.d, icc=a.icc, seed=s)
        r = run_one(X, y, g, kw, a.n_jobs); r["seed"] = s
        res["null"].append(r)
        print("null", s, json.dumps(r), flush=True)
        json.dump(res, open(os.path.join(a.out, "sim_results.json"), "w"), indent=1)
    X, y, g = noise_features(d=a.d, icc=a.icc, seed=a.signal_seed, effect=0.5, n_informative=50)
    r = run_one(X, y, g, kw, a.n_jobs); r["seed"] = a.signal_seed
    r["signal"] = "50 informative features, +0.5 SD shift in the recorded-offence group"
    res["signal"] = r
    print("signal", json.dumps(r), flush=True)
    json.dump(res, open(os.path.join(a.out, "sim_results.json"), "w"), indent=1)


if __name__ == "__main__":
    main()
```

4.7. Python/validation/make_figure_b1.py

Figure B1

```python
"""Figure B1 from results/sim/sim_results.json."""
import json, os, sys
import numpy as np
import matplotlib
matplotlib.use("Agg")
import matplotlib.pyplot as plt

plt.rcParams.update({"font.family": "serif", "font.size": 9, "axes.spines.top": False,
                     "axes.spines.right": False, "savefig.dpi": 300})
C_MAIN, C_ALT, C_GREY = "#1f4e79", "#c55a11", "#8c8c8c"


def fig_b1(sim_path, out):
    S = json.load(open(sim_path))
    sets = [(f"Noise {r['seed']}", r) for r in S["null"]] + [("True signal", S["signal"])]
    schemes = [("initial_selection", "Initial_selection stream"), ("fixed_panel", "Fixed panel"),
("full_nested", "Fully nested")]
    cols = [C_GREY, C_ALT, C_MAIN]
    fig, ax = plt.subplots(figsize=(6.3, 2.9))
    w = 0.26
    x = np.arange(len(sets))
    for j, ((k, lab), c) in enumerate(zip(schemes, cols)):
```

```
        ep = [100 * r[f"{k}_epoch_acc"] for _, r in sets]
        ax.bar(x + (j - 1) * w, ep, w, color=c, label=lab)
        for xi, v in zip(x + (j - 1) * w, ep):
            ax.text(xi, min(v / 2, 24), f"{v:.1f}", ha="center", va="center", fontsize=6.5, 
color="white", rotation=90)
        if k != "initial_selection":
            pa = [100 * r[f"{k}_part_acc"] for _, r in sets]
            ax.plot(x + (j - 1) * w, pa, "o", ms=3.5, mfc="white", mec="black", mew=0.8,
                    label="Participant accuracy" if k == "full_nested" else None)
    ax.axhline(50, ls="--", lw=0.8, color="black")
    ax.set_xticks(x, [s for s, _ in sets])
    ax.set_ylim(0, 112)
    ax.set_ylabel("LOSO accuracy (%)")
    ax.legend(frameon=False, ncol=4, fontsize=7, loc="upper center", bbox_to_anchor=(0.5, 
1.13))
    fig.tight_layout()
    fig.savefig(out)
    plt.close(fig)



if __name__ == "__main__":
    src = sys.argv[1] if len(sys.argv) > 1 else "results/sim/sim_results.json"
    os.makedirs("results/figures", exist_ok=True)
    fig_b1(src, "results/figures/fig_B1_leakage_simulation.png")
```

4.8. Python/validation/run_all.py

Fully nested LOSO evaluation: main script

```
"""Fully nested LOSO evaluation of RhythmFluxPat features; writes results/results.json.

Minimal call (feature matrix only):
    python run_all.py --features X_rfp.mat --out results --n-jobs 8

Full call:
    python run_all.py --features X_rfp.mat --ages ages.csv --panel panel352.txt \
        --epochs epochs.mat --x24 X_rfp_24ch.mat --xnostep6 X_rfp_nostep6.mat \
        --out results --n-jobs 8 --perm-n 200

Inputs
  --features  .mat/.npz with X (2554 x 9216 RAW RhythmFluxPat features, i.e. before
            cohort min-max), y (1 = control, 2 = recorded-offence), subj (participant id)
  --ages      CSV with header subj,age (one row per participant, same ids as subj)
  --panel     the 352 CWImRMR indices of the published panel (1-based, rank order)
  --epochs    .mat with E (2554 x 3840 x 32), same row order as X (band-power baselines)
```

```
  --bandpower optional .mat with abs, rel, comb matrices instead of --epochs
  --x24       features from MATLAB/RhythmFluxPat.m run on the 24 non-peripheral channels
  --xnostep6   features from RhythmFluxPat.m with the row-wise robust standardisation
removed
"""
import argparse, json, os, time
import numpy as np

from rfp import io, nested, features as F, stats as S, tknn
from rfp.selectors import RANKERS


def _jsonable(o):
    if isinstance(o, dict):
        return {k: _jsonable(v) for k, v in o.items()}
    if isinstance(o, (list, tuple)):
        return [_jsonable(v) for v in o]
    if isinstance(o, (np.integer,)):
        return int(o)
    if isinstance(o, (np.floating,)):
        return float(o)
    if isinstance(o, np.ndarray):
        return _jsonable(o.tolist())
    return o


def summarise(y, g, run, label):
    pred, score = run["pred"], run["score"]
    ep = S.metrics(y, pred)
    ep["ci"] = S.cluster_bootstrap_acc(y, pred, g)
    yt, pt, ids, ties = S.majority(y, pred, g, score)
    pa = S.metrics(yt, pt)
    pa["ci"] = S.wilson(pa["correct"], pa["n"])
    pa["sens_ci"] = S.wilson(pa["tp"], pa["tp"] + pa["fn"])
    pa["spec_ci"] = S.wilson(pa["tn"], pa["tn"] + pa["fp"])
    pa["ties"] = ties
    out = {"label": label, "epoch": ep, "participant": pa}
    if "folds" in run:
        pref = [f["prefix"] for f in run["folds"]]
        cand = [f["candidate"] for f in run["folds"]]
        inner = [f["inner_acc"] for f in run["folds"]]
        out["prefix_median"] = float(np.median(pref))
        out["prefix_range"] = [int(min(pref)), int(max(pref))]
        out["inner_mean"] = float(np.mean(inner))
        out["candidates"] = {tknn.candidate_name(int(c)): int(n)
```

```
                    for c, n in zip(*np.unique(cand, return_counts=True))}
    return out, yt, pt, ids


def save_run(path, run):
    np.savez_compressed(path, pred=run["pred"], score=run["score"],
                        prefixes=np.array([f["prefix"] for f in run.get("folds", [])]),
                        candidates=np.array([f["candidate"] for f in run.get("folds", [])]))


def main():
    ap = argparse.ArgumentParser(description=__doc__,
formatter_class=argparse.RawDescriptionHelpFormatter)
    ap.add_argument("--features", required=True)
    ap.add_argument("--ages"); ap.add_argument("--panel"); ap.add_argument("--epochs")
    ap.add_argument("--bandpower"); ap.add_argument("--x24"); ap.add_argument("--
xnostep6")
    ap.add_argument("--out", default="results")
    ap.add_argument("--n-jobs", type=int, default=-1)
    ap.add_argument("--max-feat", type=int, default=500)
    ap.add_argument("--prefix-step", type=int, default=1)
    ap.add_argument("--mrmr-prefilter", type=int, default=0, help="0 = rank all 9,216
features")
    ap.add_argument("--perm-n", type=int, default=200)
    ap.add_argument("--perm-prefix-step", type=int, default=10)
    ap.add_argument("--perm-prefilter", type=int, default=2000)
    ap.add_argument("--match-caliper", type=float, default=3.0)
    ap.add_argument("--analyses",
default="main,perm,age,baselines,ablations,selectors,diag")
    ap.add_argument("--seed", type=int, default=2026)
    a = ap.parse_args()
    todo = set(a.analyses.split(","))
    os.makedirs(a.out, exist_ok=True)
    rpath = os.path.join(a.out, "results.json")
    R = json.load(open(rpath)) if os.path.exists(rpath) else {}
    R["settings"] = vars(a)

    def dump():
        json.dump(_jsonable(R), open(rpath, "w"), indent=1)

    X, y, g, g_raw = io.load_features(a.features)
    R["data"] = {"n_epochs": int(y.size), "n_features": int(X.shape[1]),
              "n_participants": int(np.unique(g).size),
              "n_ctrl": int(np.unique(g[y == 1]).size), "n_off": int(np.unique(g[y == 2]).size),
              "epochs_ctrl": int((y == 1).sum()), "epochs_off": int((y == 2).sum())}
```

```
    pre = a.mrmr_prefilter or None
    KW = dict(ranker="mrmr", max_feat=a.max_feat, prefix_step=a.prefix_step,
              ranker_kw={"prefilter": pre})
    print(json.dumps(R["data"]), flush=True)

    def full(Xm, label, key, ranker="mrmr", transform=None, yy=None, gg=None, **extra):
        yy = y if yy is None else yy
        gg = g if gg is None else gg
        kw = dict(KW); kw["ranker"] = ranker
        kw["max_feat"] = min(a.max_feat, Xm.shape[1])
        if ranker != "mrmr":
            kw["ranker_kw"] = {}
        kw.update(extra)
        t = time.time()
        run = nested.full_nested(Xm, yy, gg, n_jobs=a.n_jobs, transform=transform, **kw)
        summ, yt, pt, ids = summarise(yy, gg, run, label)
        summ["seconds"] = time.time() - t
        save_run(os.path.join(a.out, f"pred_{key}.npz"), run)
        print(key, f"epoch {summ['epoch']['acc']:.4f} participant
{summ['participant']['acc']:.4f}", flush=True)
        return run, summ, yt, pt

    store = {}
    # ------------------------------------------------------------------ 1. main
    if "main" in todo or "baselines" in todo or "ablations" in todo:
        run, summ, yt, pt = full(X, "RhythmFluxPat, fully nested", "main")
        R["main"] = summ; store["main"] = (run, yt, pt); dump()
        if a.panel:
            panel = io.load_panel(a.panel)
            sel = [set(f["selected"].tolist()) for f in run["folds"]]
            freq = np.array([np.mean([p in s for s in sel]) for p in panel])
            jac = [len(s & set(panel)) / len(s | set(panel)) for s in sel]
            R["main"]["panel_stability"] = {
                "n_panel": int(panel.size), "n_selected_ge50": int((freq >= 0.5).sum()),
                "n_selected_ge90": int((freq >= 0.9).sum()), "median_freq": float(np.median(freq)),
                "jaccard_median": float(np.median(jac)), "jaccard_range": [float(min(jac)),
float(max(jac))]}
            fp = nested.fixed_panel(X, y, g, panel, n_jobs=a.n_jobs)
            R["fixed_panel_python"], _, _, _ = summarise(y, g, fp, "fixed 352 panel (Python
parity)")
            dump()

    # ------------------------------------------------------------------ 2. permutation
    if "perm" in todo:
```

```
        kw = dict(KW); kw["prefix_step"] = a.perm_prefix_step; kw["ranker_kw"] = {"prefilter":
a.perm_prefilter}
        obs = nested.full_nested(X, y, g, n_jobs=a.n_jobs, **kw)
        o_ep = S.metrics(y, obs["pred"])["acc"]
        yt, pt, _, _ = S.majority(y, obs["pred"], g, obs["score"]); o_pa = float((yt == pt).mean())
        rng = np.random.default_rng(a.seed)
        null_ep, null_pa = [], []
        for i in range(a.perm_n):
            yp = nested.permute_labels(y, g, rng)
            r = nested.full_nested(X, yp, g, n_jobs=a.n_jobs, **kw)
            null_ep.append(S.metrics(yp, r["pred"])["acc"])
            yt, pt, _, _ = S.majority(yp, r["pred"], g, r["score"]); null_pa.append(float((yt ==
pt).mean()))
            if (i + 1) % 10 == 0:
                print(f"perm {i + 1}/{a.perm_n}", flush=True)
                R["perm"] = {"n_done": i + 1}
                dump()
        R["perm"] = {"n": a.perm_n, "settings": kw, "obs_epoch": o_ep, "obs_part": o_pa,
                    "null_epoch": null_ep, "null_part": null_pa,
                    "p_epoch": S.perm_p(o_ep, null_ep), "p_part": S.perm_p(o_pa, null_pa),
                    "null_epoch_mean": float(np.mean(null_ep)), "null_epoch_95":
float(np.percentile(null_ep, 95)),
                    "null_part_mean": float(np.mean(null_pa)), "null_part_95":
float(np.percentile(null_pa, 95))}
        dump()

    # ---------------------------------------------------------------- 3. age
    if "age" in todo and a.ages:
        ages = io.load_ages(a.ages)
        missing = sorted(set(g_raw) - set(ages))
        if missing:
            raise SystemExit(f"ages missing for participants: {missing[:10]} ...")
        age_ep = np.array([ages[s] for s in g_raw])
        subs = np.unique(g)
        age_p = np.array([age_ep[g == s][0] for s in subs]); lab_p = np.array([y[g == s][0] for s in
subs])
        a1, a2 = age_p[lab_p == 1], age_p[lab_p == 2]
        w = S.welch_from_summary(a1.mean(), a1.std(ddof=1), a1.size, a2.mean(),
a2.std(ddof=1), a2.size)
        from scipy.stats import mannwhitneyu, spearmanr
        from sklearn.linear_model import LogisticRegression
        from sklearn.metrics import roc_auc_score
        A = {"ctrl_mean": a1.mean(), "ctrl_sd": a1.std(ddof=1), "off_mean": a2.mean(), "off_sd":
a2.std(ddof=1),
             "welch": w, "mannwhitney_p": float(mannwhitneyu(a1, a2).pvalue)}
```

```
    # age-only LOSO (participant level, logistic regression)
    prob = np.zeros(subs.size)
    for i in range(subs.size):
        tr = np.arange(subs.size) != i
        lr = LogisticRegression().fit(age_p[tr, None], lab_p[tr])
        prob[i] = lr.predict_proba(age_p[i:i + 1, None])[0, list(lr.classes_).index(2)]
    pr = np.where(prob > 0.5, 2, 1)
    m = S.metrics(lab_p, pr)
    m["ci"] = S.wilson(m["correct"], m["n"]); m["auc"] = float(roc_auc_score(lab_p == 2,
prob))
    A["age_only_logistic"] = m
    # age-only through the same tkNN/IMV nested engine (epoch level)
    run, summ, _, _ = full(age_ep[:, None], "age only, tkNN nested", "age_only",
fixed_order=[0])
    A["age_only_tknn"] = summ
    # age-residualised EEG, fully nested (residualisation fitted on outer-training epochs)
    run, summ, _, _ = full(X, "RhythmFluxPat, age-residualised", "age_resid",
                    transform=nested.age_residualiser(age_ep))
    A["age_residualised"] = summ
    # does the fully nested EEG score track age within groups?
    if "main" in store:
        sc = store["main"][0]["score"]
        ms = np.array([sc[g == s].mean() for s in subs])
        A["score_age_rho_ctrl"] = spearmanr(ms[lab_p == 1], a1)
        A["score_age_rho_off"] = spearmanr(ms[lab_p == 2], a2)
        A["score_age_rho_ctrl"] = [float(A["score_age_rho_ctrl"][0]),
float(A["score_age_rho_ctrl"][1])]
        A["score_age_rho_off"] = [float(A["score_age_rho_off"][0]),
float(A["score_age_rho_off"][1])]
    # age-matched subset (greedy nearest neighbour, without replacement, caliper)
    ctrl_ids = list(subs[lab_p == 1]); pairs = []
    order_off = subs[lab_p == 2][np.argsort(age_p[lab_p == 2])]
    for s in order_off:
        sa = age_p[subs == s][0]
        if not ctrl_ids:
            break
        ca = np.array([age_p[subs == c][0] for c in ctrl_ids])
        j = int(np.argmin(np.abs(ca - sa)))
        if abs(ca[j] - sa) <= a.match_caliper:
            pairs.append((s, ctrl_ids.pop(j)))
    keep = np.array([s for p in pairs for s in p])
    A["matched_pairs"] = len(pairs)
    if len(pairs) >= 8:
        mk = np.isin(g, keep)
```

```
        ma1 = np.array([age_p[subs == p[1]][0] for p in pairs]); ma2 = np.array([age_p[subs
== p[0]][0] for p in pairs])
        A["matched_age"] = {"ctrl_mean": ma1.mean(), "ctrl_sd": ma1.std(ddof=1),
                     "off_mean": ma2.mean(), "off_sd": ma2.std(ddof=1),
                     "welch": S.welch_from_summary(ma1.mean(), ma1.std(ddof=1),
ma1.size,
                                   ma2.mean(), ma2.std(ddof=1), ma2.size),
                     "range": [float(min(ma1.min(), ma2.min())), float(max(ma1.max(),
ma2.max()))]}
        run, summ, _, _ = full(X[mk], "RhythmFluxPat, age-matched subset", "age_matched",
                      yy=y[mk], gg=g[mk])
        A["age_matched"] = summ
      R["age"] = A
      dump()

  # ---------------------------------------------------------------- 4. baselines
  if "baselines" in todo and (a.epochs or a.bandpower):
      if a.bandpower:
        d = io.load_any(a.bandpower); BP = {k: np.asarray(d[k], float) for k in ("abs", "rel",
"comb")}
      else:
        BP = F.band_power(io.load_epochs(a.epochs))
      B = {}
      main_run, myt, mpt = store["main"]
      for k, name in (("abs", "Absolute band power"), ("rel", "Relative band power"),
              ("comb", "Combined band power")):
        run, summ, yt, pt = full(BP[k], name + ", fully nested", f"bp_{k}")
        b, c, p = S.mcnemar_exact(mpt == myt, pt == yt)
        diff, ci = S.cluster_bootstrap_diff(y, main_run["pred"], run["pred"], g)
        summ["vs_rfp"] = {"mcnemar_rfp_only": b, "mcnemar_base_only": c, "mcnemar_p":
p,
                    "epoch_diff": diff, "epoch_diff_ci": ci}
        B[k] = summ
        R["baselines"] = B; dump()

  # ---------------------------------------------------------------- 5. ablations
  if "ablations" in todo:
      Ab = {}
      sl, pe = F.self_loop_indices(), F.peripheral_indices()
      dlt = F.layer_indices("D")
      allf = np.arange(X.shape[1])
      sets = {"no_self": np.setdiff1d(allf, sl), "no_peripheral": np.setdiff1d(allf, pe),
          "no_self_no_peripheral": np.setdiff1d(allf, np.union1d(sl, pe)),
          "no_delta_layer": np.setdiff1d(allf, dlt)}
      main_run, myt, mpt = store["main"]
```

```
        for key, cols in sets.items():
            run, summ, yt, pt = full(X[:, cols], key, f"abl_{key}")
            summ["n_features"] = int(cols.size)
            diff, ci = S.cluster_bootstrap_diff(y, main_run["pred"], run["pred"], g)
            summ["vs_rfp"] = {"epoch_diff": diff, "epoch_diff_ci": ci,
                              "mcnemar_p": S.mcnemar_exact(mpt == myt, pt == yt)[2]}
            Ab[key] = summ; R["ablations"] = Ab; dump()
        if a.x24:
            X24, y24, g24, _ = io.load_features(a.x24)
            run, summ, yt, pt = full(X24, "24-channel independent extraction", "abl_reextract24")
            summ["n_features"] = int(X24.shape[1])
            diff, ci = S.cluster_bootstrap_diff(y, main_run["pred"], run["pred"], g)
            summ["vs_rfp"] = {"epoch_diff": diff, "epoch_diff_ci": ci, "mcnemar_p":
S.mcnemar_exact(mpt == myt, pt == yt)[2]}
            Ab["reextract_24ch"] = summ
        if a.xnostep6:
            X6, _, _, _ = io.load_features(a.xnostep6)
            dif = np.abs(X6 - X)
            r = [np.corrcoef(X[:, j], X6[:, j])[0, 1] for j in range(X.shape[1]) if X[:, j].std() > 0]
            Ab["step6"] = {"max_abs_diff": float(dif.max()), "mean_abs_diff": float(dif.mean()),
                           "median_feature_r": float(np.nanmedian(r)), "min_feature_r":
float(np.nanmin(r))}
            if dif.max() > 0:
                run, summ, yt, pt = full(X6, "step 6 removed", "abl_nostep6")
                diff, ci = S.cluster_bootstrap_diff(y, main_run["pred"], run["pred"], g)
                summ["vs_rfp"] = {"epoch_diff": diff, "epoch_diff_ci": ci,
                                  "mcnemar_p": S.mcnemar_exact(mpt == myt, pt == yt)[2]}
            else:  # identical features -> identical nested result
                summ = dict(R["main"]); summ["vs_rfp"] = {"epoch_diff": 0.0, "epoch_diff_ci": [0.0,
0.0], "mcnemar_p": 1.0}
            summ["n_features"] = int(X6.shape[1])
            Ab["step6"]["nested"] = summ
        R["ablations"] = Ab; dump()

    # ---------------------------------------------------------------- 6. selectors
    if "selectors" in todo:
        Sel = {"mrmr": R.get("main")}
        for rk in ("chi2", "relieff", "nca"):
            run, summ, _, _ = full(X, f"CWI-{rk}, fully nested", f"sel_{rk}", ranker=rk)
            Sel[rk] = summ; R["selectors"] = Sel; dump()

    # ---------------------------------------------------------------- 7. diagonal mass + composition
    if "diag" in todo:
        Dg = {}
        per_layer_sum = np.stack([X[:, k * 1024:(k + 1) * 1024].sum(1) for k in range(9)], 1)
```

```
        Dg["layer_sum_median"] = float(np.median(per_layer_sum))
        Dg["features_look_raw"] = bool(np.allclose(np.median(per_layer_sum, 0), 1.0, atol=0.05))
        dm = F.diagonal_mass(X)
        Dg["uniform_expectation"] = 1.0 / 32
        Dg["layers"] = {F.LAYERS[k]: {"mean": dm[:, k].mean(), "sd": dm[:, k].std(ddof=1),
                                      "ctrl_mean": dm[y == 1, k].mean(), "off_mean": dm[y == 2, k].mean()}
                        for k in range(9)}
        sl, pe = F.self_loop_indices(), F.peripheral_indices()
        if a.panel:
            panel = io.load_panel(a.panel)
            Dg["panel_self"] = S.hypergeom_summary(9216, sl.size, panel.size, np.isin(panel, sl).sum())
            Dg["panel_peripheral"] = S.hypergeom_summary(9216, pe.size, panel.size, np.isin(panel, pe).sum())
            lay, _, _ = F.decode(panel)
            Dg["panel_delta_layer"] = int((lay == 4).sum())
        if "main" in store:
            fs = store["main"][0]["folds"]
            ns = np.array([np.isin(f["selected"], sl).sum() for f in fs])
            npf = np.array([np.isin(f["selected"], pe).sum() for f in fs])
            sz = np.array([len(f["selected"]) for f in fs])
            Dg["fold_self_mean"] = float(ns.mean()); Dg["fold_self_expected"] = float((sz * sl.size / 9216).mean())
            Dg["fold_peri_mean"] = float(npf.mean()); Dg["fold_peri_expected"] = float((sz * pe.size / 9216).mean())
            Dg["fold_size_mean"] = float(sz.mean())
        R["diag"] = Dg; dump()

    print("done ->", rpath)


if __name__ == "__main__":
    main()
```

4.9. Python/validation/rfp/nested.py

Participant-level LOSO validation procedures

```
"""Leave-one-subject-out evaluation schemes.

initial_selection_stream : ranking, prefix and classifier chosen on the same LOSO stream
                 (development estimate; optimistic).
fixed_panel       : panel fixed in advance; classifier/IMV choice nested in each
                 outer fold (the classifier-nested analysis).
full_nested       : ranking, CWI prefix search, tkNN/IMV choice and any optional
```

```
            fold transform (e.g. age residualisation) are refitted inside
            every outer fold on the 94 training participants only.
"""
import numpy as np
from joblib import Parallel, delayed

from . import tknn
from .selectors import RANKERS, cwi_start


def _standardise(Xtr, Xte):
    mu = Xtr.mean(0)
    sd = Xtr.std(0, ddof=1)
    sd[~np.isfinite(sd) | (sd < 1e-12)] = 1.0
    return (Xtr - mu) / sd, (Xte - mu) / sd


def _prefix_grid(start, max_feat, step):
    start = max(1, min(start, max_feat))
    grid = list(range(start, max_feat + 1, max(1, step)))
    if grid[-1] != max_feat:
        grid.append(max_feat)
    return grid


def fit_fold(Xtr, ytr, gtr, ranker="mrmr", max_feat=500, prefix_step=1, start_mass=0.05,
             ranker_kw=None, fixed_order=None):
    """Everything that uses labels happens here, on training participants only."""
    ranker_kw = ranker_kw or {}
    if fixed_order is not None:
        order = np.asarray(fixed_order)
        grid = [order.size]
        max_feat = order.size
    else:
        max_feat = min(max_feat, Xtr.shape[1])
        if ranker == "mrmr":
            order, scores = RANKERS[ranker](Xtr, ytr, max_feat=max_feat, **ranker_kw)
        else:
            order, scores = RANKERS[ranker](Xtr, ytr, **ranker_kw)
        grid = _prefix_grid(cwi_start(scores, start_mass), max_feat, prefix_step)
    cols = order[:max_feat]
    Ztr, _ = _standardise(Xtr[:, cols], Xtr[:1, cols])
    sc = tknn.inner_scan(Ztr, ytr, gtr, grid)
    cand, cand_acc, rank = tknn.imv_select(sc["acc"], sc["preds"], ytr)
```

```
    return dict(order=cols, prefix=sc["prefix"], candidate=cand,
inner_acc=float(cand_acc[cand - 1]),
                rank=rank, curve=sc["curve"], cand_acc=cand_acc)


def _outer_fold(X, y, g, s, kw, transform):
    tr, te = g != s, g == s
    Xtr, Xte = X[tr], X[te]
    if transform is not None:
        Xtr, Xte = transform(Xtr, Xte, tr, te)
    m = fit_fold(Xtr, y[tr], g[tr], **kw)
    cols = m["order"][:m["prefix"]]
    Ztr, Zte = _standardise(Xtr[:, cols], Xte[:, cols])
    pred, score = tknn.predict(Ztr, y[tr], Zte, m["candidate"], m["rank"])
    return dict(subj=s, idx=np.where(te)[0], pred=pred, score=score, prefix=m["prefix"],
                candidate=m["candidate"], inner_acc=m["inner_acc"], selected=cols,
                cand_acc=m["cand_acc"])


def full_nested(X, y, g, n_jobs=-1, transform=None, verbose=0, **kw):
    subs = np.unique(g)
    folds = Parallel(n_jobs=n_jobs, verbose=verbose)(
        delayed(_outer_fold)(X, y, g, s, kw, transform) for s in subs)
    pred = np.zeros(y.size, np.int8); score = np.zeros(y.size)
    for f in folds:
        pred[f["idx"]] = f["pred"]; score[f["idx"]] = f["score"]
    return dict(pred=pred, score=score, folds=folds)


def fixed_panel(X, y, g, panel_order, n_jobs=-1, verbose=0):
    return full_nested(X, y, g, n_jobs=n_jobs, verbose=verbose, fixed_order=panel_order)


def initial_selection_stream(X, y, g, ranker="mrmr", max_feat=500, prefix_step=1,
start_mass=0.05,
                             ranker_kw=None):
    """Rank on ALL participants, pick prefix and tkNN config on the same LOSO stream."""
    m = fit_fold(X, y, g, ranker=ranker, max_feat=max_feat, prefix_step=prefix_step,
                 start_mass=start_mass, ranker_kw=ranker_kw)
    best_single = int(np.argmax(m["cand_acc"][:tknn.N_SINGLE])) + 1
    return dict(order=m["order"], prefix=m["prefix"], acc=float(m["cand_acc"][best_single -
1]),
                candidate=best_single, curve=m["curve"])
```

```
# ---------------------------------------------------------------- fold transforms
def age_residualiser(age_per_epoch):
    """Regress every feature on age using outer-training epochs only; apply to both."""
    a = np.asarray(age_per_epoch, float)

    def tf(Xtr, Xte, tr, te):
        A = np.c_[np.ones(tr.sum()), a[tr]]
        beta, *_ = np.linalg.lstsq(A, Xtr, rcond=None)
        Rtr = Xtr - A @ beta
        Rte = Xte - np.c_[np.ones(te.sum()), a[te]] @ beta
        return Rtr, Rte
    return tf


def permute_labels(y, g, rng):
    """Shuffle labels at participant level (group sizes and epoch structure preserved)."""
    subs = np.unique(g)
    lab = np.array([y[g == s][0] for s in subs])
    lab = rng.permutation(lab)
    m = dict(zip(subs, lab))
    return np.array([m[s] for s in g])
```

4.10. Python/validation/rfp/selectors.py

Feature ranking methods (mRMR, chi-square, ReliefF, NCA)

```
"""Supervised feature rankers fitted on TRAINING rows only.

Every ranker returns (order, scores): `order` lists feature indices from best to
worst (at least `max_feat` long), `scores` are the ranking scores aligned with
`order`. The cumulative-weighted (CWI) start prefix is computed from these scores.
"""
import numpy as np
from scipy.stats import rankdata, chi2 as chi2_dist


# ---------------------------------------------------------------- discretisation
def quantile_bins(X, nb=10):
    """Equal-frequency bins per column (ties share a bin). Returns int16 codes."""
    n = X.shape[0]
    R = rankdata(X, axis=0, method="average")
    B = np.floor((R - 0.5) / n * nb).astype(np.int16)
    return np.clip(B, 0, nb - 1)


def _mi_from_counts(C, n):
```

```
    """C: (d, a, b) joint counts -> MI (nats) per feature."""
    P = C / n
    pa = P.sum(2, keepdims=True)
    pb = P.sum(1, keepdims=True)
    with np.errstate(divide="ignore", invalid="ignore"):
        t = P * (np.log(P) - np.log(pa) - np.log(pb))
    return np.nansum(t, axis=(1, 2))


def mi_with_vector(B, v, nb, nv):
    """MI between every column of B (codes 0..nb-1) and one discrete vector v (0..nv-1)."""
    n, d = B.shape
    codes = (B.astype(np.int64) * nv + v[:, None]) + (np.arange(d, dtype=np.int64) * nb * nv)[None, :]
    C = np.bincount(codes.ravel(), minlength=d * nb * nv).reshape(d, nb, nv)
    return _mi_from_counts(C.astype(np.float64), n)


# ---------------------------------------------------------------- mRMR (MIQ)
def mrmr(X, y, max_feat=500, nb=10, prefilter=None):
    """Greedy mRMR with the mutual-information quotient (MIQ) criterion,
    as in MATLAB fscmrmr. `prefilter` keeps the top-k features by relevance
    before the greedy search (speed option; None = all features)."""
    B = quantile_bins(X, nb)
    yy = (y - y.min()).astype(np.int64)
    rel = mi_with_vector(B, yy, nb, 2)
    cand = np.arange(X.shape[1])
    if prefilter is not None and prefilter < X.shape[1]:
        cand = np.argsort(-rel, kind="stable")[:prefilter]
    Bc, relc = B[:, cand], rel[cand]
    d = cand.size
    max_feat = min(max_feat, d)
    selected, sel_scores = [], []
    red = np.zeros(d)
    avail = np.ones(d, bool)
    first = int(np.argmax(relc))
    selected.append(first); sel_scores.append(relc[first]); avail[first] = False
    for step in range(1, max_feat):
        last = selected[-1]
        red += mi_with_vector(Bc, Bc[:, last].astype(np.int64), nb, nb)
        score = relc / np.maximum(red / len(selected), 1e-12)
        score[~avail] = -np.inf
        j = int(np.argmax(score))
        selected.append(j); sel_scores.append(score[j]); avail[j] = False
    # remaining features scored with the criterion at the final step
```

```python
    if avail.any():
        last = selected[-1]
        red += mi_with_vector(Bc, Bc[:, last].astype(np.int64), nb, nb)
        rest = np.where(avail)[0]
        rs = relc[rest] / np.maximum(red[rest] / len(selected), 1e-12)
        o = np.argsort(-rs, kind="stable")
        selected += list(rest[o]); sel_scores += list(rs[o])
    order = cand[np.array(selected)]
    scores = np.array(sel_scores, float)
    if cand.size < X.shape[1]:  # unranked tail gets the lowest score
        tail = np.setdiff1d(np.arange(X.shape[1]), cand)
        tail = tail[np.argsort(-rel[tail], kind="stable")]
        order = np.concatenate([order, tail])
        scores = np.concatenate([scores, np.full(tail.size, scores.min())])
    return order, scores


# ------------------------------------------------------------------ chi-square
def chi2_rank(X, y, nb=10, **_):
    B = quantile_bins(X, nb)
    yy = (y - y.min()).astype(np.int64)
    n, d = B.shape
    codes = (B.astype(np.int64) * 2 + yy[:, None]) + (np.arange(d) * nb * 2)[None, :]
    C = np.bincount(codes.ravel(), minlength=d * nb * 2).reshape(d, nb, 2).astype(float)
    E = C.sum(2, keepdims=True) * C.sum(1, keepdims=True) / n
    with np.errstate(divide="ignore", invalid="ignore"):
        stat = np.nansum(np.where(E > 0, (C - E) ** 2 / E, 0.0), axis=(1, 2))
    dof = np.maximum((C.sum(2) > 0).sum(1) - 1, 1)
    logp = chi2_dist.logsf(stat, dof)
    score = -logp
    score[~np.isfinite(score)] = np.nanmax(score[np.isfinite(score)]) + 1
    order = np.argsort(-score, kind="stable")
    return order, score[order]


# ------------------------------------------------------------------ ReliefF
def relieff(X, y, k=10, chunk=128, **_):
    mn, mx = X.min(0), X.max(0)
    Xn = (X - mn) / np.maximum(mx - mn, 1e-12)
    n, d = Xn.shape
    sq = (Xn ** 2).sum(1)
    W = np.zeros(d)
    for s in range(0, n, chunk):
        e = min(n, s + chunk)
        D = sq[s:e, None] + sq[None, :] - 2 * Xn[s:e] @ Xn.T
```

```
        D[np.arange(e - s), np.arange(s, e)] = np.inf
        same = y[None, :] == y[s:e, None]
        Dh = np.where(same, D, np.inf)
        Dm = np.where(~same, D, np.inf)
        kh = min(k, max(1, same.sum(1).min() - 1))
        km = min(k, (~same).sum(1).min())
        H = np.argpartition(Dh, kh, axis=1)[:, :kh]
        M = np.argpartition(Dm, km - 1, axis=1)[:, :km]
        xi = Xn[s:e, None, :]
        W -= np.abs(xi - Xn[H]).mean(1).sum(0)
        W += np.abs(xi - Xn[M]).mean(1).sum(0)
    W /= n
    order = np.argsort(-W, kind="stable")
    return order, W[order]


# ------------------------------------------------------------------ NCA feature weights
def nca_rank(X, y, prefilter=500, n_sub=400, lam=None, sigma=1.0, seed=0, **_):
    """fscnca-style feature weighting (Yang et al. 2012) fitted on a training
    subsample after a Fisher-score prefilter (documented speed approximation)."""
    from scipy.optimize import minimize
    rng = np.random.default_rng(seed)
    mu, sd = X.mean(0), X.std(0) + 1e-12
    Z = (X - mu) / sd
    m1, m2 = Z[y == 1].mean(0), Z[y == 2].mean(0)
    v1, v2 = Z[y == 1].var(0), Z[y == 2].var(0)
    fisher = (m1 - m2) ** 2 / (v1 + v2 + 1e-12)
    cand = np.argsort(-fisher, kind="stable")[:min(prefilter, X.shape[1])]
    idx = rng.choice(X.shape[0], size=min(n_sub, X.shape[0]), replace=False)
    Zs, ys = Z[np.ix_(idx, cand)], y[idx]
    n, d = Zs.shape
    lam = 1.0 / n if lam is None else lam
    A = np.abs(Zs[:, None, :] - Zs[None, :, :]).astype(np.float32)  # n x n x d
    Y = (ys[:, None] == ys[None, :]).astype(np.float64)
    eye = np.eye(n, dtype=bool)

    def fg(w):
        w2 = w ** 2
        Dw = A @ w2.astype(np.float32)
        Dm = np.where(eye, np.inf, Dw)
        K = np.exp(-(Dm - Dm.min(1, keepdims=True)) / sigma)
        P = K / np.maximum(K.sum(1, keepdims=True), 1e-300)
        p = (P * Y).sum(1)
        f = -p.sum() / n + lam * w2.sum()
        S1 = np.einsum("ij,ijd->d", P * p[:, None], A)
```

```
        S2 = np.einsum("ij,ijd->d", P * Y, A)
        g = -(2 * w / sigma) * (S1 - S2) / n + 2 * lam * w
        return f, g
    res = minimize(fg, np.ones(d), jac=True, method="L-BFGS-B", options={"maxiter": 60})
    w = np.abs(res.x)
    score = np.full(X.shape[1], -1.0)
    score[cand] = w
    order = np.argsort(-score, kind="stable")
    return order, score[order]


RANKERS = {"mrmr": mrmr, "chi2": chi2_rank, "relieff": relieff, "nca": nca_rank}


def cwi_start(scores, mass=0.05):
    """First prefix (1-based count) whose cumulative shifted score mass reaches `mass`."""
    s = np.asarray(scores, float)
    s = s - s.min()
    tot = s.sum()
    if tot <= 0:
        return 1
    c = np.cumsum(s) / tot
    return int(np.searchsorted(c, mass) + 1)
```

4.11. Python/validation/rfp/tknn.py

tkNN configuration and iterative majority voting

```
"""tkNN (90 kNN configurations) and IMV ensembles with an incremental distance engine.

Candidate numbering follows the classifier-nested MATLAB run:
  candidates   1-90 : weight (squared-inverse, inverse, equal) x metric
                 (city-block, Euclidean, cosine) x k (1..10), k fastest;
                 e.g. 59 = inverse/cosine/k=9, 30 = squared-inverse/cosine/k=10.
  candidates 91-178 : IMV ensembles of the 3..90 highest-ranked configurations.
"""
import numpy as np
from scipy.spatial.distance import cdist

METRICS = ["cityblock", "euclidean", "cosine"]
WEIGHTS = ["squaredinverse", "inverse", "equal"]
KMAX = 10
CONFIGS = [(w, m, k) for w in WEIGHTS for m in METRICS for k in range(1, KMAX + 1)]
N_SINGLE = len(CONFIGS)           # 90
N_CAND = N_SINGLE + (N_SINGLE - 2)  # 178
```

```
def candidate_name(c):
    """1-based candidate index -> readable string."""
    if c <= N_SINGLE:
        w, m, k = CONFIGS[c - 1]
        return f"tkNN[{c}] {m}/{w}/k={k}"
    return f"IMV[{c}] top-{c - N_SINGLE + 2}"


# ------------------------------------------------------------- voting
def _weights(d, scheme):
    if scheme == "equal":
        return np.ones_like(d)
    if scheme == "inverse":
        return 1.0 / np.maximum(d, 1e-12)
    return 1.0 / np.maximum(d, 1e-12) ** 2


def vote_all(nn_lab, nn_dist):
    """nn_lab, nn_dist: (n, KMAX) sorted by distance, per metric.
    Returns preds (n, 30) for the (weight, k) grid of one metric and class-2 vote shares."""
    n = nn_lab.shape[0]
    preds = np.empty((n, len(WEIGHTS) * KMAX), np.int8)
    share = np.empty((n, len(WEIGHTS) * KMAX), np.float32)
    is2 = (nn_lab == 2)
    for wi, w in enumerate(WEIGHTS):
        W = _weights(nn_dist, w)
        c2 = np.cumsum(W * is2, 1)
        c1 = np.cumsum(W * ~is2, 1)
        sl = slice(wi * KMAX, (wi + 1) * KMAX)
        preds[:, sl] = np.where(c2 > c1, 2, 1)
        share[:, sl] = c2 / np.maximum(c1 + c2, 1e-300)
    return preds, share


def _reorder(per_metric):
    """Combine per-metric (n, 30) blocks [weight-major] into candidate order 1..90."""
    n = per_metric[0].shape[0]
    out = np.empty((n, N_SINGLE), per_metric[0].dtype)
    for mi in range(len(METRICS)):
        blk = per_metric[mi]
        for wi in range(len(WEIGHTS)):
            dst = wi * 30 + mi * 10
            out[:, dst:dst + KMAX] = blk[:, wi * KMAX:(wi + 1) * KMAX]
    return out
```

```
def knn_from_D(D, y_ref, kmax=KMAX):
    k = min(kmax, D.shape[1])
    nn = np.argpartition(D, k - 1, axis=1)[:, :k]
    dd = np.take_along_axis(D, nn, 1)
    o = np.argsort(dd, 1, kind="stable")
    nn = np.take_along_axis(nn, o, 1)
    dd = np.take_along_axis(dd, o, 1)
    lab = y_ref[nn]
    if k < kmax:  # pad (only for tiny training sets)
        lab = np.pad(lab, ((0, 0), (0, kmax - k)), mode="edge")
        dd = np.pad(dd, ((0, 0), (0, kmax - k)), mode="edge")
    return lab, dd


# ---------------------------------------------------------------- inner LOSO scan
def inner_scan(Z, y, g, prefixes):
    """Leave-one-participant-out scan over feature prefixes inside a training set.

    Z must already be standardised and column-ordered by the ranker. Distances are
    updated incrementally, so every prefix in `prefixes` is evaluated exactly.
    Returns dict with the best prefix (by best single-config pooled accuracy,
    ties -> smaller prefix), its 90 accuracies and predictions, and the full
    accuracy curve.
    """
    n = Z.shape[0]
    Z = Z.astype(np.float32)
    same = g[:, None] == g[None, :]
    Dc = np.zeros((n, n), np.float32)
    Dot = np.zeros((n, n), np.float32)
    nrm = np.zeros(n, np.float32)
    done = 0
    best = None
    curve = []
    for p in sorted(set(int(q) for q in prefixes)):
        blk = Z[:, done:p]
        for j in range(blk.shape[1]):
            c = blk[:, j]
            Dc += np.abs(c[:, None] - c[None, :])
        Dot += blk @ blk.T
        nrm += (blk ** 2).sum(1)
        done = p
        De = np.sqrt(np.maximum(nrm[:, None] + nrm[None, :] - 2 * Dot, 0))
        den = np.sqrt(np.maximum(nrm[:, None] * nrm[None, :], 1e-30))
```

```
        Dcos = 1.0 - Dot / den
        per = []
        for D in (Dc, De, Dcos):
            DD = np.where(same, np.inf, D)
            lab, dd = knn_from_D(DD, y)
            per.append(vote_all(lab, dd)[0])
        preds = _reorder(per)
        acc = (preds == y[:, None]).mean(0)
        curve.append((p, float(acc.max())))
        if best is None or acc.max() > best["acc"].max() + 1e-12:
            best = {"prefix": p, "acc": acc, "preds": preds}
    best["curve"] = np.array(curve)
    return best


def imv_select(acc, preds, y):
    """Rank the 90 configurations by inner accuracy and evaluate IMV ensembles.
    Returns (best candidate 1..178, candidate accuracies (178,), ranking)."""
    rank = np.lexsort((np.arange(N_SINGLE), -acc))   # stable: ties -> lower index
    cand_acc = np.empty(N_CAND)
    cand_acc[:N_SINGLE] = acc
    P = preds[:, rank]
    for m in range(3, N_SINGLE + 1):
        cand_acc[N_SINGLE + m - 3] = (imv_vote(P[:, :m]) == y).mean()
    best = int(np.argmax(cand_acc)) + 1
    return best, cand_acc, rank


def imv_vote(P):
    """Majority vote over columns of P (labels 1/2); ties -> first (best) column."""
    v2 = (P == 2).sum(1)
    v1 = P.shape[1] - v2
    out = np.where(v2 > v1, 2, np.where(v1 > v2, 1, P[:, 0]))
    return out.astype(np.int8)


# -------------------------------------------------------------- refit + predict
def predict(Ztr, ytr, Zte, candidate, rank):
    """Predict test rows with candidate (1-based) after refitting on all training rows."""
    if candidate <= N_SINGLE:
        cfgs = [candidate - 1]
    else:
        cfgs = list(rank[:candidate - N_SINGLE + 2])
    need = sorted({CONFIGS[c][1] for c in cfgs})
    nn = {}
```

```
    for m in need:
        D = cdist(Zte, Ztr, metric=m)
        nn[m] = knn_from_D(D, ytr)
    cols, shares = [], []
    for c in cfgs:
        w, m, k = CONFIGS[c]
        lab, dd = nn[m]
        lab, dd = lab[:, :k], dd[:, :k]
        W = _weights(dd, w)
        c2 = (W * (lab == 2)).sum(1)
        c1 = (W * (lab != 2)).sum(1)
        cols.append(np.where(c2 > c1, 2, 1))
        shares.append(c2 / np.maximum(c1 + c2, 1e-300))
    P = np.stack(cols, 1)
    if len(cfgs) == 1:
        return P[:, 0].astype(np.int8), shares[0]
    return imv_vote(P), (P == 2).mean(1)
```

4.12. Python/validation/rfp/stats.py

Classification metrics, confidence intervals, and statistical tests

```
"""Classification metrics, confidence intervals, and statistical tests."""
import numpy as np
from scipy import stats


def confusion(y, p):
    tn = int(((y == 1) & (p == 1)).sum()); fp = int(((y == 1) & (p == 2)).sum())
    fn = int(((y == 2) & (p == 1)).sum()); tp = int(((y == 2) & (p == 2)).sum())
    return tn, fp, fn, tp


def metrics(y, p):
    tn, fp, fn, tp = confusion(y, p)
    n = tn + fp + fn + tp
    sens = tp / max(tp + fn, 1); spec = tn / max(tn + fp, 1)
    ppv = tp / max(tp + fp, 1); npv = tn / max(tn + fn, 1)
    f1 = 2 * ppv * sens / max(ppv + sens, 1e-12)
    den = np.sqrt(float((tp + fp) * (tp + fn) * (tn + fp) * (tn + fn)))
    mcc = (tp * tn - fp * fn) / den if den > 0 else 0.0
    return dict(n=n, correct=tn + tp, acc=(tn + tp) / n, sens=sens, spec=spec,
                bacc=(sens + spec) / 2, ppv=ppv, npv=npv, f1=f1, mcc=mcc,
                tn=tn, fp=fp, fn=fn, tp=tp)
```

```
def wilson(k, n, z=1.959963984540054):
    if n == 0:
        return (np.nan, np.nan)
    ph = k / n
    den = 1 + z * z / n
    c = (ph + z * z / (2 * n)) / den
    h = z * np.sqrt(ph * (1 - ph) / n + z * z / (4 * n * n)) / den
    return (max(0.0, c - h), min(1.0, c + h))


def majority(y, p, g, score=None):
    """Participant majority vote. Ties -> mean class-2 score > 0.5 (else control)."""
    ids = np.unique(g)
    yt, pt, ties = [], [], 0
    for s in ids:
        m = g == s
        v2 = (p[m] == 2).sum(); v1 = m.sum() - v2
        if v2 > v1:
            q = 2
        elif v1 > v2:
            q = 1
        else:
            ties += 1
            q = 2 if (score is not None and score[m].mean() > 0.5) else 1
        yt.append(y[m][0]); pt.append(q)
    return np.array(yt), np.array(pt), ids, ties


def cluster_bootstrap_acc(y, p, g, B=2000, seed=2026):
    """Participant-resampled percentile CI for pooled epoch accuracy."""
    rng = np.random.default_rng(seed)
    ids = np.unique(g)
    corr = np.array([(y[g == s] == p[g == s]).sum() for s in ids], float)
    cnt = np.array([(g == s).sum() for s in ids], float)
    bs = np.empty(B)
    for b in range(B):
        i = rng.integers(0, ids.size, ids.size)
        bs[b] = corr[i].sum() / cnt[i].sum()
    return tuple(np.percentile(bs, [2.5, 97.5]))


def cluster_bootstrap_diff(y, pa, pb, g, B=2000, seed=2026):
    rng = np.random.default_rng(seed)
    ids = np.unique(g)
    ca = np.array([(y[g == s] == pa[g == s]).sum() for s in ids], float)
```

```
    cb = np.array([(y[g == s] == pb[g == s]).sum() for s in ids], float)
    cnt = np.array([(g == s).sum() for s in ids], float)
    obs = (ca.sum() - cb.sum()) / cnt.sum()
    bs = np.empty(B)
    for b in range(B):
        i = rng.integers(0, ids.size, ids.size)
        bs[b] = (ca[i].sum() - cb[i].sum()) / cnt[i].sum()
    return obs, tuple(np.percentile(bs, [2.5, 97.5]))


def mcnemar_exact(correct_a, correct_b):
    b = int((correct_a & ~correct_b).sum()); c = int((~correct_a & correct_b).sum())
    p = stats.binomtest(min(b, c), b + c, 0.5).pvalue if b + c > 0 else 1.0
    return b, c, float(p)


def perm_p(obs, null):
    null = np.asarray(null)
    return (1 + np.sum(null >= obs - 1e-12)) / (1 + null.size)


def welch_from_summary(m1, s1, n1, m2, s2, n2):
    se = np.sqrt(s1 ** 2 / n1 + s2 ** 2 / n2)
    t = (m2 - m1) / se
    df = se ** 4 / ((s1 ** 2 / n1) ** 2 / (n1 - 1) + (s2 ** 2 / n2) ** 2 / (n2 - 1))
    p = 2 * stats.t.sf(abs(t), df)
    q = stats.t.ppf(0.975, df)
    sp = np.sqrt(((n1 - 1) * s1 ** 2 + (n2 - 1) * s2 ** 2) / (n1 + n2 - 2))
    d = (m2 - m1) / sp
    J = 1 - 3 / (4 * (n1 + n2) - 9)
    return dict(diff=m2 - m1, se=se, t=t, df=df, p=p, ci=(m2 - m1 - q * se, m2 - m1 + q * se),
                d=d, g=d * J)


def hypergeom_summary(N, K, n, observed=None):
    rv = stats.hypergeom(N, K, n)
    out = dict(expected=n * K / N, lo=int(rv.ppf(0.025)), hi=int(rv.ppf(0.975)))
    if observed is not None:
        out["observed"] = int(observed)
        out["p_enrich"] = float(rv.sf(observed - 1))
        out["p_deplete"] = float(rv.cdf(observed))
    return out
```

4.13. Python/validation/rfp/features.py

```
import numpy as np
from scipy.signal import welch

FS = 256
CHANNELS = ["Cz", "Fz", "Fp1", "F7", "F3", "FC1", "C3", "FC5", "FT9", "T7", "CP5", "CP1",
        "P3", "P7", "PO9", "O1", "Pz", "Oz", "O2", "O10", "P8", "P4", "CP2", "CP6",
        "T8", "FT10", "FC6", "C4", "FC2", "F4", "F8", "Fp2"]
NCH = 32
PERIPHERAL = ["FT9", "FT10", "Fp1", "Fp2", "F7", "F8", "T7", "T8"]
LAYERS = ["A", "B", "G", "T", "D", "AT", "GA", "TB", "BG"]
LAYER_BANDS = {"A": ("alpha", "alpha"), "B": ("beta", "beta"), "G": ("gamma", "gamma"),
          "T": ("theta", "theta"), "D": ("delta", "delta"), "AT": ("alpha", "theta"),
          "GA": ("gamma", "alpha"), "TB": ("theta", "beta"), "BG": ("beta", "gamma")}
BANDS = {"delta": (0.5, 4.0), "theta": (4.0, 8.0), "alpha": (8.0, 13.0),
     "beta": (13.0, 30.0), "gamma": (30.0, 80.0)}
LAGS = np.array([1, 2, 4, 8])
LAG_W = np.array([0.50, 0.25, 0.15, 0.10])
ALPHA_WITHIN, ALPHA_CROSS = 0.12, 0.14


# ---------------------------------------------------------------------------- indices
def decode(idx0, nch=NCH):
  """0-based feature index -> (layer, source channel, target channel).
  Vectorisation: source fastest, then target, then layer (MATLAB M(:))."""
  idx0 = np.asarray(idx0)
  per = nch * nch
  layer = idx0 // per
  w = idx0 % per
  src = w % nch
  tgt = w // nch
  return layer, src, tgt


def self_loop_indices(nch=NCH, n_layers=9):
  lay, s, t = decode(np.arange(n_layers * nch * nch), nch)
  return np.where(s == t)[0]


def peripheral_indices(nch=NCH, n_layers=9, channels=CHANNELS,
peripheral=PERIPHERAL):
  pset = {channels.index(c) for c in peripheral}
  lay, s, t = decode(np.arange(n_layers * nch * nch), nch)
  return np.where(np.isin(s, list(pset)) | np.isin(t, list(pset)))[0]
```

```
def layer_indices(layer_name, nch=NCH):
    k = LAYERS.index(layer_name)
    return np.arange(k * nch * nch, (k + 1) * nch * nch)


def describe(idx0):
    lay, s, t = decode(idx0)
    return [f"{LAYERS[a]}:{CHANNELS[b]}->{CHANNELS[c]}" for a, b, c in zip(lay, s, t)]


# ------------------------------------------------------------------------ band power
def band_power(E, fs=FS):
    f, P = welch(E, fs=fs, nperseg=2 * fs, noverlap=fs, axis=1)   # (n, F, C)
    total_mask = (f >= 0.5) & (f <= 80)
    df = f[1] - f[0]
    tot = P[:, total_mask, :].sum(1) * df
    absp, relp = [], []
    for b, (lo, hi) in BANDS.items():
        m = (f >= lo) & (f < hi)
        bp = P[:, m, :].sum(1) * df
        absp.append(bp)
        relp.append(bp / np.maximum(tot, 1e-20))
    absp = np.log10(np.maximum(np.concatenate(absp, 1), 1e-20))
    relp = np.concatenate(relp, 1)
    return {"abs": absp, "rel": relp, "comb": np.concatenate([absp, relp], 1)}


def diagonal_mass(X, nch=NCH, n_layers=9):
    """Sum of the C diagonal entries per layer (features must be the raw unit-sum layers)."""
    per = nch * nch
    di = np.arange(nch) * nch + np.arange(nch)
    return np.stack([X[:, k * per + di].sum(1) for k in range(n_layers)], 1)
```

4.14. Python/validation/rfp/io.py

Data loading and preprocessing utilities

```
import os
import numpy as np


def _load_mat(path):
    try:
        from scipy.io import loadmat
        d = loadmat(path)
```

```
        return {k: v for k, v in d.items() if not k.startswith("__")}
    except NotImplementedError:  # v7.3 = HDF5
        import h5py  # pip install h5py
        out = {}
        with h5py.(path, "r") as f:
            for k in f.keys():
                # MATLAB stores column-major; h5py returns the transpose
                out[k] = np.array(f[k]).T
        return out


def load_any(path):
    ext = os.path.splitext(path)[1].lower()
    if ext == ".mat":
        return _load_mat(path)
    if ext == ".npz":
        return dict(np.load(path, allow_pickle=False))
    if ext == ".npy":
        return {"X": np.load(path)}
    raise ValueError(f"Unsupported  type: {path}")


def _pick(d, names):
    for n in names:
        if n in d:
            return d[n]
    raise KeyError(f"None of {names} found. Available: {list(d)}")


def load_features(path):
    d = load_any(path)
    X = np.asarray(_pick(d, ["X", "features", "feat", "F"]), dtype=np.float64)
    y = np.asarray(_pick(d, ["y", "labels", "label", "Y"])).ravel().astype(int)
    g = np.asarray(_pick(d, ["subj", "subject", "groups", "g", "pid", "participant"])).ravel()
    if X.shape[0] != y.size and X.shape[1] == y.size:
        X = X.T
    if set(np.unique(y)) == {0, 1}:
        y = y + 1  # code as 1 = control, 2 = recorded-offence
    assert set(np.unique(y)) <= {1, 2}, "labels must be 1 (control) / 2 (recorded-offence)"
    assert X.shape[0] == y.size == g.size
    X = np.nan_to_num(X, nan=0.0, posinf=0.0, neginf=0.0)
    g_raw = np.array([str(v).strip() for v in g])
    return X, y, _as_int_groups(g), g_raw
```

```
def _as_int_groups(g):
    _, inv = np.unique(g, return_inverse=True)
    return inv.astype(int) + 1


def load_epochs(path):
    """Return E as (n_epochs, n_samples, n_channels)."""
    d = load_any(path)
    E = np.asarray(_pick(d, ["E", "epochs", "eeg", "EEG", "data"]), dtype=np.float32)
    if E.ndim != 3:
        raise ValueError("epochs must be 3-D")
    # make samples the middle axis (expect 3840 samples, 32 channels)
    if E.shape[1] < E.shape[2]:
        E = np.transpose(E, (0, 2, 1))
    return E


def load_ages(path, g_raw=None):
    import csv
    ages = {}
    with open(path, newline="") as f:
        r = csv.DictReader(f)
        cols = {c.lower(): c for c in r.fieldnames}
        sc = cols.get("subj") or cols.get("subject") or cols.get("id")
        ac = cols.get("age")
        for row in r:
            ages[str(row[sc]).strip()] = float(row[ac])
    return ages


def load_panel(path):
    """Text  with 1-based RhythmFluxPat indices (one per line or comma separated)."""
    txt = open(path).read().replace(",", " ").split()
    idx = np.array([int(float(t)) for t in txt], dtype=int)
    return idx - 1  # 0-based
```

4.15. Python/validation/rfp/simdata.py

Simulated cohort for methodological evaluation (Appendix B)

```
"""Simulated cohort with the study's participant/epoch structure (for code tests
and the leakage demonstration). Contains no study data."""
import numpy as np

CTRL_EPOCHS = [28] * 50 + [30]                    # 51 participants, 1,430 epochs
OFF_EPOCHS = [28] * 38 + [7, 8, 10, 12, 16, 7]        # 44 participants, 1,124 epochs
```

```
def cohort_structure():
    y, g = [], []
    sid = 0
    for lab, counts in ((1, CTRL_EPOCHS), (2, OFF_EPOCHS)):
        for c in counts:
            sid += 1
            y += [lab] * c; g += [sid] * c
    return np.array(y), np.array(g)


def noise_features(d=9216, icc=0.5, effect=0.0, n_informative=0, seed=0):
    rng = np.random.default_rng(seed)
    y, g = cohort_structure()
    subs = np.unique(g)
    b = rng.normal(0, np.sqrt(icc), size=(subs.size, d))
    X = b[g - 1] + rng.normal(0, np.sqrt(1 - icc), size=(y.size, d))
    if n_informative:
        X[:, :n_informative] += effect * (y[:, None] == 2)
    return X.astype(np.float64), y, g
```

4.16. Python/validation/rfp/__init__.py

Python package initialization

4.17. Python/validation/requirements.txt

Python dependencies

```
numpy>=1.24
scipy>=1.10
scikit-learn>=1.3
joblib>=1.3
matplotlib>=3.7
h5py>=3.8
statsmodels>=0.14
```

4.18. Python/verification/analyze.py

```
#!/usr/bin/env python3
"""Recompute every reported quantity from file_sonuclari.mat.

No model is fitted here. All numbers are derived from the arrays saved by
MATLAB: actual_labels, tahmin_loso, mak_loso, idx, thm, acc_mv_loso, Cc_loso,
sonlar, selNCA, selCHI2, selMRMR, selRELIEFF, y and newIndices.
```

```
Usage: python analyze_file_mat.py file_sonuclari.mat out_dir
"""
import json
import math
import os
import sys
from collections import Counter

import numpy as np
import scipy.io as sio

CH = ['Cz', 'Fz', 'Fp1', 'F7', 'F3', 'FC1', 'C3', 'FC5', 'FT9', 'T7', 'CP5', 'CP1',
      'P3', 'P7', 'PO9', 'O1', 'Pz', 'Oz', 'O2', 'O10', 'P8', 'P4', 'CP2', 'CP6',
      'T8', 'FT10', 'FC6', 'C4', 'FC2', 'F4', 'F8', 'Fp2']
LAYERS = ['A', 'B', 'G', 'T', 'D', 'AT', 'GA', 'TB', 'BG']
PERIPHERAL = {'FT9', 'FT10', 'Fp1', 'Fp2', 'F7', 'F8', 'T7', 'T8'}
BRANCH_LABEL = {
    'son1_NCA': 'CWINCA', 'son2_Chi2': 'CWIChi2', 'son3_mRMR': 'CWImRMR',
    'son4_ReliefF': 'CWIReliefF', 'son5_NCA_Chi2_inter': 'NCA ∩ Chi2',
    'son6_NCA_mRMR_inter': 'NCA ∩ mRMR', 'son7_NCA_ReliefF_inter': 'NCA ∩ ReliefF',
    'son8_Chi2_mRMR_inter': 'Chi2 ∩ mRMR', 'son9_Chi2_ReliefF_inter': 'Chi2 ∩ ReliefF',
    'son10_mRMR_ReliefF_inter': 'mRMR ∩ ReliefF'}


def decode(index_1based):
    i = int(index_1based) - 1
    layer, within = divmod(i, 1024)
    target, source = divmod(within, 32)   # source varies fastest
    return LAYERS[layer], CH[source], CH[target]


def wilson(k, n, z=1.959963984540054):
    if n == 0:
        return (float('nan'), float('nan'))
    p = k / n
    den = 1 + z * z / n
    c = (p + z * z / (2 * n)) / den
    h = z * math.sqrt(p * (1 - p) / n + z * z / (4 * n * n)) / den
    return (max(0.0, c - h), min(1.0, c + h))


def cm2(actual, pred):
    cm = np.zeros((2, 2), int)
    for a, p in zip(actual, pred):
```

```
        cm[a - 1, p - 1] += 1
    return cm


def metrics(cm):
    tn, fp, fn, tp = int(cm[0, 0]), int(cm[0, 1]), int(cm[1, 0]), int(cm[1, 1])
    n = tn + fp + fn + tp
    sens = tp / (tp + fn)
    spec = tn / (tn + fp)
    ppv = tp / (tp + fp) if tp + fp else float('nan')
    npv = tn / (tn + fn) if tn + fn else float('nan')
    f1 = 2 * tp / (2 * tp + fp + fn)
    den = math.sqrt((tp + fp) * (tp + fn) * (tn + fp) * (tn + fn))
    mcc = (tp * tn - fp * fn) / den if den else float('nan')
    return dict(n=n, correct=tn + tp, acc=(tn + tp) / n, sens=sens, spec=spec,
                bacc=(sens + spec) / 2, ppv=ppv, npv=npv, f1=f1, mcc=mcc,
                cm=[[tn, fp], [fn, tp]])


def mode_smallest(cols):
    """MATLAB mode(): most frequent value; ties resolved to the smallest value."""
    out = np.empty(cols.shape[0], int)
    for r in range(cols.shape[0]):
        c = Counter(cols[r].tolist())
        best = max(c.values())
        out[r] = min(v for v, n in c.items() if n == best)
    return out


def runs(v):
    out, start = [], 0
    for i in range(1, len(v) + 1):
        if i == len(v) or v[i] != v[i - 1]:
            out.append((int(v[start]), i - start, start))
            start = i
    return out


def fold_order_ids(actual, ids_y, y):
    """Rebuild participant identifiers in the order of actual_labels.

    actual_labels is a concatenation of complete participant blocks. Within each
    class, participants are consumed in ascending identifier order. Every block
    must lie inside a single run of its own class; any violation raises.
    """
```

```
    uniq = np.unique(ids_y)
    length = {s: int((ids_y == s).sum()) for s in uniq}
    label = {s: int(y[ids_y == s][0]) for s in uniq}
    queue = {c: sorted(s for s in uniq if label[s] == c) for c in (1, 2)}
    out = np.zeros(len(actual), int)
    pos = 0
    while pos < len(actual):
        c = int(actual[pos])
        s = queue[c].pop(0)
        L = length[s]
        if not np.all(actual[pos:pos + L] == c):
            raise RuntimeError(f'participant {s} does not fit at row {pos}')
        out[pos:pos + L] = s
        pos += L
    assert not queue[1] and not queue[2]
    # every participant's block must end at a run boundary or be followed by
    # another block of the same class with a length that keeps the run intact
    return out


def participant_vote(actual, pred, ids):
    rows = []
    for s in sorted(np.unique(ids)):
        m = ids == s
        a = int(actual[m][0])
        p = pred[m]
        n1, n2 = int((p == 1).sum()), int((p == 2).sum())
        tie = n1 == n2
        vote = 1 if n1 >= n2 else 2          # MATLAB mode tie rule -> 1
        rows.append(dict(id=int(s), actual=a, n=int(m.sum()), n_pred1=n1, n_pred2=n2,
                         vote=vote, tie=tie, correct=vote == a))
    return rows


def participant_summary(rows):
    act = np.array([r['actual'] for r in rows])
    vote = np.array([r['vote'] for r in rows])
    cm = cm2(act, vote)
    met = metrics(cm)
    k, n = met['correct'], met['n']
    met['wilson_acc'] = wilson(k, n)
    met['wilson_sens'] = wilson(int(cm[1, 1]), int(cm[1].sum()))
    met['wilson_spec'] = wilson(int(cm[0, 0]), int(cm[0].sum()))
    met['ties'] = [r['id'] for r in rows if r['tie']]
    met['errors'] = [r['id'] for r in rows if not r['correct']]
```

```
    return met


def main(mat_path, out_dir):
    os.makedirs(out_dir, exist_ok=True)
    m = sio.loadmat(mat_path)
    y = m['y'].ravel().astype(int)
    ids_y = m['newIndices'].ravel().astype(int)
    actual = m['actual_labels'].ravel().astype(int)
    names = [str(x[0]) for x in m['son_isimleri'][0]]
    preds = [p.ravel().astype(int) for p in m['tahmin_loso'][0]]
    sonlar = [s for s in m['sonlar'][0]]
    mak = m['mak_loso'].ravel()
    idx = m['idx'].ravel().astype(int)
    thm = [t.ravel().astype(int) for t in m['thm'][0]]
    acc_mv = m['acc_mv_loso'].ravel()
    Cc = [c.astype(int) for c in m['Cc_loso'][0]]
    sel = {'NCA': m['selNCA'].ravel().astype(int), 'Chi2': m['selCHI2'].ravel().astype(int),
           'mRMR': m['selMRMR'].ravel().astype(int), 'ReliefF':
m['selRELIEFF'].ravel().astype(int)}

    R = {'': os.path.basename(mat_path), 'header': m['__header__'].decode(errors='ignore')}
    checks = []

    # ---- structural checks ------------------------------------------------
    R['n_epochs'] = int(len(y))
    R['n_participants'] = int(len(np.unique(ids_y)))
    R['class_counts_epochs'] = {int(k): int(v) for k, v in Counter(y.tolist()).items()}
    R['participants_per_class'] = {c: int(len({s for s in np.unique(ids_y) if y[ids_y == s][0] ==
c}))
                                   for c in (1, 2)}
    checks.append(('each participant has one label',
                   all(len(np.unique(y[ids_y == s])) == 1 for s in np.unique(ids_y))))
    checks.append(('actual_labels is a permutation of y', sorted(actual.tolist()) ==
sorted(y.tolist())))
    for i, S in enumerate(sonlar):
        checks.append((f'sonlar{{{i+1}}} last column equals y', bool(np.array_equal(S[:, -
1].astype(int), y))))
    ids_fold = fold_order_ids(actual, ids_y, y)
    checks.append(('fold-order participant reconstruction consistent', True))
    R['fold_order_participants'] = [r[0] for r in runs(ids_fold)]

    # ---- feature sets -------------------------------------------------------
    fs = {}
    for k, v in sel.items():
```

```
        fs[k] = dict(n=int(len(v)), unique=int(len(np.unique(v))), min=int(v.min()),
max=int(v.max()),
                  n_matrix_cols=None)
    for k, i in zip(['NCA', 'Chi2', 'mRMR', 'ReliefF'], range(4)):
        fs[k]['n_matrix_cols'] = int(sonlar[i].shape[1] - 1)
        checks.append((f'{k}: selected count equals sonlar columns', fs[k]['n'] ==
fs[k]['n_matrix_cols']))
    R['feature_sets'] = fs
    pairs = [('NCA', 'Chi2', 4), ('NCA', 'mRMR', 5), ('NCA', 'ReliefF', 6),
             ('Chi2', 'mRMR', 7), ('Chi2', 'ReliefF', 8), ('mRMR', 'ReliefF', 9)]
    base_pos = {'NCA': 0, 'Chi2': 1, 'mRMR': 2, 'ReliefF': 3}
    ov = []
    for a, b, si in pairs:
        A, B = set(sel[a].tolist()), set(sel[b].tolist())
        # MATLAB intersect(...,'stable'): order follows the rank order of the first selector
        inter = [int(f) for f in sel[a] if int(f) in B]
        jac = len(inter) / len(A | B)
        S = sonlar[si][:, :-1]
        same_cols = S.shape[1] == len(inter)
        # verify that the intersection matrix columns equal the base columns
        colmatch_a = colmatch_b = False
        if same_cols:
            pa = [int(np.where(sel[a] == f)[0][0]) for f in inter]
            pb = [int(np.where(sel[b] == f)[0][0]) for f in inter]
            Xa = sonlar[base_pos[a]][:, pa]
            Xb = sonlar[base_pos[b]][:, pb]
            colmatch_a = bool(np.allclose(S, Xa))
            colmatch_b = bool(np.allclose(S, Xb))
        ov.append(dict(a=a, b=b, n_inter=len(inter), n_union=len(A | B), jaccard=jac,
                       matrix_cols=int(S.shape[1]), cols_match_a=colmatch_a,
cols_match_b=colmatch_b,
                       features=[dict(index=f, decoded='%s %s->%s' % decode(f)) for f in inter]))
        checks.append((f'{a}∩{b}: size equals sonlar{{{si+1}}} columns', same_cols))
        checks.append((f'{a}∩{b}: columns identical to {a} and {b} source columns', colmatch_a
and colmatch_b))
    R['overlaps'] = ov

    # layer and peripheral composition per selector
    comp = {}
    for k, v in sel.items():
        dec = [decode(f) for f in v]
        lay = Counter(d[0] for d in dec)
        comp[k] = dict(layers={L: int(lay.get(L, 0)) for L in LAYERS},
                       self_transitions=int(sum(d[1] == d[2] for d in dec)),
```

```
                peripheral=int(sum((d[1] in PERIPHERAL) or (d[2] in PERIPHERAL) for d in
dec)))
    R['composition'] = comp

    # ---- branch performance ---------------------------------------------
    br = []
    for i, (nm, p) in enumerate(zip(names, preds)):
        cm = cm2(actual, p)
        e = metrics(cm)
        checks.append((f'{nm}: recomputed accuracy equals mak_loso', abs(e['acc'] - mak[i]) <
1e-12))
        prow = participant_vote(actual, p, ids_fold)
        ps = participant_summary(prow)
        br.append(dict(rank=int(np.where(idx == i + 1)[0][0]) + 1, name=nm,
label=BRANCH_LABEL[nm],
                n_features=int(sonlar[i].shape[1] - 1), epoch=e, participant=ps,
                mak_loso=float(mak[i])))
    R['branches'] = br
    order = np.argsort(-mak, kind='stable') + 1
    checks.append(('idx equals branches sorted by descending accuracy',
bool(np.array_equal(order, idx))))

    # ---- IMV over ranked branches ---------------------------------------
    imv = []
    P = np.column_stack(preds)
    for j in range(len(thm)):
        k = j + 3
        mine = mode_smallest(P[:, idx[:k] - 1])
        cm = cm2(actual, thm[j])
        e = metrics(cm)
        ties = int(sum(1 for r in P[:, idx[:k] - 1] if (r == 1).sum() == (r == 2).sum()))
        checks.append((f'IMV top-{k}: thm equals mode of ranked predictions',
bool(np.array_equal(mine, thm[j]))))
        checks.append((f'IMV top-{k}: accuracy equals acc_mv_loso', abs(e['acc'] - acc_mv[j]) <
1e-12))
        checks.append((f'IMV top-{k}: Cc_loso equals confusion matrix',
bool(np.array_equal(Cc[j], cm))))
        prow = participant_vote(actual, thm[j], ids_fold)
        ps = participant_summary(prow)
        imv.append(dict(k=k, members=[BRANCH_LABEL[names[t - 1]] for t in idx[:k]],
epoch=e,
                 participant=ps, epoch_vote_ties=ties, acc_mv_loso=float(acc_mv[j])))
    R['imv'] = imv

    # ---- per-participant tables for primary branches ----------------------
```

```
    best_branch = int(idx[0]) - 1
    best_imv = int(np.argmax(acc_mv))
    R['participant_table_best_branch'] = participant_vote(actual, preds[best_branch],
ids_fold)
    R['participant_table_best_imv'] = participant_vote(actual, thm[best_imv], ids_fold)

    # agreement between best single branch and best IMV
    a, b = preds[best_branch], thm[best_imv]
    R['best_branch_vs_best_imv'] = dict(
        changed=int((a != b).sum()),
        improved=int(((a != actual) & (b == actual)).sum()),
        worsened=int(((a == actual) & (b != actual)).sum()))

    # ---- DRLobish consistency on the mRMR panel --------------------------
    X = sonlar[2][:, :-1]
    subs = np.unique(ids_y)
    lab = np.array([y[ids_y == s][0] for s in subs])
    M = np.vstack([X[ids_y == s].mean(axis=0) for s in subs])
    m1, m2 = M[lab == 1].mean(axis=0), M[lab == 2].mean(axis=0)
    assign = np.where(m2 > m1, 2, 1)

    def auc_g(col):
        x1, x2 = col[lab == 1], col[lab == 2]
        gt = (x2[:, None] > x1[None, :]).sum() + 0.5 * (x2[:, None] == x1[None, :]).sum()
        auc = gt / (len(x1) * len(x2))
        n1, n2 = len(x1), len(x2)
        sp = math.sqrt(((n1 - 1) * x1.var(ddof=1) + (n2 - 1) * x2.var(ddof=1)) / (n1 + n2 - 2))
        d = (x2.mean() - x1.mean()) / sp
        J = 1 - 3 / (4 * (n1 + n2) - 9)
        return max(auc, 1 - auc), abs(d * J)

    route_rows = []
    for j, f in enumerate(sel['mRMR']):
        au, g = auc_g(M[:, j])
        L, s, t = decode(f)
        route_rows.append(dict(rank=j + 1, index=int(f), layer=L, source=s, target=t,
                               assigned='Control' if assign[j] == 1 else 'Recorded-offence',
                               auc=au, abs_g=g))
    R['mrmr_assignment'] = {'control': int((assign == 1).sum()), 'offence': int((assign ==
2).sum())}
    R['mrmr_layers_by_class'] = {c: dict(Counter(r['layer'] for r in route_rows if r['assigned']
== c))
                                 for c in ('Control', 'Recorded-offence')}
    probes = [('AT', 'T8', 'T8'), ('GA', 'C4', 'CP6'), ('D', 'FC2', 'F8'), ('GA', 'FT9', 'CP6'),
              ('D', 'FT10', 'FT10')]
```

```
    R['mrmr_probe_routes'] = [r for r in route_rows if (r['layer'], r['source'], r['target']) in
probes]
    R['mrmr_top_auc'] = sorted(route_rows, key=lambda r: -r['auc'])[:10]

    R['checks'] = [dict(check=c, passed=bool(p)) for c, p in checks]
    R['all_checks_passed'] = all(p for _, p in checks)

    with open(os.path.join(out_dir, 'file_results.json'), 'w') as fh:
        json.dump(R, fh, indent=1, default=float)
    with open(os.path.join(out_dir, 'mrmr_routes.csv'), 'w') as fh:
        fh.write('rank,index,layer,source,target,assigned,auc,abs_g\n')
        for r in route_rows:
            fh.write('%d,%d,%s,%s,%s,%s,%.4f,%.4f\n' % (r['rank'], r['index'], r['layer'],
r['source'],
                                   r['target'], r['assigned'], r['auc'], r['abs_g']))
    with open(os.path.join(out_dir, 'participant_votes_best_branch.csv'), 'w') as fh:
        fh.write('id,actual,n_epochs,pred_control,pred_offence,vote,tie,correct\n')
        for r in R['participant_table_best_branch']:
            fh.write('%d,%d,%d,%d,%d,%d,%d,%d\n' % (r['id'], r['actual'], r['n'], r['n_pred1'],
r['n_pred2'],
                                   r['vote'], r['tie'], r['correct']))

    # ---- console report ---------------------------------------------------
    print('checks passed:', R['all_checks_passed'])
    for c in R['checks']:
        if not c['passed']:
            print('  FAILED:', c['check'])
    print('\nBranch performance (epoch | participant)')
    for b in sorted(br, key=lambda b: b['rank']):
        e, p = b['epoch'], b['participant']
        print('%2d %-16s nF=%3d  epoch %4d/%d=%.2f%% sens %.2f spec %.2f bacc %.2f mcc
%.3f | part %d/95=%.2f%% '
              'sens %.2f spec %.2f ties %s err %s' % (
                  b['rank'], b['label'], b['n_features'], e['correct'], e['n'], 100 * e['acc'], 100 * e['sens'],
                  100 * e['spec'], 100 * e['bacc'], e['mcc'], p['correct'], 100 * p['acc'], 100 * p['sens'],
                  100 * p['spec'], p['ties'], p['errors']))
    print('\nIMV over ranked branches')
    for v in imv:
        e, p = v['epoch'], v['participant']
        print('top-%2d epoch %4d=%.2f%% cm %s sens %.2f spec %.2f | part %d=%.2f%% ties
%s err %s | epoch ties %d' % (
            v['k'], e['correct'], 100 * e['acc'], e['cm'], 100 * e['sens'], 100 * e['spec'], p['correct'],
            100 * p['acc'], p['ties'], p['errors'], v['epoch_vote_ties']))
    print('\nOverlaps')
    for o in ov:
```

```
        print('%-8s %-8s inter %3d union %3d jaccard %.2f%%' % (o['a'], o['b'], o['n_inter'],
o['n_union'],
                                                    100 * o['jaccard']))
    print('\nComposition', json.dumps(comp))
    print('\nmRMR class assignment', R['mrmr_assignment'])
    print('mRMR layers by class', R['mrmr_layers_by_class'])
    print('probe routes:')
    for r in R['mrmr_probe_routes']:
        print('  ', r)
    print('best branch vs best IMV', R['best_branch_vs_best_imv'])


if __name__ == '__main__':
    main(sys.argv[1], sys.argv[2])
```

4.19. Python/verification/tknn_verification.py

Independent verification of tkNN and IMV on saved feature panels (Appendix D)

```
#!/usr/bin/env python3
import json
import os
import sys
from collections import Counter

import numpy as np
import scipy.io as sio
from scipy.spatial.distance import cdist

sys.path.insert(0, os.path.dirname(os.path.abspath(__file__)))
from analyze_file_mat import fold_order_ids  # noqa: E402

WEIGHTS = ['squaredinverse', 'inverse', 'equal']
METRICS = ['cityblock', 'euclidean', 'cosine']


def config_list():
    out = []
    for w in WEIGHTS:
        for d in METRICS:
            for k in range(1, 11):
                out.append((w, d, k))
    return out


def loso_all_configs(X, y, ids):
```

```
    cfgs = config_list()
    P = np.zeros((len(y), len(cfgs)), int)
    for s in np.unique(ids):
        te = ids == s
        tr = ~te
        mu = X[tr].mean(axis=0)
        sd = X[tr].std(axis=0, ddof=1)
        sd[sd == 0] = 1.0
        Xtr = (X[tr] - mu) / sd
        Xte = (X[te] - mu) / sd
        ytr = y[tr]
        for d in METRICS:
            D = cdist(Xte, Xtr, metric=d)
            nn = np.argsort(D, axis=1, kind='stable')[:, :10]
            Dn = np.take_along_axis(D, nn, axis=1)
            Yn = ytr[nn]
            for w in WEIGHTS:
                if w == 'equal':
                    W = np.ones_like(Dn)
                elif w == 'inverse':
                    W = 1.0 / np.maximum(Dn, 1e-300)
                else:
                    W = 1.0 / np.maximum(Dn, 1e-300) ** 2
                for k in range(1, 11):
                    s1 = (W[:, :k] * (Yn[:, :k] == 1)).sum(axis=1)
                    s2 = (W[:, :k] * (Yn[:, :k] == 2)).sum(axis=1)
                    pred = np.where(s2 > s1, 2, 1)
                    P[te, cfgs.index((w, d, k))] = pred
    return P


def imv(P, y):
    acc = (P == y[:, None]).mean(axis=0)
    order = np.argsort(-acc, kind='stable')
    ens = []
    for n in range(3, P.shape[1] + 1):
        sub = P[:, order[:n]]
        c1 = (sub == 1).sum(axis=1)
        c2 = (sub == 2).sum(axis=1)
        ens.append(np.where(c2 > c1, 2, 1))
    return acc, order, np.column_stack(ens)


def main(mat_path, out_dir):
    m = sio.loadmat(mat_path)
```

```
    y = m['y'].ravel().astype(int)
    ids = m['newIndices'].ravel().astype(int)
    actual = m['actual_labels'].ravel().astype(int)
    names = [str(x[0]) for x in m['son_isimleri'][0]]
    saved = [p.ravel().astype(int) for p in m['tahmin_loso'][0]]
    mak = m['mak_loso'].ravel()

    # map y-order rows to fold-order rows (participant blocks keep epoch order)
    ids_fold = fold_order_ids(actual, ids, y)
    perm = np.concatenate([np.where(ids == s)[0] for s in [ids_fold[i] for i in
                           np.r_[0, np.where(np.diff(ids_fold) != 0)[0] + 1]]])
    assert np.array_equal(y[perm], actual), 'row mapping does not reproduce actual_labels'

    rows = []
    for i, S in enumerate(m['sonlar'][0]):
        X = S[:, :-1].astype(float)
        P = loso_all_configs(X, y, ids)
        acc, order, E = imv(P, y)
        allP = np.column_stack([P, E])
        allacc = (allP == y[:, None]).mean(axis=0)
        best = int(np.argmax(allacc))
        Pf = allP[perm]                              # fold order
        agree = (Pf == saved[i][:, None]).mean(axis=0)
        closest = int(np.argmax(agree))
        rows.append(dict(branch=names[i], saved_acc=float(mak[i]),
                  verification_best_single=float(acc.max()),
                  verification_best_single_cfg=list(config_list()[int(np.argmax(acc))]),
                  verification_best_178=float(allacc[best]),
                  verification_best_178_is_imv=best >= 90,
                  closest_candidate=closest, closest_agreement=float(agree[closest]),
                  closest_candidate_acc=float(allacc[closest]),
                  n_rows_differ_from_saved=int((Pf[:, closest] != saved[i]).sum())))
        r = rows[-1]
        print('%-26s saved %.4f | verification best single %.4f %s | best of 178 %.4f (IMV=%s) | closest cand %d agree %.4f (%d rows differ)' % (
            r['branch'], r['saved_acc'], r['verification_best_single'], r['verification_best_single_cfg'], r['verification_best_178'],
            r['verification_best_178_is_imv'], r['closest_candidate'], r['closest_agreement'], r['n_rows_differ_from_saved']),
            flush=True)
    with open(os.path.join(out_dir, 'tknn_verification.json'), 'w') as fh:
        json.dump(rows, fh, indent=1)


if __name__ == '__main__':
```

```
    main(sys.argv[1], sys.argv[2])
```

4.20. Python/verification/rhythmfluxpat.py

Python implementation of RhythmFluxPat.m

```
#!/usr/bin/env python3
"""Line-by-line Python port of the author MATLAB function RhythmFluxPat.m
(Supplementary Code S1, Section 4.1). Output: 1 x 9,216 feature vector in the
MATLAB column-major order idx = src + (tgt-1)*C + (layer-1)*C*C.
"""
import numpy as np
from scipy.signal import butter, filtfilt, hilbert

EPSI = 1e-10


def robust_global_z(X):
    c = np.median(X)
    mad = np.median(np.abs(X - c))
    Z = (X - c) / (1.4826 * mad + EPSI)
    Z[~np.isfinite(Z)] = 0
    return Z


def bpf(X, fs, lo, hi):
    nyq = fs / 2
    lo_n = max(lo / nyq, 1e-4)
    hi_n = min(hi / nyq, 0.999)
    if hi_n <= lo_n:
        return np.zeros_like(X)
    b, a = butter(4, [lo_n, hi_n], btype='bandpass')
    nfilt = max(len(a), len(b))
    Y = filtfilt(b, a, X, axis=0, padtype='odd', padlen=3 * (nfilt - 1))   # MATLAB filtfilt padding
    Y[~np.isfinite(Y)] = 0
    return Y


def zmap(M):
    Z = (M - M.mean()) / (M.std(ddof=1) + EPSI)
    Z[~np.isfinite(Z)] = 0
    return Z


def row_robust_z(M):
    med = np.median(M, axis=1, keepdims=True)
```

```
    C = M - med
    sc = 1.4826 * np.median(np.abs(C), axis=1, keepdims=True) + EPSI
    Z = C / sc
    Z[~np.isfinite(Z)] = 0
    return Z


def directed_wave_map(se, te, sp, tp):
    te_lead = np.vstack([te[1:], te[-1:]])
    tp_lead = np.vstack([tp[1:], tp[-1:]])
    amp = np.sqrt(np.abs(se * te_lead) + EPSI)
    ph = np.cos(sp - tp_lead)
    direction = np.tanh(row_robust_z(te_lead) - row_robust_z(se))
    C = amp * ph * (1 + 0.25 * direction)
    C = zmap(C)
    C[~np.isfinite(C)] = 0
    return C


def rank_order(M):
    nch = M.shape[1]
    key = 1e-12 * np.arange(1, nch + 1)
    # MATLAB sort(...,'descend') is stable: equal values keep ascending column order
    return np.argsort(-(M + key), axis=1, kind='stable')        # 0-based channel indices


def temporal_transition(order, Z, nch, lag):
    rc, rf = order[:-lag], order[lag:]
    mc, mf = Z[:-lag], Z[lag:]
    rows = np.arange(rc.shape[0])[:, None]
    sv = mc[rows, rc]
    tv = mf[rows, rf]
    w = 1 + 0.25 * np.tanh(0.5 * (sv + tv))
    w[~np.isfinite(w)] = 1
    w[w < 0] = 0
    M = np.zeros((nch, nch))
    np.add.at(M, (rc.ravel(), rf.ravel()), w.ravel())            # M[source, target]
    return M


def transition_contrast(M, alpha):
    s = M.sum()
    if s <= 0:
        return M
    P = M / (s + EPSI)
```

```
    E = P.sum(axis=1, keepdims=True) @ P.sum(axis=0, keepdims=True)
    E = E / (E.sum() + EPSI)
    Rm = P - alpha * E
    Rm[Rm < 0] = 0
    return (1 - alpha) * P + alpha * Rm


def make_table(mapx, nch, lags, lagw, alpha, return_raw=False):
    mapx = mapx.copy()
    mapx[~np.isfinite(mapx)] = 0
    Z = row_robust_z(mapx)
    order = rank_order(Z)
    M = np.zeros((nch, nch))
    raw = []
    for lag, w in zip(lags, lagw):
        T = temporal_transition(order, Z, nch, lag)
        raw.append(T)
        M += w * T
    M = transition_contrast(M, alpha)
    s = M.sum()
    if s > 0:
        M = M / s
    if return_raw:
        return M, order, raw, Z
    return M


def rhythmfluxpat(sig):
    X = np.asarray(sig, dtype=float)
    if X.shape[0] < X.shape[1]:
        X = X.T
    X[~np.isfinite(X)] = 0
    n, nch = X.shape
    fs = n / 15
    X = robust_global_z(X)
    ghi = min(80, 0.45 * fs)
    bands = {'D': (0.5, 4), 'T': (4, 8), 'A': (8, 13), 'B': (13, 30), 'G': (30, ghi)}
    H = {k: hilbert(bpf(X, fs, lo, hi), axis=0) for k, (lo, hi) in bands.items()}
    ENV = {k: np.abs(v) for k, v in H.items()}
    PHI = {k: np.angle(v) for k, v in H.items()}
    lags, lagw = [1, 2, 4, 8], [0.50, 0.25, 0.15, 0.10]
    mats = [make_table(ENV[k], nch, lags, lagw, 0.12) for k in ('A', 'B', 'G', 'T', 'D')]
    for s, t in (('A', 'T'), ('G', 'A'), ('T', 'B'), ('B', 'G')):
        C = directed_wave_map(ENV[s], ENV[t], PHI[s], PHI[t])
        mats.append(make_table(C, nch, lags, lagw, 0.14))
```

```
    out = []
    for M in mats:
        M[~np.isfinite(M)] = 0
        s = M.sum()
        if s > 0:
            M = M / s
        out.append(M.ravel(order='F'))                          # MATLAB mats(:)
    v = np.concatenate(out)
    v[~np.isfinite(v)] = 0
    return v, fs, ENV, mats
```

4.21. Python/verification/trace_raw_epochs.py

Raw EEG epoch feature extraction and consistency checks (Appendix C)

```
#!/usr/bin/env python3
"""Re-extract raw EEG epochs with RhythmFluxPat and trace them to the archive.

1. Runs the Python port of RhythmFluxPat.m on each raw epoch (blok_sinyal, 3,840 x 32).
2. Repeats the Appendix C checks on real EEG: rank invariance to row-wise robust
   standardisation (all nine maps) and doubly stochastic unweighted counts (all lags).
3. Locates the two epochs in the saved min-max-scaled panels. For any two rows,
   X[r2,j] - X[r1,j] = (f2_j - f1_j) / range_j with range_j > 0, so the sign of the
   between-epoch difference must match for every selected coordinate.

Usage: python trace_raw_epochs.py file_sonuclari.mat epoch_class1.mat epoch_class2.mat
out_dir
"""
import json
import os
import sys

import numpy as np
import scipy.io as sio
from scipy.signal import hilbert

sys.path.insert(0, os.path.dirname(os.path.abspath(____)))
from rhythmfluxpat import (bpf, directed_wave_map, rank_order, rhythmfluxpat,  # noqa:
E402
                           robust_global_z, row_robust_z)


def structure_checks(sig):
    X = robust_global_z(sig.astype(float))
    fs = X.shape[0] / 15
    bands = {'D': (0.5, 4), 'T': (4, 8), 'A': (8, 13), 'B': (13, 30), 'G': (30, min(80, 0.45 * fs))}
```

```
    H = {k: hilbert(bpf(X, fs, *v), axis=0) for k, v in bands.items()}
    E = {k: np.abs(v) for k, v in H.items()}
    P = {k: np.angle(v) for k, v in H.items()}
    maps = {k: E[k] for k in ('A', 'B', 'G', 'T', 'D')}
    for s, t in (('A', 'T'), ('G', 'A'), ('T', 'B'), ('B', 'G')):
        maps[s + t] = directed_wave_map(E[s], E[t], P[s], P[t])
    rank_same, ds = True, True
    for M in maps.values():
        o_z, o_raw = rank_order(row_robust_z(M)), rank_order(M)
        rank_same &= bool(np.array_equal(o_z, o_raw))
        for lag in (1, 2, 4, 8):
            C = np.zeros((32, 32))
            np.add.at(C, (o_z[:-lag].ravel(), o_z[lag:].ravel()), 1)
            ds &= bool(np.all(C.sum(0) == X.shape[0] - lag) and np.all(C.sum(1) == X.shape[0] -
lag))
    return dict(fs=fs, rank_identical_all_9_maps=rank_same,
doubly_stochastic_all_maps_lags=ds)


def main(archive, e1, e2, out_dir):
    os.makedirs(out_dir, exist_ok=True)
    res = {}
    F = []
    for name, f in (('class1', e1), ('class2', e2)):
        sig = sio.loadmat(f)['blok_sinyal']
        v, fs, _, mats = rhythmfluxpat(sig)
        F.append(v)
        res[name] = dict(=os.path.basename(f), shape=list(sig.shape), duration_s=sig.shape[0] /
fs,
                         n_features=int(v.size), all_finite=bool(np.isfinite(v).all()),
                         layer_sums=[float(m.sum()) for m in mats], **structure_checks(sig))
    F = np.vstack(F)

    m = sio.loadmat(archive)
    y = m['y'].ravel().astype(int)
    ids = m['newIndices'].ravel().astype(int)
    sel = [m['selNCA'].ravel(), m['selCHI2'].ravel(), m['selMRMR'].ravel(),
m['selRELIEFF'].ravel()]
    S = m['sonlar'][0]
    cols, feat = [], []
    for k in range(4):
        for j, f in enumerate(sel[k]):
            if int(f) not in feat:
                feat.append(int(f))
                cols.append(S[k][:, j])
```

```
    X = np.column_stack(cols)
    feat = np.array(feat)
    df = F[1, feat - 1] - F[0, feat - 1]
    sd = np.sign(df)
    r2s = np.where(y == 2)[0]
    best = []
    for r1 in np.where(y == 1)[0]:
        a = (np.sign(X[r2s] - X[r1]) == sd).mean(axis=1)
        k = int(np.argmax(a))
        best.append((float(a[k]), int(r1), int(r2s[k])))
    best.sort(reverse=True)

    def pos(r):
        rows = np.where(ids == ids[r])[0]
        return [int(ids[r]), int(np.where(rows == r)[0][0]) + 1, int(len(rows))]
    res['link'] = dict(n_union=int(len(feat)), agreement=best[0][0], second_best=best[1][0],
                       row1=pos(best[0][1]), row2=pos(best[0][2]),
                       note='row = [participant identifier, position within block, block length]')
    json.dump(res, open(os.path.join(out_dir, 'raw_epoch_trace.json'), 'w'), indent=1)
    print(json.dumps(res, indent=1))


if __name__ == '__main__':
    main(*sys.argv[1:5])
```

4.22. Python/verification/nested_svm.py

Participant-level SVM evaluation using the 352-feature panel (Section 2.2.7)

```
#!/usr/bin/env python3
"""Classifier-nested SVM on the saved 352-coordinate CWImRMR panel.

Outer loop: 95 participant-held-out folds. Inside each outer training set:
z-scoring fitted on the training participants; kernel and hyperparameters chosen
by participant-grouped five-fold CV (pooled inner epoch accuracy):
  linear: C in {0.001, 0.01, 0.1, 1}
  RBF:    C in {0.1, 1, 10, 100}, gamma in {0.25, 1, 4}/p
The selected model is refitted on all training participants and applied once to
the held-out participant. The panel itself was selected with all participants,
so feature selection is NOT nested here.

Usage: python nested_svm.py file_sonuclari.mat out_dir [n_jobs]
"""
import json
import os
import sys
```

```
import numpy as np
import scipy.io as sio
from joblib import Parallel, delayed
from sklearn.model_selection import GroupKFold
from sklearn.svm import SVC

SETTINGS = [('linear', C, None) for C in (0.001, 0.01, 0.1, 1)] + \
        [('rbf', C, g) for C in (0.1, 1, 10, 100) for g in (0.25, 1, 4)]


def kernel(A, B, kind, g, p):
    if kind == 'linear':
        return A @ B.T
    d = (A * A).sum(1)[:, None] + (B * B).sum(1)[None, :] - 2 * A @ B.T
    return np.exp(-(g / p) * np.maximum(d, 0))


def outer_fold(X, y, ids, s):
    tr, te = ids != s, ids == s
    mu, sd = X[tr].mean(0), X[tr].std(0, ddof=0)
    sd[sd == 0] = 1
    Z, Zt = (X[tr] - mu) / sd, (X[te] - mu) / sd
    ytr, gtr = y[tr], ids[tr]
    p = X.shape[1]
    G_lin = Z @ Z.T
    sq = np.diag(G_lin)
    D = np.maximum(sq[:, None] + sq[None, :] - 2 * G_lin, 0)
    folds = list(GroupKFold(n_splits=5).split(Z, ytr, gtr))
    acc = []
    for kind, C, g in SETTINGS:
        K = G_lin if kind == 'linear' else np.exp(-(g / p) * D)
        correct = 0
        for a, b in folds:
            m = SVC(kernel='precomputed', C=C).fit(K[np.ix_(a, a)], ytr[a])
            correct += int((m.predict(K[np.ix_(b, a)]) == ytr[b]).sum())
        acc.append(correct / len(ytr))
    best = int(np.argmax(acc))
    kind, C, g = SETTINGS[best]
    K = G_lin if kind == 'linear' else np.exp(-(g / p) * D)
    m = SVC(kernel='precomputed', C=C).fit(K, ytr)
    pred = m.predict(kernel(Zt, Z, kind, g, p))
    return int(s), np.where(te)[0].tolist(), pred.tolist(), best, float(acc[best])
```

```
def main(mat, out_dir, n_jobs=2):
    m = sio.loadmat(mat)
    X = m['sonlar'][0][2][:, :-1].astype(float)
    y = m['y'].ravel().astype(int)
    ids = m['newIndices'].ravel().astype(int)
    res = Parallel(n_jobs=n_jobs, verbose=5)(delayed(outer_fold)(X, y, ids, s) for s in
np.unique(ids))
    pred = np.zeros(len(y), int)
    folds = []
    for s, rows, p, best, ia in res:
        pred[rows] = p
        folds.append(dict(participant=s, setting=list(map(str, SETTINGS[best])), inner_acc=ia))
    np.save(os.path.join(out_dir, 'nested_svm_pred.npy'), pred)
    json.dump(folds, open(os.path.join(out_dir, 'nested_svm_folds.json'), 'w'), indent=1)
    print('epoch accuracy', (pred == y).mean(), int((pred == y).sum()))


if __name__ == '__main__':
    main(sys.argv[1], sys.argv[2], int(sys.argv[3]) if len(sys.argv) > 3 else 2)
```

4.23. Python/verification/svm_metrics.py

SVM performance metrics and confidence intervals

```
#!/usr/bin/env python3
"""Metrics for the nested SVM evaluation and the CWImRMR-based tkNN evaluation:
confusion matrices,
participant votes, Wilson intervals and participant-cluster bootstrap intervals.

Usage: python svm_metrics.py file_sonuclari.mat nested_svm_pred.npy
nested_svm_folds.json out_dir
"""
import json
import os
import sys
from collections import Counter

import numpy as np
import scipy.io as sio

sys.path.insert(0, os.path.dirname(os.path.abspath(__file__)))
from analyze_file_mat import cm2, fold_order_ids, metrics, participant_summary,
participant_vote  # noqa

m = sio.loadmat(sys.argv[1])
y = m['y'].ravel().astype(int)
```

```
ids = m['newIndices'].ravel().astype(int)
pred = np.load(sys.argv[2])
folds = json.load(open(sys.argv[3]))


def cluster_boot(correct, groups, B=2000, seed=20261027):
    rng = np.random.default_rng(seed)
    u = np.unique(groups)
    idx = {g: np.where(groups == g)[0] for g in u}
    k = np.array([correct[idx[g]].sum() for g in u])
    n = np.array([len(idx[g]) for g in u])
    acc = []
    for _ in range(B):
        s = rng.integers(0, len(u), len(u))
        acc.append(k[s].sum() / n[s].sum())
    return [float(np.percentile(acc, 2.5)), float(np.percentile(acc, 97.5))]


e = metrics(cm2(y, pred))
rows = participant_vote(y, pred, ids)
p = participant_summary(rows)
e['cluster_boot_ci'] = cluster_boot((pred == y).astype(int), ids)
kinds = Counter(f['setting'][0] for f in folds)
lin_small = sum(1 for f in folds if f['setting'][0] == 'linear' and f['setting'][1] == '0.001')
settings = Counter(tuple(f['setting']) for f in folds)

# CWImRMR-based tkNN evaluation: bootstrap in fold order
actual = m['actual_labels'].ravel().astype(int)
ids_fold = fold_order_ids(actual, ids, y)
p3 = m['tahmin_loso'][0][2].ravel().astype(int)
mr_ci = cluster_boot((p3 == actual).astype(int), ids_fold)

out = dict(epoch=e, participant=p, kinds=dict(kinds), linear_C0001=lin_small,
        settings={str(k): v for k, v in settings.most_common()},
mrmr_verification_cluster_ci=mr_ci,
        inner_acc_mean=float(np.mean([f['inner_acc'] for f in folds])))
json.dump(out, open(os.path.join(sys.argv[4], 'nested_svm_metrics.json'), 'w'), indent=1,
default=float)
print('SVM epoch %d/%d = %.2f%%  sens %.2f spec %.2f bacc %.2f  CI %s' % (
    e['correct'], e['n'], 100 * e['acc'], 100 * e['sens'], 100 * e['spec'], 100 * e['bacc'],
    ['%.2f' % (100 * v) for v in e['cluster_boot_ci']]))
print('SVM participants %d/95 = %.2f%%  cm %s ties %s errors %s wilson %s' % (
    p['correct'], 100 * p['acc'], p['cm'], p['ties'], p['errors'], ['%.2f' % (100 * v) for v in
p['wilson_acc']]))
print('kernels', dict(kinds), 'linear C=0.001', lin_small, 'settings', settings.most_common(6))
```

```
print('CWImRMR cluster confidence interval', ['%.2f' % (100 * v) for v in mr_ci])
```

4.24. Python/verification/make_table_s1.py

Participant-level classification results (Supplementary Table S1)

```
#!/usr/bin/env python3
"""Supplementary Table S1: per-participant record of the nested SVM (fold-wise) and of the

import json
import os
import sys

import numpy as np
import scipy.io as sio

sys.path.insert(0, os.path.dirname(os.path.abspath(____)))
from analyze_file_mat import fold_order_ids  # noqa: E402

m = sio.loadmat(sys.argv[1])
res = sys.argv[2]
y = m['y'].ravel().astype(int)
ids = m['newIndices'].ravel().astype(int)
actual = m['actual_labels'].ravel().astype(int)
ids_fold = fold_order_ids(actual, ids, y)
p_knn = m['tahmin_loso'][0][2].ravel().astype(int)          # CWImRMR branch, fold order
p_svm = np.load(os.path.join(res, 'nested_svm_pred.npy'))    # y order
folds = {f['participant']: f for f in json.load(open(os.path.join(res, 'nested_svm_folds.json')))}
NAMES = {'linear': 'Linear', 'rbf': 'RBF'}

rows = []
for s in np.unique(ids):
    g = int(y[ids == s][0])
    n = int((ids == s).sum())
    k_ok = int((p_knn[ids_fold == s] == g).sum())
    k1 = int((p_knn[ids_fold == s] == 1).sum())
    k_vote = 1 if k1 >= n - k1 else 2
    s_ok = int((p_svm[ids == s] == g).sum())
    s1 = int((p_svm[ids == s] == 1).sum())
    s_vote = 1 if s1 >= n - s1 else 2
    f = folds[int(s)]
    kind, C, gam = f['setting']
    setting = f'{NAMES[kind]}, C = {C}' + ('' if gam == 'None' else f', γ = {gam}/p')
    rows.append(dict(id=int(s), group='Control' if g == 1 else 'Recorded offence', epochs=n,
                knn_correct=k_ok, knn_vote='Control' if k_vote == 1 else 'Recorded offence',
                knn_tie=k1 == n - k1, knn_ok=k_vote == g,
```

```
            svm_setting=setting,      svm_inner=round(100      *      f['inner_acc'],      2),
svm_correct=s_ok,
            svm_vote='Control' if s_vote == 1 else 'Recorded offence', svm_ok=s_vote == g))
json.dump(rows, open(os.path.join(res, 'table_s1.json'), 'w'), indent=1)
print('rows', len(rows), 'tkNN correct votes', sum(r['knn_ok'] for r in rows),
    'SVM correct votes', sum(r['svm_ok'] for r in rows))
print('tkNN epochs', sum(r['knn_correct'] for r in rows), 'SVM epochs', sum(r['svm_correct']
for r in rows))
```

4.25. Python/verification/make_figures.py

Figures 9, A3 and C1

```
#!/usr/bin/env python3
"""Figures generated from the classification and validation results.

Figure 9  : comparison of feature-selection methods (a) and IMV ensembles (b)       <-
file_results.json
Figure A3 : nested SVM confusion matrices (epoch, participant)          <-
nested_svm_metrics.json
Figure C1 : raw-epoch trace to the saved panels          <- archive + two raw epochs

Usage: python make_figures.py file_sonuclari.mat epoch_class1.mat epoch_class2.mat
results_dir out_dir
"""
import json
import os
import sys

import matplotlib
matplotlib.use('Agg')
import matplotlib.pyplot as plt  # noqa: E402
import numpy as np  # noqa: E402
import scipy.io as sio  # noqa: E402

sys.path.insert(0, os.path.dirname(os.path.abspath(____)))
from rhythmfluxpat import rhythmfluxpat  # noqa: E402

GREY, ORANGE, BLUE, DARK = '#8c8c8c', '#c55a11', '#1f4e79', '#1f2d3d'
plt.rcParams.update({'font.family': 'serif', 'font.size': 8.5, 'axes.spines.top': False,
            'axes.spines.right': False, 'savefig.dpi': 300, 'axes.titlesize': 9,
            'axes.titleweight': 'bold'})


def fig9(R, out):
  br = sorted(R['branches'], key=lambda b: b['rank'])[::-1]
```

```
fig, (a, b) = plt.subplots(1, 2, figsize=(7.2, 3.4), gridspec_kw={'width_ratios': [1.25, 1]})
ypos = np.arange(len(br))
single = [('∩' not in x['label']) for x in br]
a.barh(ypos, [100 * x['epoch']['acc'] for x in br], color=[BLUE if s else ORANGE for s in single],
       height=0.68)
a.scatter([100 * x['participant']['acc'] for x in br], ypos, s=22, facecolor='white', edgecolor='black',
          zorder=3, linewidth=1)
for y, x in zip(ypos, br):
    a.text(51, y, f'{100 * x["epoch"]["acc"]:.2f}', va='center', ha='left', color='white', fontsize=7)
a.set_yticks(ypos)
a.set_yticklabels([f'{x["label"]} ({x["n_features"]})' for x in br], fontsize=7.5)
a.set_xlim(50, 100)
a.set_xlabel('LOSO accuracy (%)')
a.set_title('(a) Feature-selection comparison', loc='left')
h = [plt.Rectangle((0, 0), 1, 1, color=BLUE), plt.Rectangle((0, 0), 1, 1, color=ORANGE),
     plt.Line2D([], [], marker='o', ls='', mfc='white', mec='black')]
a.legend(h, ['Single selector (epochs)', 'Intersection (epochs)', 'Participants'], fontsize=6.5,
         loc='upper center', bbox_to_anchor=(0.42, -0.17), ncol=3, frameon=False, handlelength=1.2,
         columnspacing=0.8)

I = R['imv']
k = [v['k'] for v in I]
acc = [100 * v['epoch']['acc'] for v in I]
sen = [100 * v['epoch']['sens'] for v in I]
spe = [100 * v['epoch']['spec'] for v in I]
ref = 100 * [x for x in R['branches'] if x['label'] == 'CWImRMR'][0]['epoch']['acc']
b.plot(k, spe, color=GREY, marker='s', ms=3.5, lw=1, label='Control specificity')
b.plot(k, acc, color=BLUE, marker='o', ms=4, lw=1.4, label='Accuracy')
b.plot(k, sen, color=ORANGE, marker='^', ms=4, lw=1, label='Recorded-offence sensitivity')
b.axhline(ref, ls='--', lw=0.9, color='black')
b.text(10.1, ref + 0.25, f'CWImRMR {ref:.2f}%', ha='right', va='bottom', fontsize=6.5)
for kk, v in zip(k, I):
    if v['epoch_vote_ties']:
        b.axvspan(kk - 0.35, kk + 0.35, color='#e6e6e6', zorder=0)
b.set_xticks(k)
b.set_xlabel('Branches pooled (top-k)')
b.set_ylabel('Epoch-level value (%)')
b.set_ylim(80, 100)
b.set_title('(b) IMV ensembles', loc='left')
hb, lb = b.get_legend_handles_labels()
hb.append(plt.Rectangle((0, 0), 1, 1, color='#e6e6e6'))
```

```
    lb.append('Tied votes (even k)')
    b.legend(hb, ['Specificity', 'Accuracy', 'Sensitivity', 'Tied votes (even k)'], fontsize=6.5,
             frameon=False, loc='upper center', bbox_to_anchor=(0.5, -0.17), ncol=4,
handlelength=1.2,
             columnspacing=0.7)
    fig.tight_layout()
    fig.savefig(os.path.join(out, 'figure9_selector_verification.png'))
    plt.close(fig)


def cm_panel(ax, cm, title):
    cm = np.array(cm)
    pct = cm / cm.sum(axis=1, keepdims=True)
    ax.imshow(pct, cmap='Blues', vmin=0, vmax=1.15)
    for i in range(2):
        for j in range(2):
            c = 'white' if pct[i, j] > 0.5 else DARK
            ax.text(j, i - 0.12, f'{cm[i, j]:,}', ha='center', va='center', fontsize=11, fontweight='bold',
                    color=c, family='sans-serif')
            ax.text(j, i + 0.2, f'{100 * pct[i, j]:.2f}%', ha='center', va='center', fontsize=8, color=c,
                    family='sans-serif')
    ax.set_xticks([0, 1])
    ax.set_xticklabels(['Control', 'Recorded-offence'])
    ax.set_yticks([0, 1])
    ax.set_yticklabels(['Control', 'Recorded-\noffence'])
    ax.set_xlabel('Predicted group')
    ax.set_ylabel('True group')
    ax.set_title(title)
    for s in ax.spines.values():
        s.set_visible(False)
    ax.tick_params(length=0)


def figA3(S, out):
    fig, (a, b) = plt.subplots(1, 2, figsize=(6.4, 2.9))
    cm_panel(a, S['epoch']['cm'], f'(a) Epochs (n = {S["epoch"]["n"]:,})')
    cm_panel(b, S['participant']['cm'], f'(b) Participants (n = {S["participant"]["n"]})')
    fig.tight_layout()
    fig.savefig(os.path.join(out, 'figureA3_nested_svm.png'))
    plt.close(fig)


def figC1(archive, e1, e2, out):
    m = sio.loadmat(archive)
    y = m['y'].ravel().astype(int)
```

```
sel = [m['selNCA'].ravel(), m['selCHI2'].ravel(), m['selMRMR'].ravel(), m['selRELIEFF'].ravel()]
S = m['sonlar'][0]
cols, feat = [], []
for k in range(4):
    for j, f in enumerate(sel[k]):
        if int(f) not in feat:
            feat.append(int(f))
            cols.append(S[k][:, j])
X = np.column_stack(cols)
feat = np.array(feat)
F = np.vstack([rhythmfluxpat(sio.loadmat(f)['blok_sinyal'])[0] for f in (e1, e2)])
df = F[1, feat - 1] - F[0, feat - 1]
sd = np.sign(df)
r2s = np.where(y == 2)[0]
best = []
for r1 in np.where(y == 1)[0]:
    a_ = (np.sign(X[r2s] - X[r1]) == sd).mean(axis=1)
    k = int(np.argmax(a_))
    best.append((a_[k], r1, r2s[k]))
best.sort(key=lambda t: -t[0])
_, r1, r2 = best[0]
dX = X[r2] - X[r1]
cross = (feat - 1) // 1024 >= 5
fig, (a, b) = plt.subplots(1, 2, figsize=(6.8, 2.8))
a.axhline(0, color='black', lw=0.6)
a.axvline(0, color='black', lw=0.6)
a.scatter(1e4 * df[~cross], dX[~cross], s=6, color=BLUE, alpha=0.7, label='Within-rhythm layers')
a.scatter(1e4 * df[cross], dX[cross], s=6, color=ORANGE, alpha=0.7, label='Cross-rhythm layers')
a.set_xlabel('Independently extracted difference (×10$^{-4}$)')
a.set_ylabel('Saved scaled difference')
a.set_title(f'(a) Matched pair: {int(round(best[0][0] * len(feat)))}/{len(feat)} signs agree', loc='left')
a.set_xscale('symlog', linthresh=1)
a.legend(fontsize=6.5, frameon=False, loc='upper left')
vals = np.array([t[0] for t in best]) * 100
b.hist(vals[1:], bins=np.arange(56, 101, 1.5), color=GREY)
b.axvline(vals[0], color=ORANGE, lw=2)
b.text(vals[0] - 1, b.get_ylim()[1] * 0.9, 'matched\npair', ha='right', fontsize=7, color=ORANGE)
b.set_xlabel('Best sign agreement per candidate pair (%)')
b.set_ylabel('Control rows')
b.set_title(f'(b) Next best: {vals[1]:.2f}%', loc='left')
```

```
    fig.tight_layout()
    fig.savefig(os.path.join(out, 'figureC1_raw_epoch_trace.png'))
    plt.close(fig)


if __name__ == '__main__':
    archive, e1, e2, res, out = sys.argv[1:6]
    os.makedirs(out, exist_ok=True)
    R = json.load(open(os.path.join(res, 'file_results.json')))
    S = json.load(open(os.path.join(res, 'nested_svm_metrics.json')))
    fig9(R, out)
    figA3(S, out)
    figC1(archive, e1, e2, out)
    print('figures written to', out)
```
